# Role of radicalization in the antioxidant and phototoxic properties of curcuminoids

**DOI:** 10.1039/d6ra07998a

**Published:** 2026-11-06

**Authors:** Giovanna Bruno, Maria Vittoria Rossi, Federico Vavassori, Angelo Maspero, Elisa Terzaghi, Damiano Monticelli, Andrea Penoni, Massimo Mella, Luca Nardo

**Affiliations:** a Dipartimento di Chimica, Biologia e Biotecnologie, Università degli Studi di Perugia Via Elce di Sotto, 8 Perugia (PG) 06123 Italy giovanna.bruno@unipg.it; b Dipartimento di Scienze Biomediche Sperimentali e Cliniche “Mario Serio” (DSBSC), Università degli Studi di Firenze Viale Morgagni, 50 Firenze (FI) 50134 Italy mariavittoria.rossi@unifi.it; c DiSAT – Dipartimento di Scienza e Alta Tecnologia, Università degli Studi dell'Insubria Via Valleggio, 9-11 22100 Como (CO) Italy fede.vavassoric3@gmail.com angelo.maspero@uninsubria.it elisa.terzaghi@uninsubria.it damiano.monticelli@uninsubria.it andrea.penoni@uninsubria.it massimo.mella@uninsubria.it luca.nardo@uninsubria.it +39 031 238 6273; d CLIP – Como Lake Institute of Photonics Italy

## Abstract

Curcumin exhibits antioxidant properties as well as phototoxic potential. Formation of curcumin radicals could be invoked to explain both features. In this work we present a systematic computational study on the tendency of curcumin to form radicals upon photosensitization to its excited singlet state. Concomitantly, experimental evidence of photoinduced curcumin radical production is provided through LC-HRMS and UV-visible absorption spectroscopy studies exploiting the radical-targeting spin-trap TEMPO. Because curcuminoids undergo extensive intramolecular hydrogen bonding, we investigate the role of hydrogen bonding patterns in fostering radical formation. Namely, we assess by TR-DFT calculations the differential proneness to radicalize through H-atom abstraction in correspondence of the enol and phenyl hydroxyl groups of curcumin and three substituted curcuminoids. The latter were chosen either to selectively prevent radicalization of each of the three hydroxyl moieties under quest or to hinder the formation of the H-bonds in which these moieties are involved in the parent compound. A mechanism potentially able to rationalize previous and current experimental results is, eventually, proposed, basing on the natural S_1_ → T_1_ intersystem crossing of photoexcited carbonyl groups, the T_1_ state being able to extract hydrogen atoms to form allyl-type radicals. These findings promise to open new perspectives towards full exploitation of curcuminoids photobiological activity.

## Abstract 2

We demonstrate photoinduced curcuminoid radical formation by mass spectrometry, unravel the underlying molecular mechanisms by all-atoms simulations and provide indication of stable C_α_ radicals based on UV-vis absorption and *in silico* calculations.

## Graphical abstract

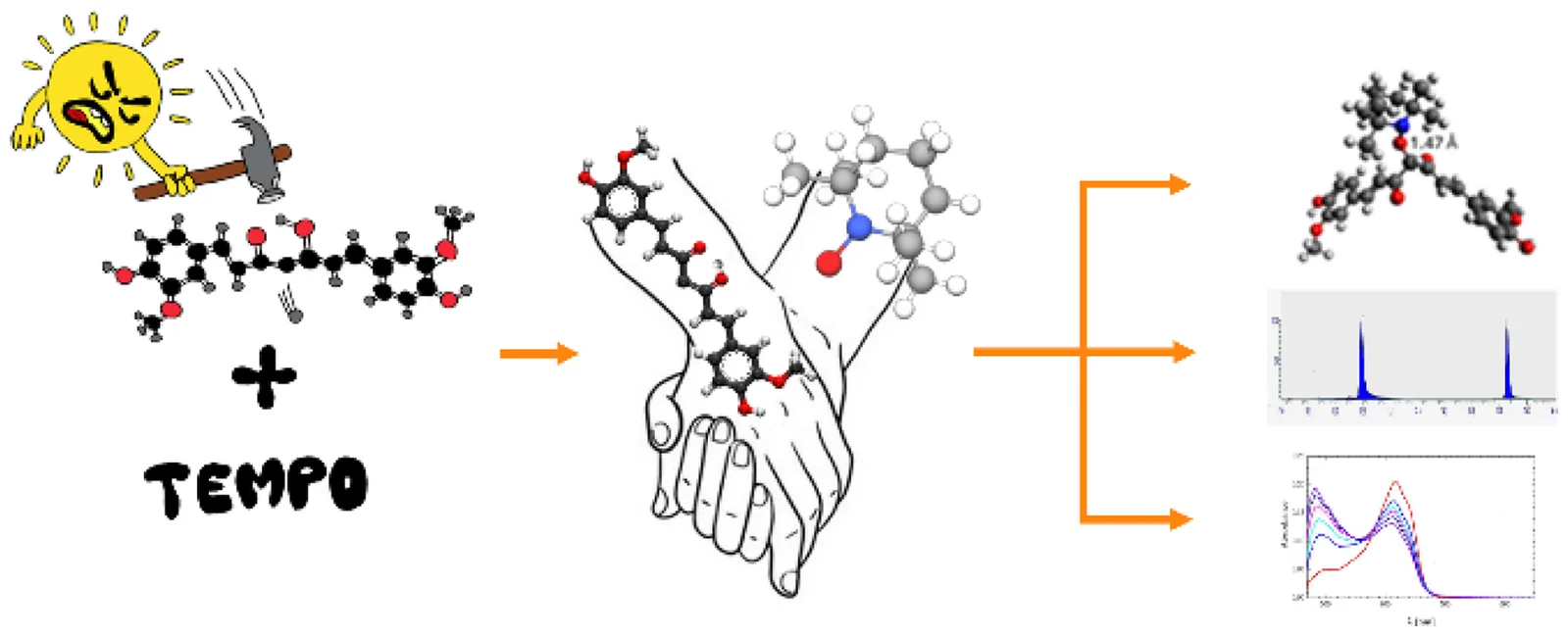

## 1 Introduction

Curcumin, the yellow-orange pigment extracted from the rhizome of *Curcuma longa*, has captured the interest of the scientific community since the seventies, when it was demonstrated that curcuminoids possess exceptional pharmacological properties, *e.g.* for treating hypercholesterolemia^1^ and diabetes,^2^ as natural anti-inflammatory^3^ and anti-oxidant^4^ agents and even as inhibitors of cancer proliferation.^5–7^ Later, curcumin has been demonstrated to have potential in the treatment of other severe pathologies including AIDS,^8,9^ Parkinson's^10^ and Alzheimer's diseases.^11,12^ Several reviews on the biomedical properties of curcumin and its analogues are available.^1,13–28^

Among the various aspects of curcumin bioactivity, the antioxidant potential has received special attention. In fact, it is well-known that oxidative stress is not only one of the main causes of aging through cell, proteins and DNA damage, but it is also strictly related to the development of several diseases, such as diabetes and different kinds of cancer and neurodegenerative pathologies. Thus, most of the several medicinal properties of this natural drug substance might eventually be ascribed to its antioxidant effects. A wealth of studies has been devoted to determining the antioxidant effects of curcumin and other curcuminoids, both natural and synthetic, assessed *in vitro*, *in cell* and *in vivo*, and focusing on different biologically relevant oxidative pathways. Direct activity as a scavenger of reactive oxygen species (ROS) and reactive nitrogen species (RNS) including superoxide anion, hydroxyl radicals, H_2_O_2_, singlet oxygen, nitric oxide, peroxynitrite, and peroxylradicals, was early demonstrated.^1,13,29–35^ However, indirect activity through modulation of proteins involved in ROS clearance has also been ascertained.^13,34–36^ As to the radical scavenging activity, in spite of the flourishing of several experimental studies between the end of the twentieth century and the first decade of the new millennium,^37–45^ a clear understanding of the underlying molecular mechanisms has not been achieved yet. Such controversy arises mainly because the mechanisms of interaction seem to depend strongly on both the environment and the targeted radical.^37,46,47^ Six different mechanisms, extensively described by Shaikh *et al.*^37^ and Galano *et al.*,^47^ have been proposed in the literature, namely:

- Single electron transfer (SET); by this mechanism, an oxidizing radical is transformed in an anion through donation of an electron by curcumin, which is concomitantly transformed to its monocation.

- H-atom transfer from neutral curcumin (HAT); by this mechanism, curcumin-OCH_3_ neutralizes oxidizing radicals through donation of one of its three acidic H atoms (either one of the phenolic hydroxyls or the enolic hydrogen). The result is a curcumin-OCH_2_^−^˙ radical.

- H-atom transfer from deprotonated curcumin (HAT-D); by this mechanism, curcumin first loses one proton. The curcumin monoanion OCH_2_^−^˙ then undergoes radicalization to OCH^−^˙ by transferring one of the remaining two H-atoms to an oxidizing radical, thereby neutralizing the latter.

- Sequential proton loss electron transfer (SPLET); in this case, the first step of the reaction is equal to HAT-D, but then, instead of dissociating an H-atom, the curcumin mono-anion OCH_2_^−^ transfers to the oxidizing radical an electron, transforming it into its mono-anion and forming a 
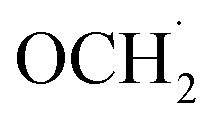
 radical.

- Radicals adduct formation (RAF); in this reaction, neutral curcumin forms a stable radical adduct with the oxidizing species.

- Sequential electron transfer-proton transfer (SET-PT); in this latter mechanism SET is followed by neutralization of the anion of the oxidizing species through transfer of a proton by OCH_3_^+^, resulting in formation of the curcumin radical 
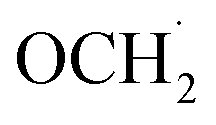
.

Interestingly, five of the above pathways encompass H-atom abstraction and formation of a curcuminoid radical as steps for neutralization of harmful oxidizing radical species.

Several theoretical studies have been devoted to elucidate the relative likelihood of the different radical neutralization pathways in terms of activation energy barriers.^37,38,46–50^ These efforts have brought to some agreement in the physical chemists' community. Although SET has been considered to have a role at basic pH or in the presence of electron donating substrates (see *e.g.* Jin *et al.*,^35^ Shaikh *et al.*,^37^ Galano *et al.*,^47^ Gorman *et al.*^51^), the energetically favoured process in physiological conditions seems to be H-atom abstraction, with the concomitant generation of a curcuminoid radical.^35,37–43,46–51^ An additional caveat concerns the actual radicalization site. Several works evidenced that the bond dissociation enthalpy (BDH) for the phenolic H atoms abstraction is significantly lower than that required to remove the enolic/vinylic H atom thus forming directly a carbonyl radical.^37,38,46–48,50^ However, this result has been put into doubt by some authors.^49^ Moreover, it seems that the high degree of conjugation of the curcuminoid molecule promotes fast resonance between the two radical species, and some evidence has been produced suggesting that in the end the more stable radical species is actually the carbon-centred one,^47,50,52^ although Priyadarsini and coauthors propend for the formation of stable hydroxyl radicals.^37,38^

Despite the above strong conjectures pointing to formation of curcuminoid radicals as a pivotal step underlying their antioxidant activity, relatively limited attention has been granted to detecting and characterizing such radicals. Indeed, there exist only few studies claiming for evidence in support of the actual existence of curcuminoid radical species. In 1994, Chignell *et al.* reported the detection of “unidentified carbon-centered radicals” in curcumin samples irradiated with lamp light using the electron paramagnetic resonance/spin-trapping technique with 5,5-dimethyl-pyrroline-*N*-oxide as a trapping agent.^53^ Schaich and coworkers also applied electron paramagnetic resonance, with 4-POBN as a spin trap, to unravel the formation of a curcuminoid radical: based on the splitting constant values, they found that adducts were typical of an alkyl rather than a phenyl radical and concluded that the latter were localized at the vinylic carbon of the central diketo moiety.^39^ Concomitantly, Gorman *et al.*, by means of pulse radiolysis experiments, detected a long-lived transient and associated it with the formation of a curcumin radical; they further speculated that such radical was generated through HAT at the phenolic hydroxyl.^51^ Several years later, two pivotal studies by Jovanovic *et al.* exploited pulse radiolysis and flash photolysis to identify the appearance of a transient absorption band at ≈500 nm, concomitant to bleaching of the ground state to first excited singlet state transition band in curcumin and some analogues and attributed it to formation of a curcuminoid radical.^40,42^ This finding was confirmed independently by Priyadarsini and collaborators, who also found an additional transient absorption band in the UV and concluded that two distinct radical species were coexisting; this conclusion was corroborated by cyclic voltammetry experiments, revealing two distinct, though closely lying, oxidation peaks. Also, the authors compared the behavior of curcumin and dimethoxycurcumin, a synthetic analogue lacking phenolic hydroxyls: for this derivative, only one oxidation peak and the UV transition band were observed.^38^

Another very captivating feature of curcumin and its analogues lies in their notable phototoxicity.^54–56^ This has been ascertained in 1989 by Dahl *et al.*^57^ and several studies have been devoted to probe the efficacy of the parent compound as well as natural and specifically designed synthetic curcuminoids on several cell lines, including cancer models and bacteria.^58–63^ The IC50 values span in the range of hundreds of nanomolar to low micromolar concentrations, suggesting the potential of curcuminoids as active principles for both the photodynamic therapy of tumours and bactericidal/antibiotic purposes. Similarly to their antioxidant properties, also the molecular bases of the photosensitizing activity of curcuminoids remain elusive. Although a recent publication by Wolnicka-Glubisz *et al.* seems to re-evaluate the role of direct singlet oxygen (^1^O_2_) production as a main mechanism of photodamage by curcumin,^64^ it was early recognized that the intense phototoxic activity of curcumin could not be reconcealed with a classical type-1 or type-2 photosensitization of ^1^O_2_ through intersystem crossing, due to the very low quantum yield of this ROS.^51,58^ It was thus proposed that formation of excitonic curcumin radicals might be at the origin of its photokilling potency through triggering of a ROS cascade.^39,51,58,59^ The excited state dynamics of curcuminoids, however, has proved to be extremely complex, and to depend on the photosensitizers' environment as well as their tendency to form intra- and inter-molecular H-bonding patterns.^54,65–70^ For this reason, until now the interesting hypothesis of light-mediated curcuminoid radicals' production as the main source of the photobiological activities of this class of compounds has remained at the speculative level, and to the best of our knowledge photosensitized curcuminoid radicals were never isolated.

In this article, with the aim of offering a rationalized panorama on the mechanisms of curcumin radical formation through H-atom abstraction, we first present a systematic study, based on TD-DFT calculations, on H-atom bond dissociation energies (BDE) of the three acidic protons in the ground state. In order to probe the dependence of BDE from the complex intramolecular H-bonding patterns exhibited by curcumin, the study is extended to a number of curcuminoids in which the H-bonds involving the abstractable protons are selectively perturbed one at a time. Attention is devoted also to the tuning of BDE due to the physicochemical properties of the environment. Namely, polarity and H-bonding affinity of the solvent are mimicked by both implicit and explicit solvent models.

Since in the literature we could only find studies aimed at determining the BDE/BDH for HAT from curcumin and analogues in the ground state, which appear to be valuable for assessing the antioxidation mechanisms but are unfit to investigate the molecular bases of the molecule's photosensitized pharmacological activities, we then focus our analysis to the calculation of BDE for HAT from the first excited singlet state of curcumin, showing that the excitation energy is more than sufficient to overcome the pertaining HAT potential barrier. The results yielded by these calculations are complemented by UV-vis absorption and LC-HRMS experiments in which curcumin radicalization is photosensitized in a deoxygenated environment in the presence of the radical targeting spin-trap (2,2,6,6-tetramethylpiperidinyl-1-yl)oxyl TEMPO. Both techniques show unambiguously that curcumin–TEMPO adducts are formed, thereby demonstrating photosensitized generation of curcumin radicals. A mechanism for excited-state curcumin radical production is proposed based on the natural S_1_ → T_1_ intersystem crossing of photoexcited carbonyl groups, the T_1_ state being able to extract hydrogen atoms to form allyl-type radicals. Finally, the calculated proneness to radicalization of the different curcuminoids is compared to their relative ability to produce singlet oxygen, as assessed by means of the 9,10-dimethylanthracene fluorescence assay. These results demonstrate that curcuminoid radicals can be generated through spontaneous excitonic reactions upon photosensitization, shedding new light on the molecular bases of curcuminoids photobiological activity.

## 2 Materials and methods

### 2.1. Computational details of theoretical investigation

Geometry optimizations of compounds in Fig. 1 and corresponding radicals in both the ground and excited state were carried out at the DFT level of theory (Time Dependent (TD) version of the method was employed for the excited state calculations). The optimized geometries are shown in SI (see Section S0). In regard to the ground state we employed the hybrid functional B3LYP and the Pople triple-zeta basis set plus polarization functions 6-311G(d,p), whose conjunction has proven to provide accurate results for the structures of acenes.^71^ The same level has been shown to be of comparable accuracy with other functionals with respect to bond dissociation energies for highly conjugated species such as quercetin and edaravone.^72^ Notice that we have, in general, not included semi-empirical dispersion corrections in our calculations, as these do not appear to impactfully change the overall isomeric energy landscape (*e.g.* see the paper by Madinah *et al.*,^73^ as well as Section S2 of the SI for the test we run with respect to relative isomer energetics and bond dissociation energy). Whereas for the excited state calculations we reduced the cardinality of the basis set, employing 6-31G(d,p) to speed up the calculations. From a literature analysis, it seems that neither the selected functional nor the basis set should be expected to impact heavily on the accuracy of the computational results. In fact, the localized excitonic nature of the S_1_ state (*e.g.* see the article by Ghiami-Shomami *et al.*^74^) has been found to markedly reduce the importance of long-range correction, which are instead much needed for charge-transfer excitations.^75^ Moreover, standard TD-B3LYP has already been shown to be a useful approach in deciphering experimental data involving curcumin-related radicals basing on the comparison between *in silico* and experimental spectra.^76^

**Fig. 1:**
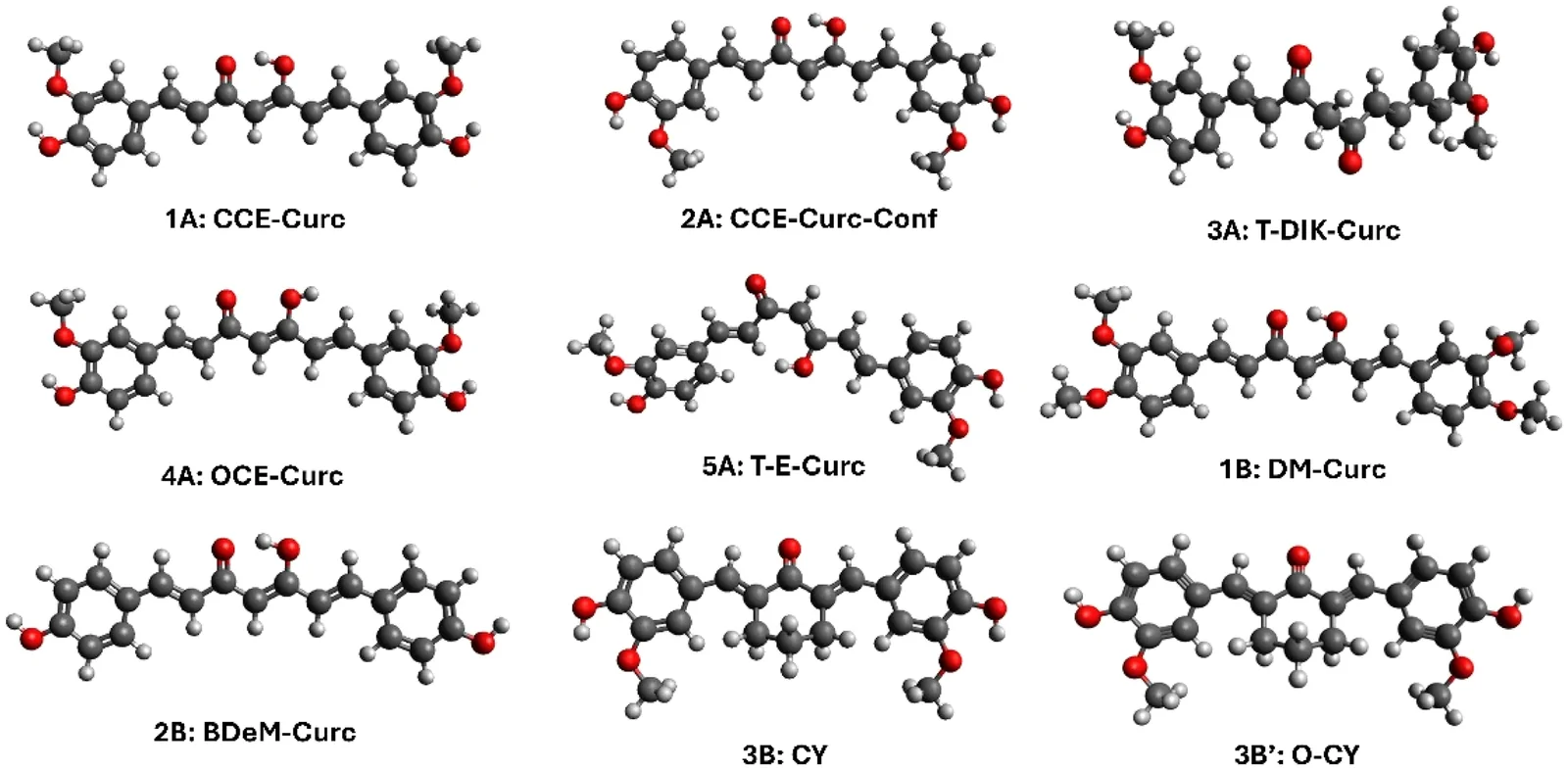
Curcuminoids analyzed in this work. Group A: 1A: CCE-Curc: Closed Cis Enol Curcumin; 2A: CCE-Curc-Conf: Closed Cis Enol Curcumin Conformer; 3A: T-DIK-Curc: Trans Diketo Curcumin; 4A: OCE-Curc: Open Cis Enol Curcumin; 5A: T-E-Curc: Trans Enol Curcumin. Group B: 1B: DM-Curc: Dimethoxy Curcumin; 2B: BDeM-Curc: Bis-Demethoxy Curcumin; 3B: CY: Cyclovalone; 3B′: O-CY: Cyclovalone with phenolic hydroxyl groups in open conformation.

The same methodologies were then employed to perform the analytical frequencies analysis which confirmed that the obtained geometries correspond to local minima; this calculation also yields the thermodynamic variables and the zero-point energy. Unless otherwise specified, all calculations were performed by Gaussian09, revision B.01.^77^ Cartesian coordinates of the stationary points optimized can be obtained by the authors upon request.

The curcuminoid radicals' analysis (Section 3.1.3) was carried out by employing the same level of theory (B3LYP/6-311G(d,p)) for all calculations. Concerning the spin density, it was computed by employing the pop:noab keyword of Gaussian09 and then its QTAIM populations were computed by using the Multiwfn program package.^78,79^

### 2.2. Liquid chromatography high resolution mass spectrometry (LC-HRMS)

Analyses of the samples were performed using an Ultra High Performance Liquid Chromatography (UHPLC) device (Thermo Fisher Vanquish) coupled to a mass spectrometer (Thermo Fisher Orbitrap Exploris 120) operating in ESI negative ion full scan MS (100–800 u) at 60 000 resolution and in data dependent MS2 at 30 000 resolution and 30–60 NCE collision energies. The ionization conditions were optimized for acidic compounds and resulted in the formation of negative molecular ions [M − H]^−^. The chromatographic separation was performed with an Accucore C18 column, 50 × 2.1 mm ID, 2.6 µm phase (Thermo Fisher) using a gradient of ammonium acetate, 10 mM in water (A) and acetonitrile (B) at a flow rate of 350 µL min^−1^. The chromatographic gradient was as follows: initially solvent A was set at 99% while B was set at 1%; B was then linearly increased to 99% in 19 min and kept constant for 10 min; then B was brought to 1% in 1 min, while A was set at 99% and kept constant for 6 min to equilibrate the column (total run time 36 min). Chromatograms obtained from the LC-HRMS analysis were analyzed with the Freestyle software (Thermo Fisher Scientific) for target analyses of curcumin and suspect screening of curcumin + TEMPO based on their exact masses. Extracted ion chromatograms (XIC) were extracted from full scan data, with a tolerance range of ±5 ppm. The exact masses of the deprotonated form of the detected compounds are reported in Table 1.

**Table 1:** Curcumin and curcumin–TEMPO adduct monitored masses

Species	Formula	Exact mass	[M − H]^−^
[Curcumin]	C_21_H_20_O_6_	368.12599	367.11871
[Curcumin–TEMPO] adduct	C_30_H_37_NO_7_	523.25700	522.24973

### 2.3. UV-vis absorption measurements

All the UV-vis absorption measurements were performed on a Lambda2 spectrophotometer (PerkinElmer). As it is well known that ^1^O_2_ reacts with TEMPO to form a stable and neutral adduct, in order to monitor formation of curcumin radical adducts with TEMPO the solutions were previously deoxygenized under argon flux. Namely, the solvents in which the TEMPO and curcumin stock solutions were prepared were preliminarily degassed with argon for 1 h to remove oxygen gas. Then suitable quantities of powdered compounds were added to obtain 1 mM concentrated TEMPO and 10 µM concentrated curcumin stocks. The latter were further gurgled for 10 min under argon flux. The cuvettes used for the UV-vis absorption measurements (Hellma) were made of Suprasil quartz and they were hermetically sealed by screw-tips equipped with PTFE membranes. They were initially filled with the previously degassed solvent of interest. The latter was gurgled for 10 min in order to remove oxygen from the cuvette walls. The solvent was eliminated, and the cuvettes were filled with freshly degassed solvent. Curcumin and/or TEMPO stocks were added to get solutions with final concentration of 1 mM TEMPO (cuvette directly filled with the stock solution) and/or 1 µM curcumin. The measurements were executed readily after sample preparation. Throughout the preparation and degassing procedures, all samples were kept in the dark by wrapping the glassware in aluminum foils.

A Wood lamp (Kunpeng; 220–240 V, 50–60 Hz, 26 W) was used to photosensitize the samples. The sample containing both curcumin and TEMPO and the control solutions containing only TEMPO and only curcumin, respectively, were irradiated simultaneously using an expressly built illumination chamber, assuring deliverance of homogeneous light doses.

### 2.4. Measurement of ^1^O_2_ generation efficiency

The relative efficiency of curcumin, dimethoxycurcumin, bisdemethoxycurcumin and cyclovalone in producing ^1^O_2_ has been tested in spectroscopic grade pure acetonitrile, dimethylsulfoxide, and methanol (Merck) by applying the 9,10-dimethylanthracene fluorescence assay. In order to prevent oxidation of the fluorescent sensor prior to interaction with photosensitized curcuminoids, stocks of 9,10-dimethylanthracene at 1 mM concentration in deoxygenated solvents were prepared. Namely, the solvents were gurgled with argon for 30 minutes. Powdered 9,10-dimethylanthracene, purchased from Sigma Aldrich, was added, the samples were sonicated for 5 minutes to allow complete solubilization and further gurgled with argon for 5 minutes. Stocks (100 µM) of the curcuminoids in deoxygenated solvents were also prepared according to the same procedure and kept in the dark until use. The samples used for the measurements were prepared immediately after usage starting from the stocks and from non-deoxygenated solvents, using the above-described hermetically sealed screw-tip quartz cuvettes. The final curcuminoid concentration was 1 µM and the 9,10-dimethylanthracene concentration was 10 µM in all the samples. Samples of pure 9,10-dimethylanthracene in the absence of photosensitizers and of pure curcuminoids in the absence of the fluorescent sensor were also prepared. The fluorescence spectrum in the range 380–600 nm was measured by means of a PTI fluorescence master system spectrofluorimeter upon excitation at 340 nm for all the samples. Subsequently, the solutions were irradiated by means of the Wood lamp (see Section 2.3 for details) for 30 s and their emission spectra re-acquired under the same instrumental settings. The spectra were automatically corrected with respect to wavelength dependences of the photomultiplier tube detection quantum efficiency by the acquisition software (Felix2000). The amount of generated singlet oxygen was quantified in terms of the spectrum-integrated fluorescence quenching. Namely, using eqn (1):1
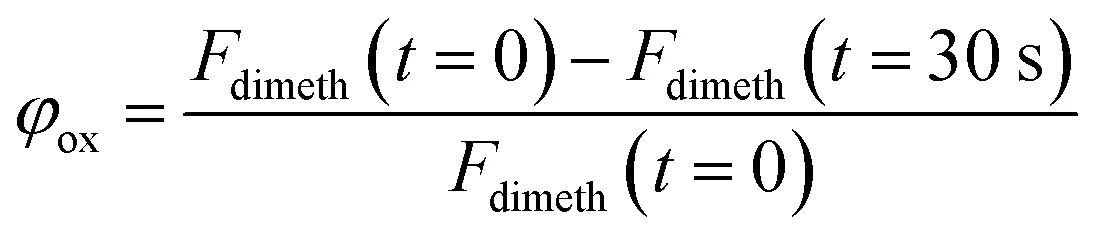


## 3 Results and discussion

### 3.1. Theoretical investigation

As suggested by several experimental as well as theoretical studies on the topic, we focused on the HAT mechanism as the main responsible of curcuminoids radicalization, which, depending on the circumstances, may foster the antioxidant activity and photosensitizing potential of these molecules. As depicted in Scheme 1, the antioxidation mechanism proceeds by transferring a hydrogen atom from the curcuminoid to a free radical, resulting in the quenching of the latter and in the formation of the corresponding curcuminoid radical. As to the photosensitized generation of ROS, irradiation is hypothesized to bring curcumin to its first excited singlet state, and the excitation energy can then be used to trigger an excitonic HAT with formation of the curcumin radical. Eventually, the latter interacts with the substrate to generate a cascade of ROS.

**Scheme 1:**
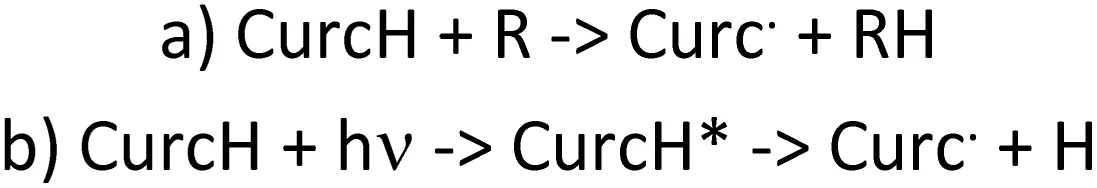
Hydrogen Atom Transfer (HAT) mechanisms (a) in the ground state by interaction with oxidant species of the substrate and (b) in the excited state using energy from the light field.

Curcumin radicalization *via* HAT mechanism may involve either of the hydroxyl moieties at the phenol or enol groups, which can form intramolecular H-bonds with neighboring groups. Therefore, it is reasonable to assume that the thermodynamic efficiency of curcuminoids radicalization does not merely depend on the strength of the O–H chemical bond but also on the strength of the H-bonds which the hydroxyl groups may form. Quite obviously not only the specific structural isomer and corresponding conformers but also possible substituents to either the carbon chain or the aromatic rings may affect the reactivity. All these factors were analyzed by selecting a sample of nine curcuminoids divided into two groups: curcumin isomers (Group A) and curcumin derivatives (Group B) (Fig. 1).

As regards the theoretical part of this work, the antioxidant power of curcuminoids and its relationships with the intramolecular H-bonding patterns were evaluated from a thermodynamic perspective. More specifically, the BDE associated to all hydroxyl moieties of curcuminoids was estimated in the ground state (S_0_) and, in a few cases, in the first excited singlet state (S_1_) *via*eqn (2), where *E*_H_ is the total energy of the hydrogen atom, *E*_Curc_ the total energy of the curcuminoid before radicalization (in the S_0_ or S_1_ states) while *E*_Curc˙_ the corresponding value after the reaction (in the D_0_ or D_1_ state, respectively). In fact, we are going to show later that the S_0_ → S_1_ transition in curcuminoids mainly involves HOMO and LUMO orbitals of the S_0_ state, which are highly delocalized over the conjugated chain and aromatic rings. Consequently, it is reasonable to assume that radicalization proceeds by passing through the D_1_ state and that the latter presents a reasonably long lifetime.

2 BDE = (*E*_Curc˙_ + *E*_H_) − *E*_Curc_

Hereafter, we will refer to the right and left lateral radicals as the right and left radical forms obtained from the radicalization on the right and left phenolic hydroxyl groups, using the labels R-Lat-Ph and L-Lat-Ph, respectively. Conversely, we will refer to the central radical as the radical form obtained from the radicalization of the central enolic hydroxyl group, using the label C-En. Note that in the case of compound 3A: T-DIK-Curc which does not have any enolic hydroxyl group, we also simulated the radicalization on the central –CH_2_ moiety connecting the two carbonyl groups (radical indicated as C–CH).

#### 3.1.1. Conformational analysis of curcumin

To determine the relative abundance of curcumin isomers, we carried out a conformational analysis of curcumin tautomers and conformers, shown in Fig. 1, with the chosen electronic structure methodology. The results of the latter study appear to be in agreement with the ones present in previous literature on the molecules, so that, for the sake of conciseness, we provide them and the associated discussion in Section S1 of the SI. For the overall goal of this work, it is, nevertheless, important to report that CCE-Curc (*vide*Fig. 1) appears to be the most stable isomer, even when including implicit solvent models or explicit solvent molecules to mimic solvation in acetonitrile or methanol. The potential impact of explicit solvent molecules onto the CCE/OCE isomerization has also been investigated and discussed in the SI under Section S1.

#### 3.1.2. Bond dissociation energy (BDE) of O–H fragments in curcuminoids

As anticipated, the antioxidant capacity of curcuminoids *via* HAT mechanism and its dependence on the strength of intramolecular H-bonds was evaluated in terms of BDE (eqn (2)) associated with the homolytic breaking of O–H fragments. The BDEs shown in Fig. 2 indicate that, regardless of the curcuminoid, a clear distinction exists between the proneness of undergoing radicalization of phenolic and enolic O–H groups whenever an intramolecular keto–enol H-bond is present (compounds 1A, 2A, 1B and 2B). Namely, the enolic O–H shows BDEs (∼106) around 20 kcal mol^−1^ higher than the phenolic ones (∼86 kcal mol^−1^). Interestingly, this trend stands whether or not the phenolic groups form intramolecular H-bond with neighboring groups such as in all curcumin isomers and both 3B and 3B′ curcumin derivatives. This suggests that phenolic intramolecular H-bonds do not significantly influence the reactivity of the corresponding O–H moieties while confirming the especially high stability of keto–enol intramolecular H-bond discussed in previous sections.

**Fig. 2:**
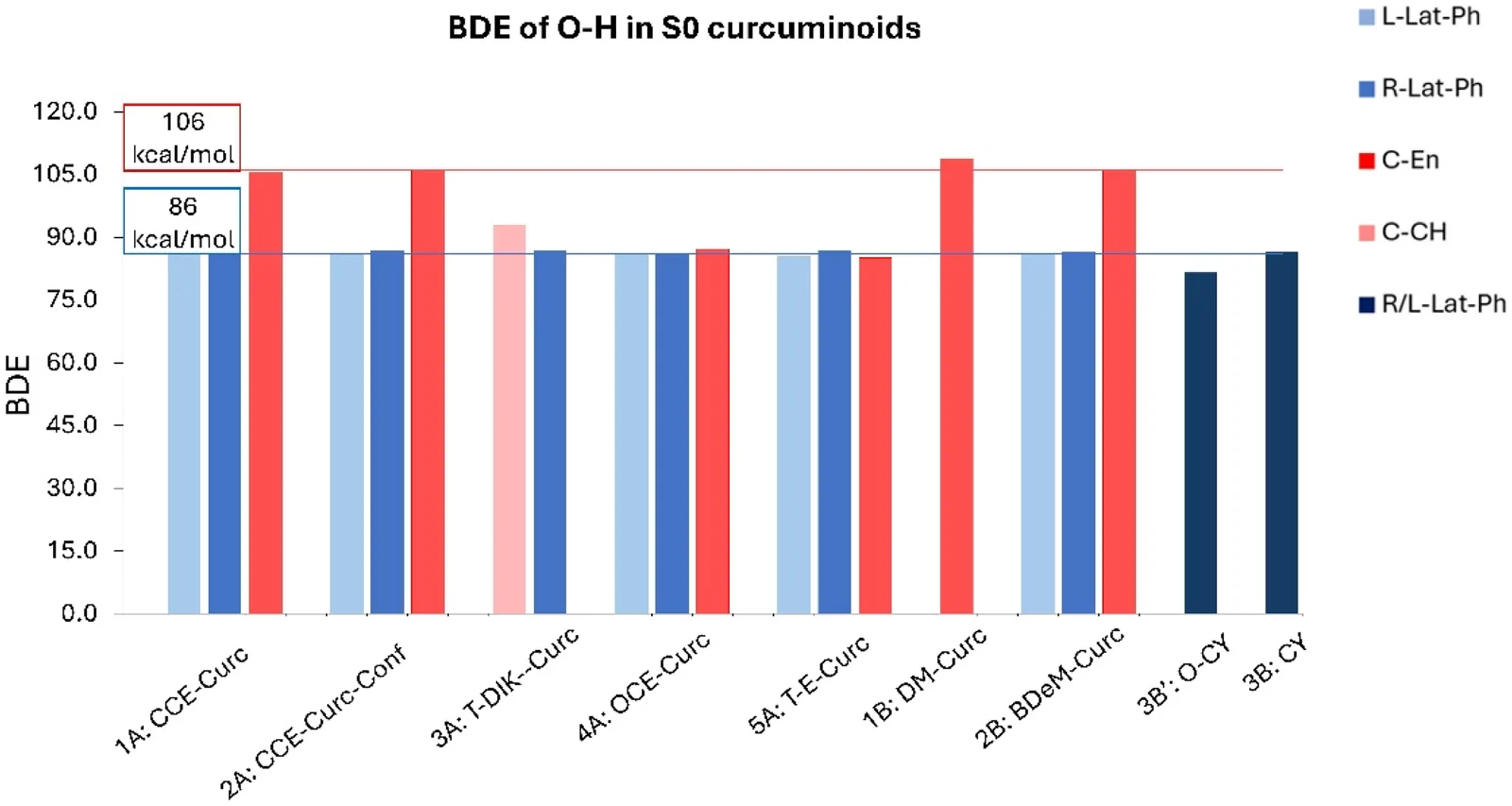
BDE of enolic and phenolic hydroxyl groups of the curcuminoids of Fig. 1 in the singlet ground state (S_0_). Note that in case of compound 3A: T-DIK-Curc which does not present any enolic hydroxyl group, we also simulated the breaking of the C–H bond of the central methylene group. As shown in the legend, light blue bars, blue bars and red bars refer to the O–H breaking of left phenolic groups, right phenolic groups and enolic groups, respectively. The pink bar refers to the breaking of C–H in the central methylene group of compound 3A: T-DIK-Curc while dark blue bars refer to either left or right phenolic O–H breaking when the latter basically show the same BDE. All values are reported in kcal mol^−1^.

It is also noteworthy that despite the different chemical environment of enolic and phenolic groups, the corresponding BDEs have similar values when intramolecular keto–enol H-bond does not form as in compounds 4A and 5A. This corroborates the argument according to which the less favorable thermodynamics of enol radicalization is basically due to the exceptional strength of the RAHB of keto–enol moiety. In fact, the latter results so thermodynamically stable that even the C–H bond breaking of methylene in compound 3A shows a lower BDE (around 93 kcal mol^−1^).

In general, given a specific reaction site, Fig. 2 clearly suggests that no significant differences should be expected in the thermodynamics of radicalization of curcumin isomers and derivatives.

The values of BDE estimated herein are in good agreement with literature values^37,38,46–50^ and suggest that any of the analyzed curcuminoids can act as an effective antioxidant with respect to, *e.g.*, most physiologically produced ROS according to the reaction sketched in Scheme 1a.^46,80^ However, it is likely that the transferred protons are, when present, the phenolic ones due to their lower BDE, which is likely to correlate with lower activation barriers.^81,82^ This notwithstanding, the enol proton can also participate in the radical scavenging of, *e.g.*, hydroxyl radicals (water BDE = 119 kcal mol^−1^). Conversely, the BDEs are so remarkably high, ranging between 80 kcal mol^−1^ and 110 kcal mol^−1^, to suggest that the self-cleavage of the O–H bonds could only occur at extremely high temperature and thus under rather extreme experimental conditions; thus, the latter process cannot be the root-cause of a radical cascade onset.

In order to investigate the mechanisms of photosensitization relying on formation of curcuminoid radicals starting from the molecule excited state, summarized in Scheme 1b, we also investigated the thermodynamics of radicalization of the first excited singlet state (S_1_) of a subset of the curcuminoids of Fig. 1. In particular, since we did not observe relevant differences among curcumin isomers and derivatives in the singlet ground state (S_0_), the S_1_ BDEs were only computed for the two conformers of closed cis enol curcumin (compounds 1A: CCE-Curc and 2A: CCE-Curc-Conf in Fig. 1) and the corresponding conformers of cyclovalone, a curcuminoid in which the diketo moiety is substituted by a pentose ring (compounds 3B: CY and 3B′: O-CY in Fig. 1). Fig. 3 clearly shows that the BDEs of hydroxyl groups significantly decrease upon excitation of curcuminoids to state S_1_. More specifically, the BDEs of enolic hydroxyl moieties lower by around 50 kcal mol^−1^ while the ones of phenolic hydroxyl groups by around 30 kcal mol^−1^. As a consequence, the high energy gap (around 20 kcal mol^−1^) between enolic and phenolic groups involved in intramolecular H-bonds (see Fig. 2, compounds 1A, 2A and 2B), which would favor the radicalization of phenolic groups in the S_0_ state, basically disappears in the S_1_ state where all BDEs range around 50 kcal mol^−1^. However, in both cases the O–H intramolecular bond distance not only decreases, suggesting a strengthening of the corresponding bonds, but it does so to the same extent that is around 0.01 Å. In addition, no noticeable differences are observed in the overall geometry of the compounds in the states S_0_ and S_1_. To further investigate the role of intramolecular H-bonds in determining the more favorable thermodynamics of radicalization in the S_1_ state, we also computed the variation of the wave function square modulus associated with the S_0_ → S_1_ excitation.

**Fig. 3:**
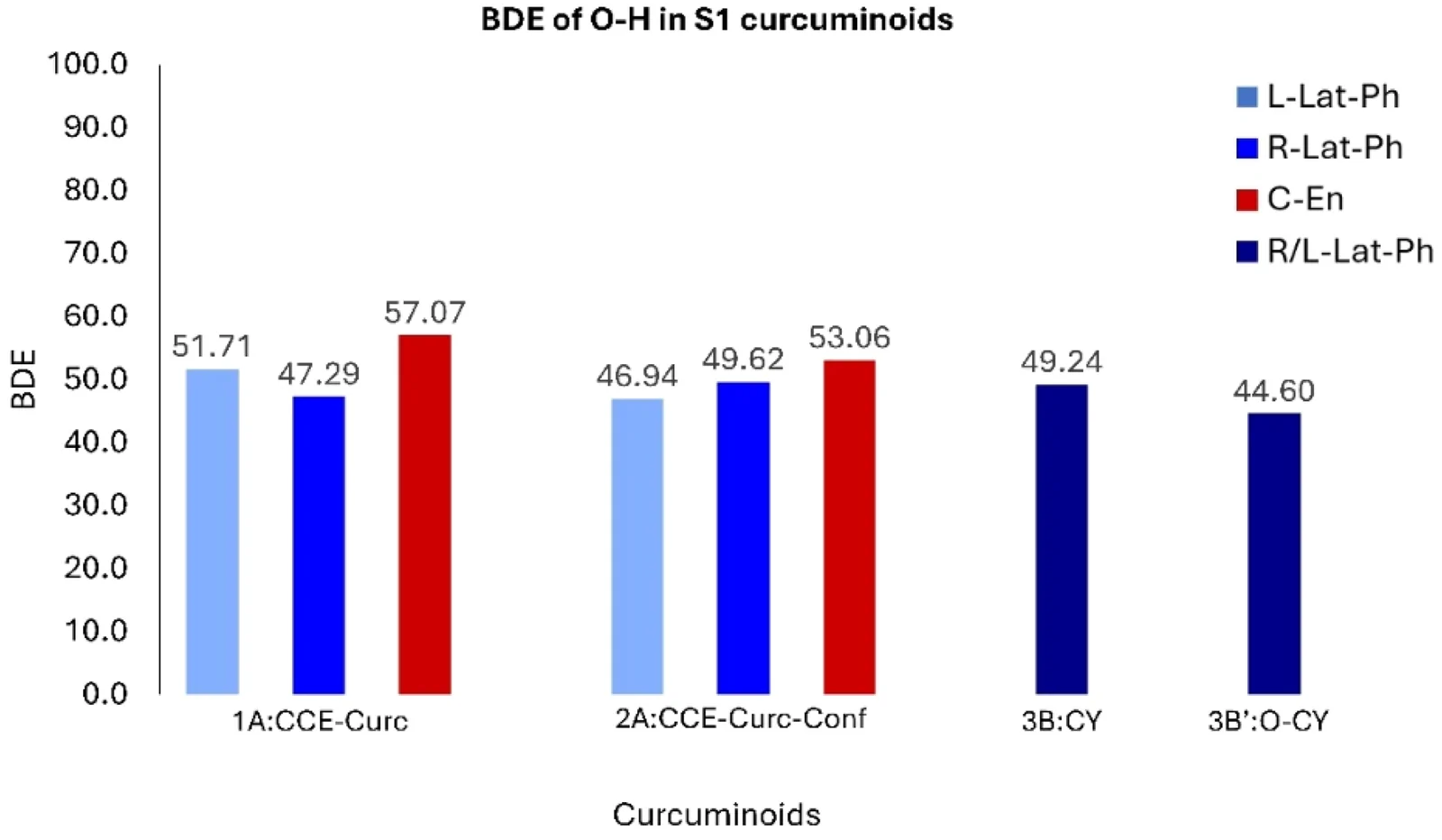
BDE of enolic and phenolic hydroxyl groups of compounds 1A: CCE-Curc, 2A: CCECurc-Conf, 3B: CY and 3B′: O-CY in the first excited singlet state (S_1_). As shown in the legend, light blue bars, blue bars and red bars refer to the O–H breaking of left phenolic groups, right phenolic groups and enolic groups, respectively. Dark blue bars refer instead to either left or right phenolic O–H breaking when the latter basically show the same BDE. All values are reported in kcal mol^−1^.

Since the HOMO–LUMO single particle transition accounts for around the 70% of the orbital composition associated with the excitation, such variation is well-approximated by the difference between the S_0_ HOMO's and LUMO's square moduli shown in Fig. 4. These results basically confirm the marginal role of intramolecular H-bonds in determining the thermodynamics of radicalization in the S_1_ state since they do not contribute to the modification of the electronic structure of compound 1A: CCE-Curc upon excitation.

**Fig. 4:**
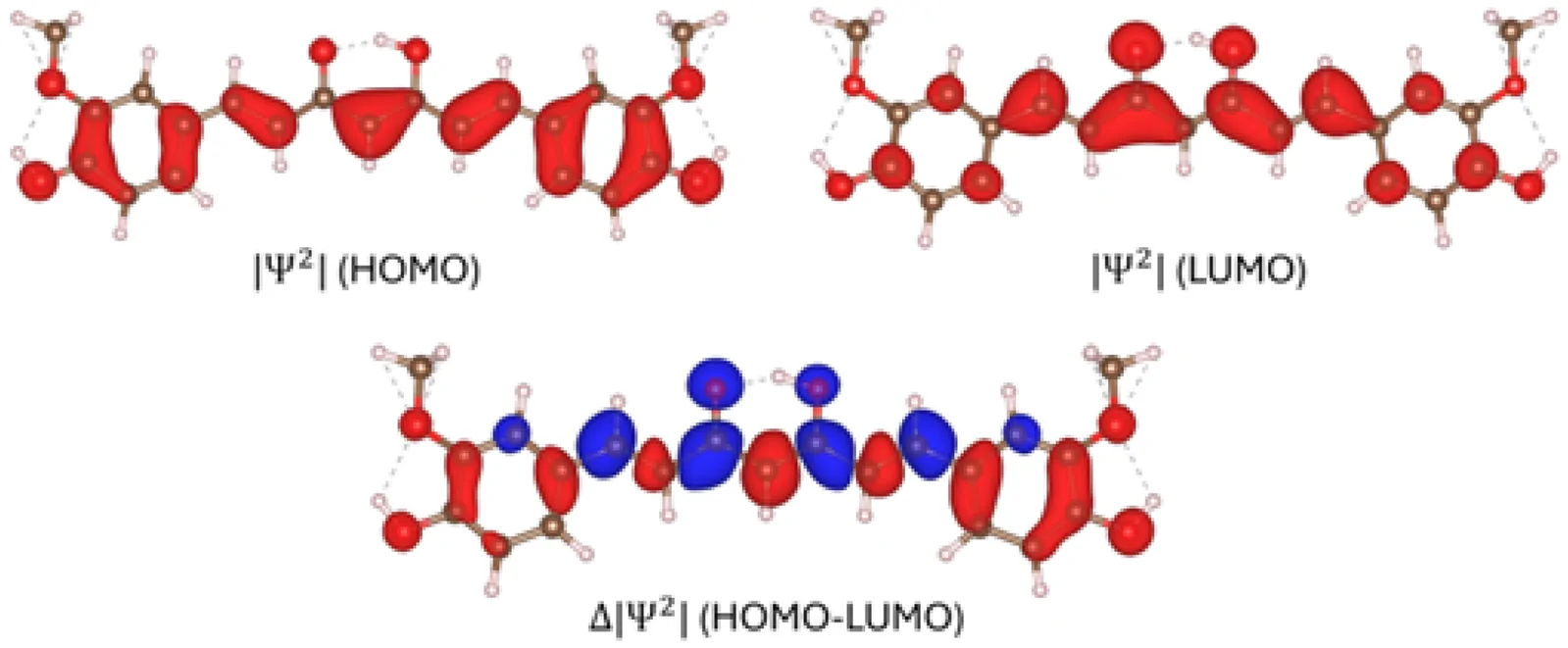
Square modulus of S_0_ HOMO and LUMO's wavefunctions and their corresponding difference.

Therefore, neither the weakening of intramolecular H-bonds nor eventual geometry relaxations seem to justify the substantially more favored thermodynamics of radicalization as well as the leveling of phenolic and enolic groups reactivity in the excited state. Hence, these data collectively suggest that the more favorable thermodynamics of curcuminoids radicalization in the S_1_ state is mainly due to the stabilization of their radicals in the excited state rather than the destabilization of the reagents.

#### 3.1.3. Characterization of curcuminoids radicals

In order to better characterize the curcuminoids radicals we computed both the electron spin density and the thermodynamics of their scavenging with the well-known radical marker 2,2,6,6-tetramethylpiperidinyl-1-oxyl (TEMPO). Since no significant differences were detected in the thermodynamics of radicalization of different curcumin isomers and derivatives (Fig. 2), herein we only report the results associated with the reference molecule 1A: CCE-Curc.

Fig. 5 shows the QTAIM electron spin density populations associated with selected atoms of both D_0_1A: CCE-Curc (C-En) and D_0_1A: CCE-Curc (R-Lat-Ph) (results comparable to D_0_1A: CCE-Curc (R-Lat-Ph) were obtained for D_0_1A: CCE-Curc (L-Lat-Ph)).^71,78^ Interestingly, the spin density associated with D_0_1A: CCE-Curc (R-Lat-Ph) is much more delocalized compared to D_0_1A: CCE-Curc (C-En), with relatively high QTAIM populations not only on the phenolic radicalization center but also on the aromatic ring, conjugated chain and keto–enolic group, although the highest population is still associated with the reaction site.

**Fig. 5:**
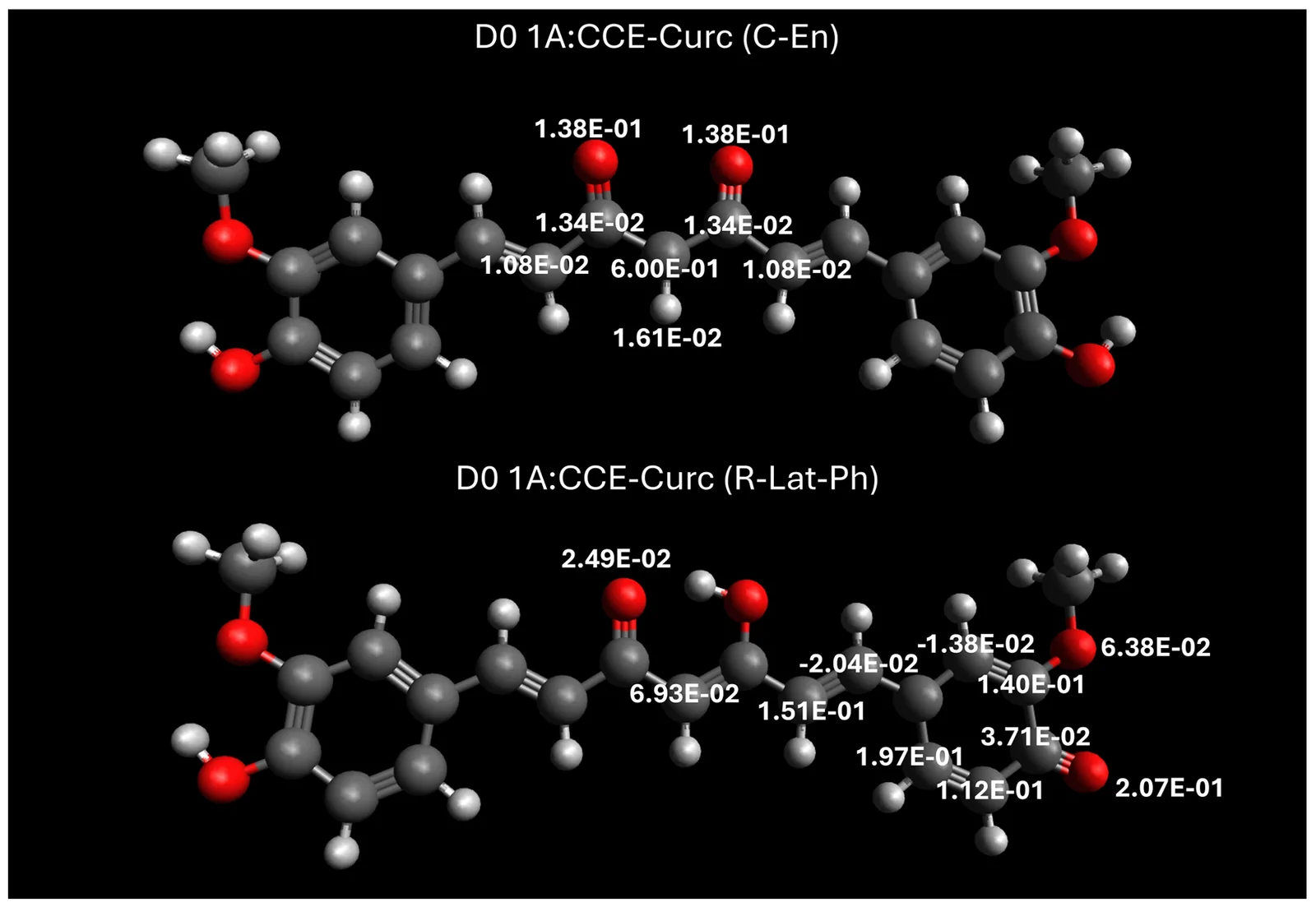
QTAIM electron spin density populations in e^−^ bohr^−3^ on selected atoms (spin density population > |10^−2^| e^−^ bohr^−3^) of both D_0_1A: CCE-Curc (C-En) and D_0_1A: CCE-Curc (R-Lat-Ph).

On the contrary, in case of D_0_1A: CCE-Curc (C-En) the highest spin density is associated with the carbon atom of the central methylene group rather than the enolic radicalization center. Besides, the bond lengths of both ketonic and enolic CO fragments significantly reduce compared with S_0_1A: CCE-Curc. In particular, they are both equal to 1.23 Å, *i.e.* the reference value for standard C=O double bonds.

To further investigate this point, we also simulated the reaction of D_0_1A: CCE-Curc with the radical TEMPO on the most spin populated sites of both C-En and R/L-Lat-Ph radicals, namely the enolic oxygen, the methylene carbon and both the phenolic oxygens. This analysis (Fig. 6) shows that the reaction is endoenergetic in all cases except for the radical attack of TEMPO on the carbon atom of methylene group. In other words, these data strongly support the view that, upon loss of the enolic hydrogen atom, the D_0_1A: CCE-Curc (C-En) is stabilized by a marked conjugation of the unpaired electron, which is delocalized on the two carbonyl groups and the methylene carbon, albeit with a strong preference toward the latter.

**Fig. 6:**
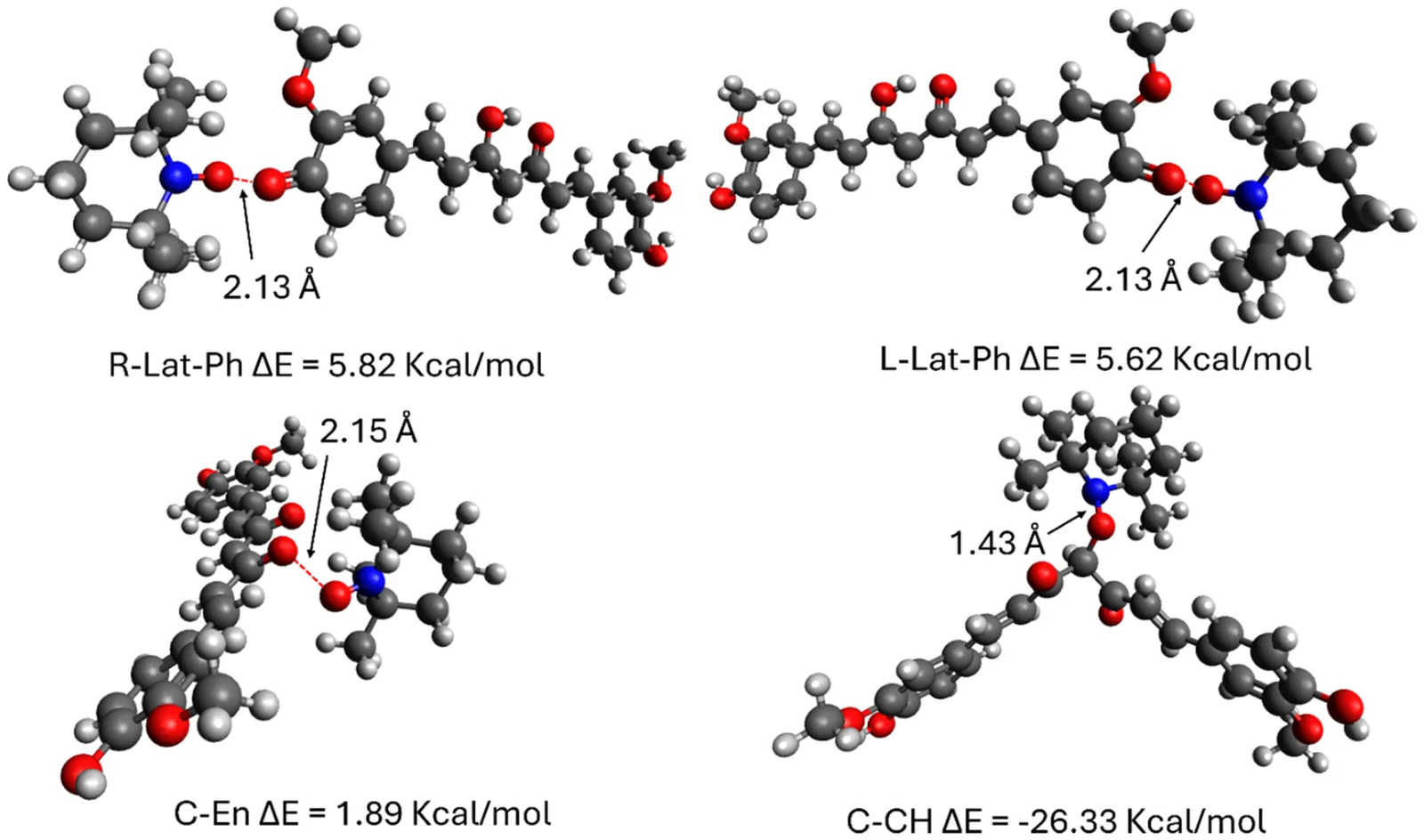
Products of reactions between radical TEMPO and curcuminoids radicals and corresponding reaction energies in kcal mol^−1^.

#### 3.1.4. Distinct roles of phenolic and enolic hydroxyl groups in curcuminoid radicalization

The relative energetics of phenolic and enolic O–H bonds in the ground state (GS) S_0_ is principally determined by the intramolecular hydrogen bond within the keto–enol group. When this interaction is present, enolic O–H bond dissociation energies (BDEs) are on average approximately 20 kcal mol^−1^ higher than the corresponding phenolic ones, suggesting that phenolic groups are more favorable sites for hydrogen-atom donation in the GS. However, when this H-bond cannot form, as in compounds 4A: OCE-Curc and 5A: T-E-Curc (Fig. 2), the BDEs of the two groups become comparable, being both around 80 kcal mol^−1^. This indicates that the difference in BDEs does not primarily arise from the distinct chemical nature of phenolic and enolic hydroxyl groups, but rather from the exceptional stabilization of the keto–enol moiety associated with the resonance-assisted hydrogen bond (RAHB) (see Section S1 of the SI for further details).^83^ Upon excitation to the S_1_ state, the BDEs of both phenolic and enolic O–H bonds decrease substantially, by approximately 30 and 50 kcal mol^−1^, respectively, making the two groups energetically comparable with respect to the hydrogen atom donation (Fig. 3). The orbital analysis suggests that intramolecular hydrogen bonding plays a less significant role in the excited-state electronic structure, thereby contributing to the reduced distinction between phenolic and enolic radicalization (Fig. 4). Moreover, the limited structural changes upon excitation, including a shortening of the O–H bonds by only about 0.01 Å, suggest that the decrease in BDEs primarily reflects the enhanced stabilization of the resulting radicals in the excited state D_1_, rather than a substantial destabilization of the reagents in S_1_.

The spin-density distributions further distinguish the two radicalization pathways: phenolic H-atom loss leads to a radical with spin delocalized across the aromatic ring and conjugated framework, whereas enolic H-atom loss yields a species with spin density concentrated predominantly on the central methylene carbon (Fig. 4). Consistently, the TEMPO trapping calculations identify this carbon as the only investigated site for which the reaction is exoenergetic, supporting the stabilization of the enolic radical through conjugation of the dicarbonyl moiety. Nevertheless, despite the more favorable radicalization energetics in the excited state, the O–H BDEs remain relatively high. Thus, while these results identify the factors governing radicalization propensity and the stabilization of the resulting species, they do not, on their own, fully account for the observed radical formation. This motivates the exploration of an alternative mechanism involving low-lying triplet states and bimolecular hydrogen-atom transfer (HAT), which may also be relevant to the antioxidant activity of curcuminoids (see Section 3.3).

### 3.2. Experimental results

In the sought of conclusive experimental evidence that curcumin radicalization can be elicited through photoexcitation, selected experiments were performed using the permanent radical TEMPO as a spin trap able to form stable adducts with putative curcumin radicals. Namely, we exploited LC-HRMS to detect species with the mass of these adducts and we looked for their fingerprints in UV-visible absorption spectra.

#### 3.2.1. LC-HRMS

To validate whether the hypothesis of some kind of radical HAT mechanism may be at play following curcumin excitation, several control experiments were carried out under argon inert atmosphere to avoid potential degradation of curcumin caused by radical oxygen species. The solvent of choice for all the experiments was acetonitrile, since the best singlet oxygen generation performances were noted from previous experiments in this solvent. All samples were exposed to UV light for 10 min using a commercially available Wood lamp.

Effective detection of interesting species was obtained using an UHPLC-HRMS instrument equipped with an Orbitrap. A 10 mg L^−1^ solution of curcumin with TEMPO in a 1 : 3 (m/m ratio) in degassed acetonitrile was stirred under dark for 10 min and injected; the same solution was then irradiated using the Wood lamp for 10 min and injected. Since the mass spectrometric detection was performed with a high-resolution mass spectrometer, the identification of compounds (curcumin and curcumin–TEMPO adduct) was based on their exact masses (see Table 1 in the Materials and methods section).

We found the presence of both curcumin (retention time = 12.48 min, exact mass = 367.11871) and curcumin–TEMPO adduct (retention time = 15.13 min, exact mass = 522.24973) in the irradiated solution. No trace of a similar species was found in the non-irradiated solution, instead (Fig. 7).

**Fig. 7:**
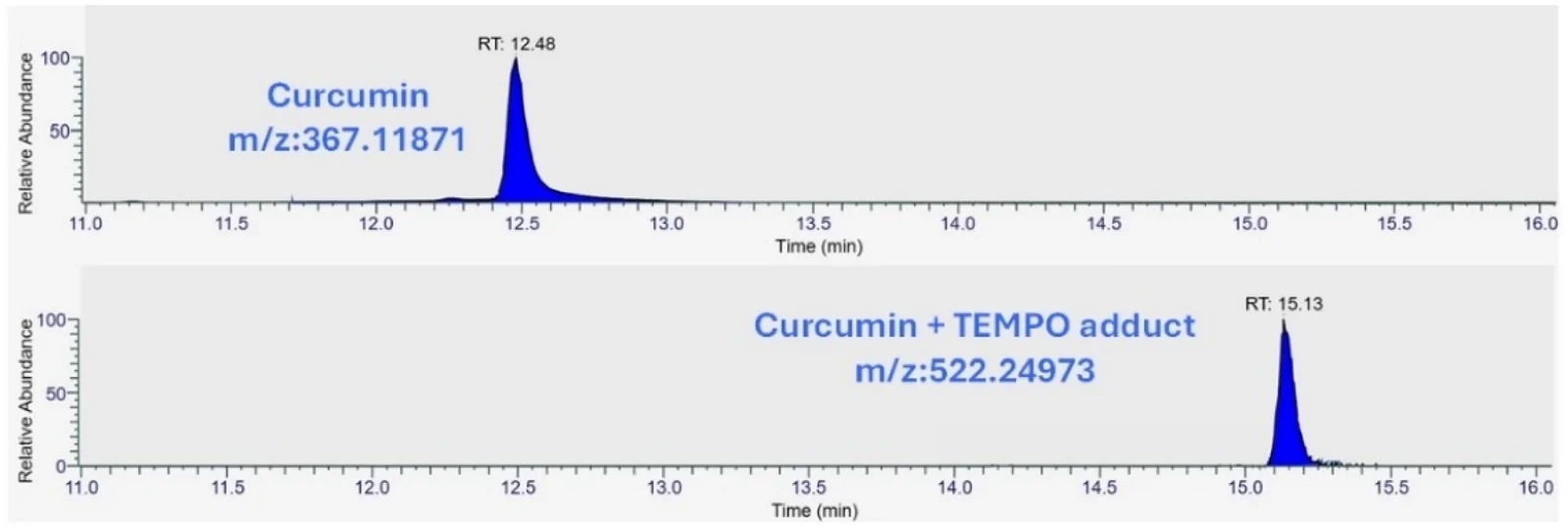
Negative ion Extracted Ion Chromatograms (XIC) of curcumin and curcumin–TEMPO adduct (stylized as curcumin + TEMPO adduct) in the solution irradiated using the Wood lamp. XIC chromatograms were extracted with a 5 ppm window.

Albeit not detailing the possible structure of the adduct produced, the mass signature for the latter brings strong support to the idea that some form of HAT mechanism should be at play, the trapping of the TEMPO scavenger being due to the formation of an unpaired spin center on curcumin following irradiation, related to the loss of a hydrogen atom.

#### 3.2.2. UV-vis absorption spectroscopy

In order to evidence the formation of radical curcuminoid species upon photosensitization, we submitted curcumin solutions to UV-vis illumination for several time lapses in the presence of overwhelming quantities (100 : 1) of the permanent radical TEMPO, *i.e.*, in an experimental condition in which any curcumin radicals putatively produced upon light irradiation should be readily stabilized by formation of permanents curcuminoid–TEMPO covalent adducts. We sought evidence of the presence of such adducts by means of UV-vis absorption spectroscopy. Considering that curcumin was reported to be highly photolabile in certain environments, we worked in acetonitrile, a solvent in which the compound is rather stable, and compared the spectra obtained for the curcumin/TEMPO mixture to those of baseline samples consisting of pure curcumin and pure TEMPO at the same concentration as in the mixture (see Materials and methods for details). These triplets of samples were irradiated simultaneously for different times under a Wood lamp (see Materials and methods for details). As shown in Fig. 8a, the TEMPO sample exhibits a main absorption band in the UVB, peaked at ≈240 nm, and negligibly low absorption over the curcumin absorption spectral band (350–650 nm). Moreover, the absorption spectrum is minimally affected by irradiation. The pure curcumin sample exhibited the typical band peaked around 420 nm characteristic of the enol tautomers, see Fig. 8b. Conversely, in agreement with wide literature evidence, the shoulder at ≈350 nm which was revealed in aqueous environment and attributed to the presence of sizeable amounts of the *cis*-diketo tautomer in previous reports^84,85^ was not resolved. Indeed, this structure is expected to be adopted in water, but not in organic solvents, due to its superior polarity with respect to the enol conformers, making it more prone to react with water molecules according to the peculiar dual proton-donating and proton-accepting nature of this solvent. A more extensive discussion on this point can be found, *e.g.*, in ref. 68 and 70. The absence of spectroscopic fingerprints of the diketo tautomer stems in support of the results of our *in silico* calculations, yielding energies lower by several kcal mol^−1^ for the closed enol then for the diketo tautomers both in the gas phase and in solution (with implicit and explicit solvent treatment). The enol band is gradually attenuated upon irradiation. Appearance of a doublet of faint absorption bands peaked at ≈289 nm and ≈313 nm corresponds to the reduction of the enol peak intensity, as shown in Fig. 8b. The generation of the species absorbing at 289 nm is photosensitized at the same rate as the photodecomposition of curcumin, (1.1 ± 0.1 × 10^−3^ s^−1^, see Fig. 8b inset). Accordingly, we interpret the appearance of the doublet as due to the formation of curcumin photodegradation products (eventually at the steady state vanillin and ferulic acid;^50,86^ notably, vanillin absorbs at 281 nm.) in aqueous environment,^87^ while the main absorption peak of ferulic acid falls at 316 nm in ethyl acetate.^88^ A substantially different behavior is shown by the curcumin/TEMPO mixture, as shown in Fig. 8c. The enol band intensity drops consistently already upon illumination for 2 minutes. The appearance of an intense band peaked at 283 nm corresponds to the enol band fading. The intensities of the 283 nm and the enol bands are anticorrelated and light-dose dependent, see Fig. 8c inset.

**Fig. 8:**
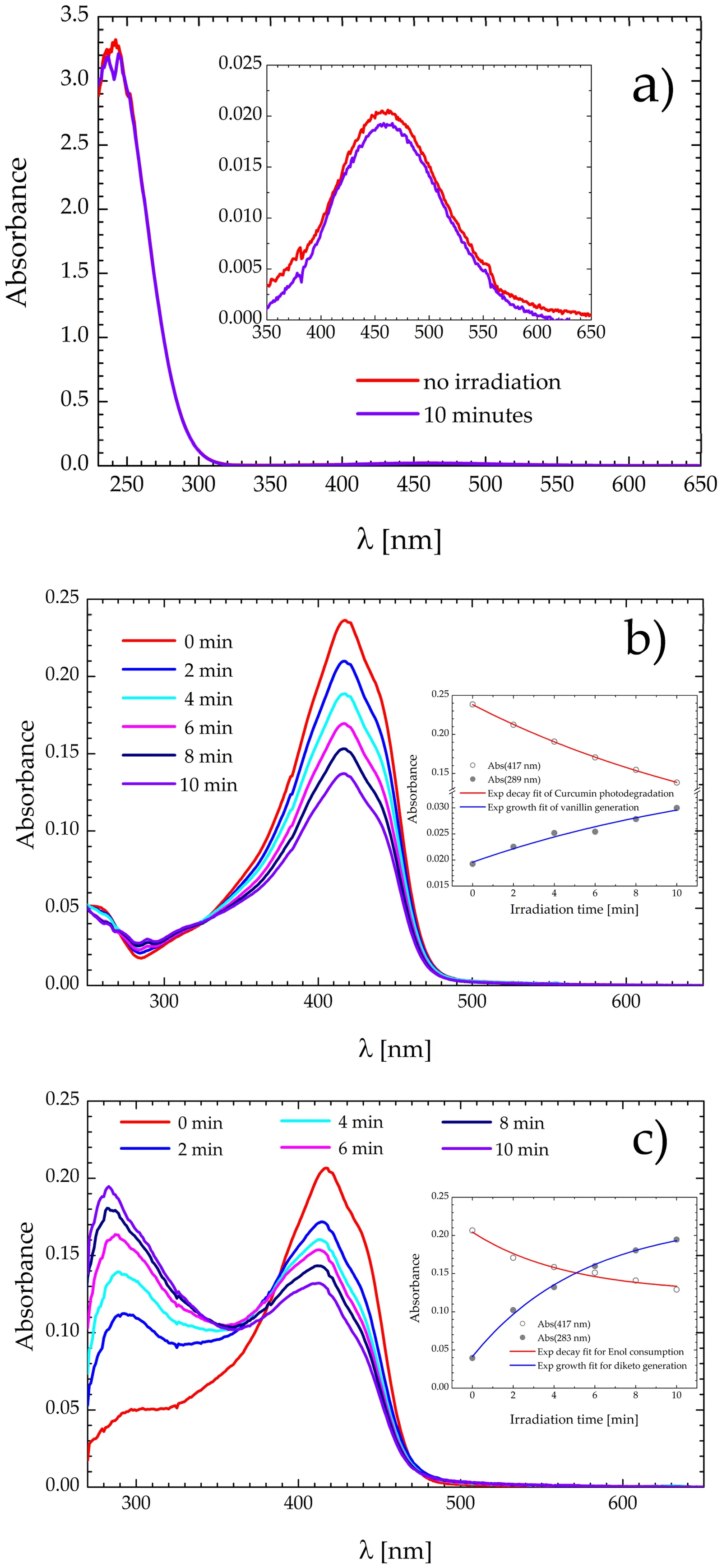
(a) UV-vis absorption spectra, at different times of irradiation by a Wood lamp, of a solution of TEMPO (in the inset zoom on the curcumin absorption band). (b) Same plot for a degassed (*i.e.*, Ar saturated) solution of curcumin; in the inset, plots of the absorbance at the enol-curcumin absorption peak and at 289 nm (attributed to vanillin) as a function of irradiation time, together with exponential fits. (c) Same plots as in panel (b) for a de-oxygenated mixture of 100 : 1 TEMPO : curcumin mixture.

Taking advantage of the calculations on curcuminoid radical–TEMPO adducts formation, we may gain some insight into this behavior. In fact, the 283 nm band falls in the spectral region characteristic of the diketo tautomers of curcumin. In previous works we showed that substitutions at the vinylic proton have the effect of shifting considerably the keto–enolic equilibrium towards the diketo conformers.^68,70^ As our present calculations suggest that the radical spin density tends to concentrate in the neighborhoods of such proton, we interpret the appearance of the band located in the same spectral region of the diketo species as due to the TEMPO radical covalent binding to the vinyl moiety, which in turn induces perturbation of the RAHB semi-aromatic ring and substantial destabilization of the enol conformers.

As apparent from Fig. 8c, the molar extinction coefficients of the photolabile enol species and of the formed adduct are similar, in good agreement with previous results suggesting such similarity to hold between enol and diketo curcuminoids.^68,70^ This interpretation is further supported by literature data showing that the formation of adducts with radicals other than TEMPO occurs preferentially at the vinyl and induces breaking of the overall planarity of the curcumin molecule.^50^ Interestingly, in the same article it is shown that formation of the curcumin carbonyl radical is prodromic to photodecomposition of curcumin and production of both vanillin and ferulic acid. Formation of stable, non-radical adducts with the permanent radical TEMPO is expected to hinder such photolytic mechanisms. Accordingly, at variance with Fig. 8b, where, as discussed above, sizeable perturbation of the absorption spectral line-shape in the 280–330 nm wavelengths interval constitutes a fingerprint of these two photoproducts, we do not reveal any spectroscopic indication suggesting their presence in the UV-vis absorption spectra of Fig. 8c, acquired on samples in which the extreme excess of TEMPO with respect to curcuminoid radicals should assure prompt formation of the neutral adducts. Namely, according to the exponential fit shown in Fig. 8c the formation rate of the curcumin–TEMPO adduct is (3.1 ± 0.4) × 10^−3^ s^−1^, very similar to the consumption rate of enol curcumin, (4.1 ± 1.3) × 10^−3^ s^−1^.

#### 3.2.3. Singlet oxygen generation efficiency

It is well assessed that ^1^O_2_ generation is not the main mechanism of curcuminoids phototoxicity.^39,51,58,59^ However, in the assumption that photosensitized activity is initiated by curcuminoid radical generation upon illumination of ground state curcumin, the ^1^O_2_ production should scale as the concentration of curcuminoid radical. The latter is in turn proportional to the product between curcuminoid radical formation probability and its lifetime. In order to estimate the proneness of different curcuminoids to undergo radicalization, their ^1^O_2_ production efficiency was assessed in both polar non-H-bonding (acetonitrile) and protic solvent (methanol) using the 9,10-dimethylanthracene fluorescence assay. In agreement with previously published results, the ^1^O_2_ production is maximally efficient for dimethoxycurcumin in acetonitrile.^67^ Accordingly, the singlet oxygen produced for this curcuminoid in this solvent was used as a benchmark and arbitrarily set to unit value. The amounts of ^1^O_2_ produced by curcumin, bisdemethoxycurcumin, and cyclovalone in acetonitrile, as well as those produced by the four curcuminoids in methanol, are reported in Table 2 as fractional concentrations relative to the amount of ^1^O_2_ produced by dimethoxycurcumin in acetonitrile upon irradiation with the same Wood lamp for the same time (30 s). Over this photo-sensibilization time scale, the fluorescence emission spectra of samples of the same curcuminoids in the absence of 9,10-dimethylanthracene decreased by less than 10% at worst. Accordingly, photodegradation of the curcuminoids was neglected. Similarly, irradiating samples of deoxygenated 9,10-dimethylanthracene for the same time did not induce any appreciable change in their fluorescence emission spectrum in any of the two solvents.

**Table 2:** ^1^O_2_ quantum yields of the curcuminoids in different solvents. The values are expressed as fractions of the ^1^O_2_ quantum yield of dimethoxycurcumin in acetonitrile. The average fluorescence lifetimes of the curcuminoids are also reported in parentheses

Curcuminoid	*Φ*_acetonitrile_ (*τ*_av_)	*Φ*_methanol_ (*τ*_av_)	*Φ*_DMSO_ (*τ*_av_)
Curcumin	0.31 (695 ps)	0.07 (162 ps)	<0.01 (155 ps)
Dimethoxycurcumin	1 (862 ps)	0.09 (418 ps)	
Bisdemethoxycurcumin	0.36 (321 ps)	0.13 (720 ps)	
Cyclovalone	0.55 (394 ps)	0.05 (123 ps)	

In the non-H-bonding solvent acetonitrile the singlet oxygen production of curcumin is comparable but somewhat lower than that of cyclovalone, in agreement with the lower value obtained for the pertaining calculated BDEs in the S_1_ state. However, the situation is reverted in methanol. More in general, the ^1^O_2_ generation efficiency is systematically and notably depressed in the latter solvent. In the case of curcumin, we also estimated the yield in dimethylsulfoxide, which is even lower than in methanol, and barely detectable (<0.01). These findings indicate that the environmental properties, and particularly the ability of establishing solute–solvent H-bonding patterns, are capable of notably affecting either the curcuminoids proneness to undergo radicalization or the radicals' lifetime. For instance, quenching of the radical species by solute–solvent charge transfer reactions may play a role in reducing the overall radicals' lifetime, thus their probability to interact with oxygen. Notably, similar mechanisms were previously postulated to have a prominent role also in the S_1_ state deactivation for curcumin^65^ and cyclovalone^69^ in H-bonding solvents, while they were seemingly inactive for bisdemethoxycurcumin.^66^ Accordingly, the reduction in photosensitizing potential in changing from non-H-bonding to H-bonding environment is more prominent for the former than for the latter curcuminoid.

Finally, it must be considered that, since curcuminoids radicalization is a negligibly rare event in the ground state in our experimental conditions, *i.e.* in the absence of oxidizing species, another important factor dictating the actual probability to undergo radicalization is the S_1_ state lifetime of the different curcuminoids, which sets an upper limit to the time scale on which the radicalization reaction must occur. The examined curcuminoids exhibit rather different average fluorescence lifetimes in the different solvents (see Table 2). The complex interplay between the above and other excited-state dynamics of the curcuminoids under study jeopardizes any attempt at rationalizing their phototoxic potential in terms of theoretical calculations of the BDEs in the gas phase. More in general, at the light of the present preliminary study, the proneness of a given curcuminoid to undergo radicalization in the excited state appears to be only one of the several aspects influencing the ability to photosensitize the production of singlet oxygen.

### 3.3. Further exploration of the mechanism generating curcuminoid radicals

We have established that curcuminoids undergo radicalization upon irradiation with UV-vis light (see Fig. 7 and 8), the radical produced either decomposing or being trapped in presence of TEMPO. According to our calculations and the time dependence of the absorption bands shown in Fig. 8, it appears highly likely for the radical to involve the central carbon in a to the carbonyl groups. The elevated BDE's estimated for the chosen species suggest, however, that a mechanism non-involving the direct and monomolecular H atom-loss following excitation should be sought. In this respect, it is useful to recall that carbonylic compounds undergo intersystem crossing with high quantum yield, producing long-lived T_1_ triplet states (see the paper by Losantos *et al.* for the specific case of curcumin^89^). Indeed, the di-radical nature of the latter would pave the way toward the bimolecular production of curcumin-based radicals *via* HAT between a curcumin molecule in the T_1_ state and one in the ground state, provided that the energy barrier for the latter process was sufficiently low.

To verify such possibility we, therefore, sought possible HAT pathways leading to the formation of species that may help to rationalize the discussed experimental outcomes (Fig. 9). We were, *de facto*, successful in locating a low-lying transition state for the HAT shifting a hydrogen atom from the enolic hydroxyl of a S_0_ CCE-Curc to the central carbon atom of the T_1_ CCE-Curc, the latter being among the molecular fragments that appear to be receiving the electron involved in the excitation (see Fig. 4 for the difference between HOMO and LUMO electron densities, and Fig. 9 for the energetics of optimized reactants, products, and transition state). As net result for the mentioned process, the HAT between a T_1_ CCE-Curc and a S_0_ CCE-Curc generates a D_0_1A: CCE-Curc (C-En) species (see Fig. 6) plus a partial adduct presenting the central carbon atom of the α,β-unsaturated β-diketone group in the enolic form bearing an additional hydrogen atom. This product has the carbon atom bonded to the enolic hydroxyl also bearing an unpaired electron. As shown by the energy results, the overall process is endothermic despite the excited nature of the reacting CCE-Curc in its lowest triplet state. This finding, together with the low energy barriers, clearly indicates that the HAT should be reversible, leading back to the ground state CCE-Curc molecules.

**Fig. 9:**
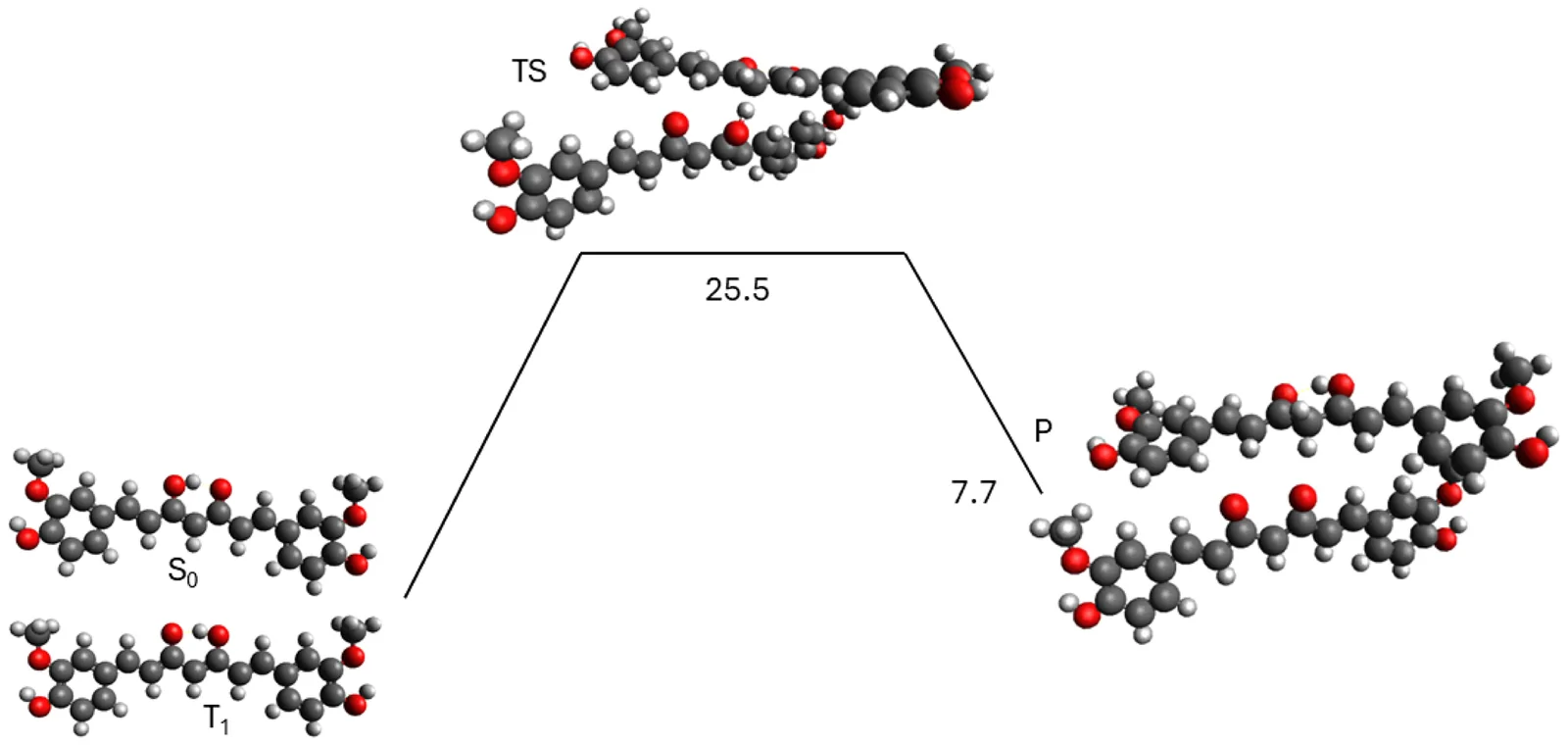
Zero-point energy corrected reaction profile (in kcal mol^−1^) for the HAT from the enolic hydroxyl of a S_0_ curcumin and the T_1_ state of another one.

If the products of the HAT process survived sufficiently long, the D_0_1A: CCE-Curc (C-En) species may be scavenged leading to the adduct C–CH in Fig. 6 in presence of TEMPO, so to justify the disappearance of the latter seen in Fig. 8c. The second produced compounds may, also, lead to a TEMPO adduct, albeit probably less stable than the one formed by D_0_1A: CCE-Curc (C-En) due to a reduced conjugation in the π systems; it could, as well, initiate a cascade of radical reactions among which 1,2 H-shifts to more stable species, and potentially initiating a polymerization by adding to the carbon–carbon double bonds of the curcuminoids. In all cases, the possibility of extending the molecular conjugation *via* the formation of a keto–enolic moiety would be impaired, thus rationalizing the progressive disappearance of the curcumin absorption band.

In the end, the mechanism just discussed seems to provide a reasonable explanation for the radical-based origin of the curcumin cytotoxicity hypothesized in the Introduction, as well as for the evidence that only a limited amount of ^1^O_2_ is generated upon irradiation of curcumin. In fact, the low barrier present for the HAT process following excitation and intersystem crossing permits a substantial production of radicals made relatively stable by the conjugation with double bond present, while de-pauperizing the environment of the T_1_ CCE-Curc species needed to transfer energy to ^3^O_2_. Most importantly, in view of such mechanism it may be possible to reconcile the complex picture provided by the juxtaposition of previous studies with the results discussed in this work with the goal of managing computer-based predictions on the efficacy of curcuminoids as phototoxic agents. This might be achieved considering, as a general rule, the intermolecular H-bonding patterns of tentative curcumin-based phototoxic drug compounds with their substrate, as this could enhance radicalization of the latter *via* HAT, as well as the impact of phenolic substitutions compared to curcumin itself on the barrier height for HAT itself. Work along these lines is already underway.

## 4 Conclusion

In this article we have presented a thorough computational analysis of the proneness of a panel of curcumin analogues to undergo radicalization through proton ablation in both the ground and first excited singlet state, with the aim of shedding light onto the molecular mechanisms underlying the antioxidant and phototoxic properties of this class of phytochemicals. In the ground state, the bond dissociation energy for phenyl proton dissociation is in the range of 80 kcal mol^−1^, suggesting that curcuminoids may act as efficient quenchers for many oxidizing species produced in metabolic pathways. In the first excited singlet state the proneness to proton abstraction is notably increased and the differential tendency to dissociate of the phenolic compared to enolic proton reduced. The BDEs seem to be significantly lower than the excitation energy, indicating that formation of curcuminoid radicals can be an efficient excited-state deactivation pathway. In both ground and excited states, in the most stable radical species the radical charge seems to concentrate around the vinylic carbon.

For the parent compound, curcumin, experiments relying on spin trapping by the permanent radical TEMPO were undertaken to demonstrate photosensitized production of excitonic curcuminoid radicals. HPLC-mass spectroscopy has allowed to detect curcumin–TEMPO covalent adducts in solutions submitted to UV-vis irradiation. UV-vis absorption spectroscopy results suggest that these adducts may involve the curcumin vinyl carbon, in agreement with the *in silico* results.

A bimolecular photosensitized radicalization process was eventually proposed, involving CCE-Curc S_0_ → T_1_ intersystem crossing, followed by abstraction of the hydrogen atom from the enolic hydroxyl of a ground state CCE-Curc. This process was found to involve surmounting a barrier of 25.5 kcal mol^−1^, much lower than for any of the other processes leading to an H-atom loss investigated herein. Importantly, the two radicals formed through this excited-state deactivation pathway can couple to generate a hemiketal, which provides a convincing mechanistic explanation of the cytotoxic activity of photoexcited curcumin.

## Author contributions

G. B. investigation, formal analysis, validation, visualization, writing – original draft, writing – revision and editing. M. V. R. investigation, formal analysis, validation. F. V. investigation, visualization, writing – revision and editing. A. M. methodology, investigation, formal analysis, validation, writing – original draft, writing – revision and editing, supervision, resources. E. T. methodology, investigation, formal analysis, validation, writing – revision and editing. D. M. investigation, writing – revision and editing. A. P. methodology, investigation, validation, writing – revision and editing, supervision, resources. M. M. conceptualization, methodology, investigation, formal analysis, validation, visualization, writing – original draft, writing – revision and editing, supervision, resources. L. N. conceptualization, methodology, investigation, formal analysis, validation, visualization, writing – original draft, writing – revision and editing, supervision, resources.

## Conflicts of interest

There are no conflicts to declare.

## Supplementary Material

RA-OLF-D6RA07998A-s001

## Data Availability

The data supporting this article have been included as part of the supplementary information (SI). Supplementary information: (S0) optimized geometries for the compounds studied. (S1) conformational analysis of curcumin. (S2) effects of semi-empirical corrections for the dispersion interactions. See DOI: https://doi.org/10.1039/d6ra07998a.
