# Supplementary material for: Role of radicalization in the antioxidant and phototoxic properties of curcuminoids
Source: RSC Adv. 2026 Nov 6. Online ahead of print. doi: 10.1039/d6ra07998a (PMC13741483; doi:10.1039/d6ra07998a)
Supplement: Supplementary file 1 [file RA-OLF-D6RA07998A-s001.pdf]

Supplementary Information (SI) for RSC Advances.
This journal is © The Royal Society of Chemistry

Supporting Information file for:

Role of radicalization in the antioxidant and phototoxic properties of curcuminoids

Giovanna Bruno, Maria Vittoria Rossi, Federico Vavassori, Angelo Maspero, Elisa Terzaghi, Damiano Monticelli, Andrea Penoni, Massimo Mella* and Luca Nardo*

S0. Optimized geometries for the compounds studied

Curcuminoids

1A: CCE-Curc S0

```
47 48  0  0  0  0  0  0  0  0999 V2000
  -8.4773    2.2854    0.0001 C   0  0  0  0  0  0  0  0  0  0  0  0
  -8.5754    0.8658    0.0001 O   0  0  0  0  0  0  0  0  0  0  0  0
  -7.4167    0.1301    0.0000 C   0  0  0  0  0  0  0  0  0  0  0  0
  -7.6335   -1.2616    0.0000 C   0  0  0  0  0  0  0  0  0  0  0  0
  -6.5418   -2.1237   -0.0000 C   0  0  0  0  0  0  0  0  0  0  0  0
  -5.2487   -1.6172   -0.0000 C   0  0  0  0  0  0  0  0  0  0  0  0
  -5.0125   -0.2342   -0.0000 C   0  0  0  0  0  0  0  0  0  0  0  0
  -6.1264    0.6326    0.0000 C   0  0  0  0  0  0  0  0  0  0  0  0
  -5.9539    1.7012    0.0000 H   0  0  0  0  0  0  0  0  0  0  0  0
  -3.6816    0.3568   -0.0000 C   0  0  0  0  0  0  0  0  0  0  0  0
  -2.4882   -0.2666   -0.0001 C   0  0  0  0  0  0  0  0  0  0  0  0
  -1.2314    0.5002   -0.0001 C   0  0  0  0  0  0  0  0  0  0  0  0
   0.0071   -0.2330   -0.0001 C   0  0  0  0  0  0  0  0  0  0  0  0
   1.2188    0.4306   -0.0001 C   0  0  0  0  0  0  0  0  0  0  0  0
   2.4851   -0.2760   -0.0001 C   0  0  0  0  0  0  0  0  0  0  0  0
   3.6749    0.3596   -0.0000 C   0  0  0  0  0  0  0  0  0  0  0  0
   5.0049   -0.2332    0.0000 C   0  0  0  0  0  0  0  0  0  0  0  0
   5.2405   -1.6164    0.0000 C   0  0  0  0  0  0  0  0  0  0  0  0
   6.5332   -2.1238    0.0001 C   0  0  0  0  0  0  0  0  0  0  0  0
   7.6258   -1.2631    0.0001 C   0  0  0  0  0  0  0  0  0  0  0  0
   7.4099    0.1288    0.0000 C   0  0  0  0  0  0  0  0  0  0  0  0
   6.1200    0.6325    0.0000 C   0  0  0  0  0  0  0  0  0  0  0  0
   5.9497    1.7015   -0.0000 H   0  0  0  0  0  0  0  0  0  0  0  0
   8.5689    0.8635    0.0000 O   0  0  0  0  0  0  0  0  0  0  0  0
   8.4727    2.2833   -0.0000 C   0  0  0  0  0  0  0  0  0  0  0  0
   9.4962    2.6536   -0.0000 H   0  0  0  0  0  0  0  0  0  0  0  0
   7.9546    2.6438   -0.8946 H   0  0  0  0  0  0  0  0  0  0  0  0
   7.9546    2.6438    0.8946 H   0  0  0  0  0  0  0  0  0  0  0  0
   8.8875   -1.7594    0.0001 O   0  0  0  0  0  0  0  0  0  0  0  0
   9.4931   -1.0052    0.0001 H   0  0  0  0  0  0  0  0  0  0  0  0
   6.7193   -3.1909    0.0001 H   0  0  0  0  0  0  0  0  0  0  0  0
   4.4083   -2.3092    0.0001 H   0  0  0  0  0  0  0  0  0  0  0  0
   3.6429    1.4454    0.0000 H   0  0  0  0  0  0  0  0  0  0  0  0
   2.4174   -1.3578   -0.0001 H   0  0  0  0  0  0  0  0  0  0  0  0
   1.2732    1.7583   -0.0000 O   0  0  0  0  0  0  0  0  0  0  0  0
```

0.3108 2.0636 -0.0000 H 0 0 0 0 0 0 0 0 0 0 0 0
-0.0031 -1.3142 -0.0001 H 0 0 0 0 0 0 0 0 0 0 0 0
-1.2446 1.7580 -0.0000 O 0 0 0 0 0 0 0 0 0 0 0 0
-2.4045 -1.3481 -0.0001 H 0 0 0 0 0 0 0 0 0 0 0 0
-3.6407 1.4437 -0.0000 H 0 0 0 0 0 0 0 0 0 0 0 0
-4.4158 -2.3091 -0.0001 H 0 0 0 0 0 0 0 0 0 0 0 0
-6.7292 -3.1905 -0.0000 H 0 0 0 0 0 0 0 0 0 0 0 0
-8.8958 -1.7567 0.0000 O 0 0 0 0 0 0 0 0 0 0 0 0
-9.5003 -1.0016 0.0001 H 0 0 0 0 0 0 0 0 0 0 0 0
-7.9586 2.6453 -0.8944 H 0 0 0 0 0 0 0 0 0 0 0 0
-9.5004 2.6571 0.0001 H 0 0 0 0 0 0 0 0 0 0 0 0
-7.9586 2.6452 0.8946 H 0 0 0 0 0 0 0 0 0 0 0 0
1 46 1 0 0 0 0
1 47 1 0 0 0 0
2 1 1 0 0 0 0
3 2 1 0 0 0 0
4 3 2 0 0 0 0
4 43 1 0 0 0 0
5 4 1 0 0 0 0
6 5 2 0 0 0 0
6 7 1 0 0 0 0
7 8 2 0 0 0 0
8 3 1 0 0 0 0
8 9 1 0 0 0 0
10 7 1 0 0 0 0
10 40 1 0 0 0 0
11 10 2 0 0 0 0
12 11 1 0 0 0 0
12 38 2 0 0 0 0
13 12 1 0 0 0 0
13 14 2 0 0 0 0
14 15 1 0 0 0 0
14 35 1 0 0 0 0
15 16 2 0 0 0 0
16 33 1 0 0 0 0
16 17 1 0 0 0 0
17 18 1 0 0 0 0
18 32 1 0 0 0 0
18 19 2 0 0 0 0
19 31 1 0 0 0 0
20 19 1 0 0 0 0
20 29 1 0 0 0 0
21 24 1 0 0 0 0
21 20 2 0 0 0 0
22 17 2 0 0 0 0
22 21 1 0 0 0 0
23 22 1 0 0 0 0
25 26 1 0 0 0 0
25 24 1 0 0 0 0
25 28 1 0 0 0 0

27 25 1 0 0 0 0
30 29 1 0 0 0 0
34 15 1 0 0 0 0
35 36 1 0 0 0 0
37 13 1 0 0 0 0
39 11 1 0 0 0 0
41 6 1 0 0 0 0
42 5 1 0 0 0 0
43 44 1 0 0 0 0
45 1 1 0 0 0 0
M END

3A:T-DIK-Curc S0

47 48 0 0 0 0 0 0 0 0999 V2000
-0.0025 3.2765 -0.2923 C 0 0 0 0 0 0 0 0 0 0 0 0
-0.9756 2.3132 -0.9800 C 0 0 0 0 0 0 0 0 0 0 0 0
-2.0359 1.7492 -0.1217 C 0 0 0 0 0 0 0 0 0 0 0 0
-2.9698 0.9268 -0.6386 C 0 0 0 0 0 0 0 0 0 0 0 0
-4.0816 0.2793 0.0395 C 0 0 0 0 0 0 0 0 0 0 0 0
-4.3325 0.4115 1.4138 C 0 0 0 0 0 0 0 0 0 0 0 0
-5.4091 -0.2315 2.0090 C 0 0 0 0 0 0 0 0 0 0 0 0
-6.2629 -1.0227 1.2468 C 0 0 0 0 0 0 0 0 0 0 0 0
-6.0265 -1.1681 -0.1347 C 0 0 0 0 0 0 0 0 0 0 0 0
-4.9520 -0.5264 -0.7261 C 0 0 0 0 0 0 0 0 0 0 0 0
-4.7643 -0.6340 -1.7867 H 0 0 0 0 0 0 0 0 0 0 0 0
-6.9407 -1.9736 -0.7654 O 0 0 0 0 0 0 0 0 0 0 0 0
-6.8051 -2.1862 -2.1662 C 0 0 0 0 0 0 0 0 0 0 0 0
-7.6266 -2.8408 -2.4514 H 0 0 0 0 0 0 0 0 0 0 0 0
-6.8816 -1.2432 -2.7172 H 0 0 0 0 0 0 0 0 0 0 0 0
-5.8528 -2.6727 -2.4014 H 0 0 0 0 0 0 0 0 0 0 0 0
-7.3137 -1.6493 1.8283 O 0 0 0 0 0 0 0 0 0 0 0 0
-7.7754 -2.1411 1.1349 H 0 0 0 0 0 0 0 0 0 0 0 0
-5.6078 -0.1343 3.0694 H 0 0 0 0 0 0 0 0 0 0 0 0
-3.6802 1.0200 2.0275 H 0 0 0 0 0 0 0 0 0 0 0 0
-2.8770 0.7249 -1.7040 H 0 0 0 0 0 0 0 0 0 0 0 0
-2.0217 2.0250 0.9269 H 0 0 0 0 0 0 0 0 0 0 0 0
-0.8412 2.0305 -2.1588 O 0 0 0 0 0 0 0 0 0 0 0 0
1.0072 2.4999 0.5626 C 0 0 0 0 0 0 0 0 0 0 0 0
2.0291 1.7402 -0.1840 C 0 0 0 0 0 0 0 0 0 0 0 0
2.9940 1.0653 0.4707 C 0 0 0 0 0 0 0 0 0 0 0 0
4.0743 0.2657 -0.0854 C 0 0 0 0 0 0 0 0 0 0 0 0
4.9858 -0.3389 0.8071 C 0 0 0 0 0 0 0 0 0 0 0 0
6.0309 -1.1137 0.3341 C 0 0 0 0 0 0 0 0 0 0 0 0
6.1952 -1.3086 -1.0517 C 0 0 0 0 0 0 0 0 0 0 0 0
5.3010 -0.7166 -1.9382 C 0 0 0 0 0 0 0 0 0 0 0 0
4.2540 0.0606 -1.4620 C 0 0 0 0 0 0 0 0 0 0 0 0
3.5688 0.5087 -2.1704 H 0 0 0 0 0 0 0 0 0 0 0 0
5.4445 -0.8799 -2.9993 H 0 0 0 0 0 0 0 0 0 0 0 0
7.2164 -2.0674 -1.5170 O 0 0 0 0 0 0 0 0 0 0 0 0

7.7135 -2.3827 -0.7493 H 0 0 0 0 0 0 0 0 0 0 0 0
6.9794 -1.7516 1.0932 O 0 0 0 0 0 0 0 0 0 0 0 0
6.9164 -1.6202 2.5089 C 0 0 0 0 0 0 0 0 0 0 0 0
5.9787 -2.0263 2.9019 H 0 0 0 0 0 0 0 0 0 0 0 0
7.7530 -2.1954 2.9013 H 0 0 0 0 0 0 0 0 0 0 0 0
7.0192 -0.5734 2.8129 H 0 0 0 0 0 0 0 0 0 0 0 0
4.8531 -0.1864 1.8705 H 0 0 0 0 0 0 0 0 0 0 0 0
2.9565 1.1294 1.5564 H 0 0 0 0 0 0 0 0 0 0 0 0
1.9589 1.7505 -1.2660 H 0 0 0 0 0 0 0 0 0 0 0 0
0.9319 2.5148 1.7793 O 0 0 0 0 0 0 0 0 0 0 0 0
0.5144 3.8337 -1.0757 H 0 0 0 0 0 0 0 0 0 0 0 0
-0.5361 3.9581 0.3717 H 0 0 0 0 0 0 0 0 0 0 0 0
1 47 1 0 0 0 0
1 24 1 0 0 0 0
2 1 1 0 0 0 0
2 3 1 0 0 0 0
3 22 1 0 0 0 0
4 3 2 0 0 0 0
4 5 1 0 0 0 0
5 6 2 0 0 0 0
6 7 1 0 0 0 0
6 20 1 0 0 0 0
7 19 1 0 0 0 0
8 17 1 0 0 0 0
8 7 2 0 0 0 0
9 8 1 0 0 0 0
10 9 2 0 0 0 0
10 5 1 0 0 0 0
11 10 1 0 0 0 0
12 9 1 0 0 0 0
13 12 1 0 0 0 0
14 13 1 0 0 0 0
15 13 1 0 0 0 0
16 13 1 0 0 0 0
18 17 1 0 0 0 0
21 4 1 0 0 0 0
23 2 2 0 0 0 0
24 45 2 0 0 0 0
25 26 2 0 0 0 0
25 24 1 0 0 0 0
26 43 1 0 0 0 0
27 26 1 0 0 0 0
27 28 2 0 0 0 0
28 42 1 0 0 0 0
29 28 1 0 0 0 0
29 37 1 0 0 0 0
30 29 2 0 0 0 0
31 32 2 0 0 0 0
31 30 1 0 0 0 0
32 27 1 0 0 0 0

33 32 1 0 0 0 0
34 31 1 0 0 0 0
35 30 1 0 0 0 0
35 36 1 0 0 0 0
37 38 1 0 0 0 0
38 41 1 0 0 0 0
38 40 1 0 0 0 0
38 39 1 0 0 0 0
44 25 1 0 0 0 0
46 1 1 0 0 0 0
M END

4A:OCE-Curc S0

47 48 0 0 0 0 0 0 0 0999 V2000
-8.6970 -1.9094 -0.0765 C 0 0 0 0 0 0 0 0 0 0 0 0
-8.6289 -0.4966 0.0759 O 0 0 0 0 0 0 0 0 0 0 0 0
-7.3919 0.0992 0.1049 C 0 0 0 0 0 0 0 0 0 0 0 0
-7.4445 1.4979 0.2575 C 0 0 0 0 0 0 0 0 0 0 0 0
-6.2597 2.2248 0.3018 C 0 0 0 0 0 0 0 0 0 0 0 0
-5.0352 1.5771 0.1976 C 0 0 0 0 0 0 0 0 0 0 0 0
-4.9623 0.1845 0.0460 C 0 0 0 0 0 0 0 0 0 0 0 0
-6.1697 -0.5443 0.0015 C 0 0 0 0 0 0 0 0 0 0 0 0
-6.1238 -1.6195 -0.1149 H 0 0 0 0 0 0 0 0 0 0 0 0
-3.7085 -0.5510 -0.0667 C 0 0 0 0 0 0 0 0 0 0 0 0
-2.4519 -0.0738 -0.0518 C 0 0 0 0 0 0 0 0 0 0 0 0
-1.2893 -0.9976 -0.1818 C 0 0 0 0 0 0 0 0 0 0 0 0
0.0219 -0.3340 -0.1682 C 0 0 0 0 0 0 0 0 0 0 0 0
1.2407 -0.9218 -0.3204 C 0 0 0 0 0 0 0 0 0 0 0 0
2.4569 -0.1182 -0.3078 C 0 0 0 0 0 0 0 0 0 0 0 0
3.7048 -0.6124 -0.1734 C 0 0 0 0 0 0 0 0 0 0 0 0
4.9613 0.1298 -0.1752 C 0 0 0 0 0 0 0 0 0 0 0 0
5.0563 1.4840 -0.5263 C 0 0 0 0 0 0 0 0 0 0 0 0
6.2768 2.1472 -0.5017 C 0 0 0 0 0 0 0 0 0 0 0 0
7.4351 1.4751 -0.1286 C 0 0 0 0 0 0 0 0 0 0 0 0
7.3605 0.1124 0.2204 C 0 0 0 0 0 0 0 0 0 0 0 0
6.1431 -0.5477 0.1934 C 0 0 0 0 0 0 0 0 0 0 0 0
6.0851 -1.5942 0.4654 H 0 0 0 0 0 0 0 0 0 0 0 0
8.5716 -0.4322 0.5646 O 0 0 0 0 0 0 0 0 0 0 0 0
8.6182 -1.8013 0.9492 C 0 0 0 0 0 0 0 0 0 0 0 0
9.6603 -2.0128 1.1812 H 0 0 0 0 0 0 0 0 0 0 0 0
8.0032 -1.9831 1.8366 H 0 0 0 0 0 0 0 0 0 0 0 0
8.2878 -2.4526 0.1333 H 0 0 0 0 0 0 0 0 0 0 0 0
8.6259 2.1229 -0.1074 O 0 0 0 0 0 0 0 0 0 0 0 0
9.2963 1.4848 0.1730 H 0 0 0 0 0 0 0 0 0 0 0 0
6.3544 3.1919 -0.7770 H 0 0 0 0 0 0 0 0 0 0 0 0
4.1733 2.0282 -0.8372 H 0 0 0 0 0 0 0 0 0 0 0 0
3.8227 -1.6828 -0.0080 H 0 0 0 0 0 0 0 0 0 0 0 0
2.3002 0.9513 -0.3865 H 0 0 0 0 0 0 0 0 0 0 0 0
1.3523 -2.2624 -0.4564 O 0 0 0 0 0 0 0 0 0 0 0 0
2.2271 -2.4662 -0.8052 H 0 0 0 0 0 0 0 0 0 0 0 0

0.0292 0.7406 -0.0314 H 0 0 0 0 0 0 0 0 0 0 0 0
-1.4416 -2.2087 -0.2981 O 0 0 0 0 0 0 0 0 0 0 0 0
-2.2461 0.9867 0.0535 H 0 0 0 0 0 0 0 0 0 0 0 0
-3.7925 -1.6304 -0.1772 H 0 0 0 0 0 0 0 0 0 0 0 0
-4.1269 2.1653 0.2358 H 0 0 0 0 0 0 0 0 0 0 0 0
-6.3204 3.3000 0.4193 H 0 0 0 0 0 0 0 0 0 0 0 0
-8.6409 2.1302 0.3603 O 0 0 0 0 0 0 0 0 0 0 0 0
-9.3277 1.4513 0.3093 H 0 0 0 0 0 0 0 0 0 0 0 0
-8.1996 -2.4212 0.7539 H 0 0 0 0 0 0 0 0 0 0 0 0
-9.7562 -2.1610 -0.0749 H 0 0 0 0 0 0 0 0 0 0 0 0
-8.2483 -2.2268 -1.0235 H 0 0 0 0 0 0 0 0 0 0 0 0
1 46 1 0 0 0 0
1 2 1 0 0 0 0
1 45 1 0 0 0 0
2 3 1 0 0 0 0
3 4 2 0 0 0 0
4 5 1 0 0 0 0
4 43 1 0 0 0 0
5 42 1 0 0 0 0
6 41 1 0 0 0 0
6 5 2 0 0 0 0
7 6 1 0 0 0 0
8 7 2 0 0 0 0
8 3 1 0 0 0 0
9 8 1 0 0 0 0
10 11 2 0 0 0 0
10 7 1 0 0 0 0
11 39 1 0 0 0 0
12 13 1 0 0 0 0
12 11 1 0 0 0 0
13 37 1 0 0 0 0
14 15 1 0 0 0 0
14 13 2 0 0 0 0
15 16 2 0 0 0 0
16 33 1 0 0 0 0
17 16 1 0 0 0 0
17 22 2 0 0 0 0
18 19 2 0 0 0 0
18 17 1 0 0 0 0
19 20 1 0 0 0 0
20 29 1 0 0 0 0
20 21 2 0 0 0 0
21 24 1 0 0 0 0
22 21 1 0 0 0 0
22 23 1 0 0 0 0
24 25 1 0 0 0 0
25 26 1 0 0 0 0
25 27 1 0 0 0 0
28 25 1 0 0 0 0
29 30 1 0 0 0 0

```
31 19 1 0 0 0 0
32 18 1 0 0 0 0
34 15 1 0 0 0 0
35 14 1 0 0 0 0
36 35 1 0 0 0 0
38 12 2 0 0 0 0
40 10 1 0 0 0 0
44 43 1 0 0 0 0
47 1 1 0 0 0 0
M END
```

5A:T-E-Curc S0

```
47 48 0 0 0 0 0 0 0 0999 V2000
  8.4372 -0.5324  1.3578 C 0 0 0 0 0 0 0 0 0 0 0 0
  8.0226  0.6190  0.6306 O 0 0 0 0 0 0 0 0 0 0 0 0
  6.7363  0.6654  0.1563 C 0 0 0 0 0 0 0 0 0 0 0 0
  6.4422  1.8421 -0.5606 C 0 0 0 0 0 0 0 0 0 0 0 0
  5.1720  2.0147 -1.1013 C 0 0 0 0 0 0 0 0 0 0 0 0
  4.1999  1.0374 -0.9330 C 0 0 0 0 0 0 0 0 0 0 0 0
  4.4726 -0.1383 -0.2180 C 0 0 0 0 0 0 0 0 0 0 0 0
  5.7656 -0.3078  0.3217 C 0 0 0 0 0 0 0 0 0 0 0 0
  5.9855 -1.2148  0.8701 H 0 0 0 0 0 0 0 0 0 0 0 0
  3.4958 -1.1974 -0.0050 C 0 0 0 0 0 0 0 0 0 0 0 0
  2.1915 -1.2184 -0.3416 C 0 0 0 0 0 0 0 0 0 0 0 0
  1.3649 -2.4342 -0.0832 C 0 0 0 0 0 0 0 0 0 0 0 0
 -0.0973 -2.3367 -0.1708 C 0 0 0 0 0 0 0 0 0 0 0 0
 -0.8941 -1.2464  0.0131 C 0 0 0 0 0 0 0 0 0 0 0 0
 -2.3358 -1.3278 -0.1220 C 0 0 0 0 0 0 0 0 0 0 0 0
 -3.1684 -0.2908  0.1012 C 0 0 0 0 0 0 0 0 0 0 0 0
 -4.6208 -0.2745 -0.0230 C 0 0 0 0 0 0 0 0 0 0 0 0
 -5.3807 -1.3824 -0.4270 C 0 0 0 0 0 0 0 0 0 0 0 0
 -6.7644 -1.3075 -0.5225 C 0 0 0 0 0 0 0 0 0 0 0 0
 -7.4273 -0.1236 -0.2180 C 0 0 0 0 0 0 0 0 0 0 0 0
 -6.6820  1.0002  0.1876 C 0 0 0 0 0 0 0 0 0 0 0 0
 -5.3024  0.9232  0.2824 C 0 0 0 0 0 0 0 0 0 0 0 0
 -4.7270  1.7855  0.5943 H 0 0 0 0 0 0 0 0 0 0 0 0
 -7.4539  2.1030  0.4571 O 0 0 0 0 0 0 0 0 0 0 0 0
 -6.8067  3.2988  0.8746 C 0 0 0 0 0 0 0 0 0 0 0 0
 -7.5977  4.0304  1.0294 H 0 0 0 0 0 0 0 0 0 0 0 0
 -6.2625  3.1468  1.8126 H 0 0 0 0 0 0 0 0 0 0 0 0
 -6.1176  3.6642  0.1060 H 0 0 0 0 0 0 0 0 0 0 0 0
 -8.7786 -0.0513 -0.3118 O 0 0 0 0 0 0 0 0 0 0 0 0
 -9.0358  0.8466 -0.0611 H 0 0 0 0 0 0 0 0 0 0 0 0
 -7.3537 -2.1613 -0.8341 H 0 0 0 0 0 0 0 0 0 0 0 0
 -4.8918 -2.3170 -0.6722 H 0 0 0 0 0 0 0 0 0 0 0 0
 -2.7151  0.6460  0.4109 H 0 0 0 0 0 0 0 0 0 0 0 0
 -2.7119 -2.2966 -0.4289 H 0 0 0 0 0 0 0 0 0 0 0 0
 -0.4422 -0.0064  0.3496 O 0 0 0 0 0 0 0 0 0 0 0 0
  0.4903 -0.0588  0.5948 H 0 0 0 0 0 0 0 0 0 0 0 0
```

```
-0.5935  -3.2887  -0.3148 H   0  0  0  0  0  0  0  0  0  0  0  0
 1.8962  -3.5130   0.1674 O   0  0  0  0  0  0  0  0  0  0  0  0
 1.7326  -0.3849  -0.8652 H   0  0  0  0  0  0  0  0  0  0  0  0
 3.8632  -2.0942   0.4892 H   0  0  0  0  0  0  0  0  0  0  0  0
 3.2221   1.1909  -1.3727 H   0  0  0  0  0  0  0  0  0  0  0  0
 4.9685   2.9226  -1.6560 H   0  0  0  0  0  0  0  0  0  0  0  0
 7.3906   2.7955  -0.7261 O   0  0  0  0  0  0  0  0  0  0  0  0
 8.1932   2.4819  -0.2864 H   0  0  0  0  0  0  0  0  0  0  0  0
 8.3674  -1.4346   0.7416 H   0  0  0  0  0  0  0  0  0  0  0  0
 9.4767  -0.3577   1.6290 H   0  0  0  0  0  0  0  0  0  0  0  0
 7.8403  -0.6614   2.2665 H   0  0  0  0  0  0  0  0  0  0  0  0
1 46 1 0 0 0 0
1 47 1 0 0 0 0
2 1 1 0 0 0 0
3 8 1 0 0 0 0
3 2 1 0 0 0 0
4 3 2 0 0 0 0
5 6 2 0 0 0 0
5 4 1 0 0 0 0
6 7 1 0 0 0 0
7 10 1 0 0 0 0
7 8 2 0 0 0 0
8 9 1 0 0 0 0
10 40 1 0 0 0 0
11 12 1 0 0 0 0
11 10 2 0 0 0 0
12 38 2 0 0 0 0
13 12 1 0 0 0 0
13 14 2 0 0 0 0
14 35 1 0 0 0 0
15 14 1 0 0 0 0
15 16 2 0 0 0 0
16 33 1 0 0 0 0
17 16 1 0 0 0 0
17 22 2 0 0 0 0
18 17 1 0 0 0 0
19 18 2 0 0 0 0
19 20 1 0 0 0 0
20 21 2 0 0 0 0
21 22 1 0 0 0 0
21 24 1 0 0 0 0
22 23 1 0 0 0 0
24 25 1 0 0 0 0
25 26 1 0 0 0 0
25 27 1 0 0 0 0
28 25 1 0 0 0 0
29 20 1 0 0 0 0
29 30 1 0 0 0 0
31 19 1 0 0 0 0
32 18 1 0 0 0 0
```

```
34 15  1  0  0  0  0
35 36  1  0  0  0  0
37 13  1  0  0  0  0
39 11  1  0  0  0  0
41  6  1  0  0  0  0
42  5  1  0  0  0  0
43  4  1  0  0  0  0
43 44  1  0  0  0  0
45  1  1  0  0  0  0
M  END
```

1B:DM-Curc S0

```
53 54  0  0  0  0  0  0  0  0  0999 V2000
    9.2180    1.4284   -0.9729 C   0  0  0  0  0  0  0  0  0  0  0  0
    8.4339    1.2354    0.2141 O   0  0  0  0  0  0  0  0  0  0  0  0
    7.3838    0.3573    0.0765 C   0  0  0  0  0  0  0  0  0  0  0  0
    7.5970   -1.0248   -0.0617 C   0  0  0  0  0  0  0  0  0  0  0  0
    6.4955   -1.8725   -0.1968 C   0  0  0  0  0  0  0  0  0  0  0  0
    5.2032   -1.3717   -0.1722 C   0  0  0  0  0  0  0  0  0  0  0  0
    4.9694    0.0043   -0.0006 C   0  0  0  0  0  0  0  0  0  0  0  0
    6.0850    0.8499    0.1149 C   0  0  0  0  0  0  0  0  0  0  0  0
    5.9528    1.9192    0.2364 H   0  0  0  0  0  0  0  0  0  0  0  0
    3.6370    0.5969    0.0518 C   0  0  0  0  0  0  0  0  0  0  0  0
    2.4531   -0.0379   -0.0531 C   0  0  0  0  0  0  0  0  0  0  0  0
    1.1790    0.6542    0.0175 C   0  0  0  0  0  0  0  0  0  0  0  0
   -0.0237   -0.0151   -0.0880 C   0  0  0  0  0  0  0  0  0  0  0  0
   -1.2714    0.6997   -0.0088 C   0  0  0  0  0  0  0  0  0  0  0  0
   -2.5189   -0.0757   -0.1229 C   0  0  0  0  0  0  0  0  0  0  0  0
   -3.7207    0.5240   -0.0437 C   0  0  0  0  0  0  0  0  0  0  0  0
   -5.0410   -0.0890   -0.1314 C   0  0  0  0  0  0  0  0  0  0  0  0
   -5.2473   -1.4552   -0.3668 C   0  0  0  0  0  0  0  0  0  0  0  0
   -6.5357   -1.9677   -0.4241 C   0  0  0  0  0  0  0  0  0  0  0  0
   -7.6519   -1.1496   -0.2629 C   0  0  0  0  0  0  0  0  0  0  0  0
   -7.4635    0.2337   -0.0483 C   0  0  0  0  0  0  0  0  0  0  0  0
   -6.1691    0.7399    0.0223 C   0  0  0  0  0  0  0  0  0  0  0  0
   -6.0089    1.7971    0.1882 H   0  0  0  0  0  0  0  0  0  0  0  0
   -8.5949    0.9882    0.0596 O   0  0  0  0  0  0  0  0  0  0  0  0
   -8.4592    2.3949    0.2199 C   0  0  0  0  0  0  0  0  0  0  0  0
   -9.4739    2.7874    0.2562 H   0  0  0  0  0  0  0  0  0  0  0  0
   -7.9399    2.6454    1.1513 H   0  0  0  0  0  0  0  0  0  0  0  0
   -7.9276    2.8430   -0.6262 H   0  0  0  0  0  0  0  0  0  0  0  0
   -8.8899   -1.7122   -0.3944 O   0  0  0  0  0  0  0  0  0  0  0  0
   -9.7693   -1.6345    0.7356 C   0  0  0  0  0  0  0  0  0  0  0  0
  -10.6500   -2.2159    0.4651 H   0  0  0  0  0  0  0  0  0  0  0  0
   -9.2985   -2.0797    1.6190 H   0  0  0  0  0  0  0  0  0  0  0  0
  -10.0561   -0.6042    0.9484 H   0  0  0  0  0  0  0  0  0  0  0  0
   -6.7126   -3.0210   -0.6066 H   0  0  0  0  0  0  0  0  0  0  0  0
   -4.4051   -2.1225   -0.5002 H   0  0  0  0  0  0  0  0  0  0  0  0
   -3.7002    1.6005    0.1092 H   0  0  0  0  0  0  0  0  0  0  0  0
```

-2.4203 -1.1461 -0.2685 H 0 0 0 0 0 0 0 0 0 0 0 0
-1.2997 1.9451 0.1550 O 0 0 0 0 0 0 0 0 0 0 0 0
-0.0212 -1.0872 -0.2285 H 0 0 0 0 0 0 0 0 0 0 0 0
1.2221 1.9699 0.1910 O 0 0 0 0 0 0 0 0 0 0 0 0
0.2590 2.2677 0.2169 H 0 0 0 0 0 0 0 0 0 0 0 0
2.3937 -1.1108 -0.1959 H 0 0 0 0 0 0 0 0 0 0 0 0
3.6049 1.6731 0.1927 H 0 0 0 0 0 0 0 0 0 0 0 0
4.3719 -2.0583 -0.2745 H 0 0 0 0 0 0 0 0 0 0 0 0
6.6838 -2.9328 -0.3183 H 0 0 0 0 0 0 0 0 0 0 0 0
8.8578 -1.5630 -0.1138 O 0 0 0 0 0 0 0 0 0 0 0 0
9.6161 -1.4774 1.1038 C 0 0 0 0 0 0 0 0 0 0 0 0
9.1125 -2.0285 1.9047 H 0 0 0 0 0 0 0 0 0 0 0 0
10.5775 -1.9442 0.8925 H 0 0 0 0 0 0 0 0 0 0 0 0
9.7629 -0.4388 1.4073 H 0 0 0 0 0 0 0 0 0 0 0 0
9.6710 0.4910 -1.3048 H 0 0 0 0 0 0 0 0 0 0 0 0
8.6007 1.8438 -1.7763 H 0 0 0 0 0 0 0 0 0 0 0 0
9.9962 2.1436 -0.7088 H 0 0 0 0 0 0 0 0 0 0 0 0
1 53 1 0 0 0 0
1 2 1 0 0 0 0
3 8 1 0 0 0 0
3 2 1 0 0 0 0
4 3 2 0 0 0 0
5 6 2 0 0 0 0
5 4 1 0 0 0 0
6 7 1 0 0 0 0
7 10 1 0 0 0 0
7 8 2 0 0 0 0
8 9 1 0 0 0 0
10 43 1 0 0 0 0
11 12 1 0 0 0 0
11 10 2 0 0 0 0
12 40 1 0 0 0 0
13 14 1 0 0 0 0
13 12 2 0 0 0 0
14 38 2 0 0 0 0
15 16 2 0 0 0 0
15 14 1 0 0 0 0
16 36 1 0 0 0 0
17 16 1 0 0 0 0
17 22 2 0 0 0 0
18 17 1 0 0 0 0
19 18 2 0 0 0 0
19 20 1 0 0 0 0
20 21 2 0 0 0 0
21 22 1 0 0 0 0
21 24 1 0 0 0 0
22 23 1 0 0 0 0
24 25 1 0 0 0 0
25 26 1 0 0 0 0
25 27 1 0 0 0 0

28 25 1 0 0 0 0
29 20 1 0 0 0 0
29 30 1 0 0 0 0
30 33 1 0 0 0 0
30 32 1 0 0 0 0
31 30 1 0 0 0 0
34 19 1 0 0 0 0
35 18 1 0 0 0 0
37 15 1 0 0 0 0
39 13 1 0 0 0 0
40 41 1 0 0 0 0
42 11 1 0 0 0 0
44 6 1 0 0 0 0
45 5 1 0 0 0 0
46 4 1 0 0 0 0
46 47 1 0 0 0 0
47 50 1 0 0 0 0
47 48 1 0 0 0 0
49 47 1 0 0 0 0
51 1 1 0 0 0 0
52 1 1 0 0 0 0
M END

2B:BDeM-Curc S0

39 40 0 0 0 0 0 0 0 0999 V2000
-5.2485 -1.2645 -0.0008 C 0 0 0 0 0 0 0 0 0 0 0 0
-6.5315 -1.7799 -0.0005 C 0 0 0 0 0 0 0 0 0 0 0 0
-7.6331 -0.9151 0.0004 C 0 0 0 0 0 0 0 0 0 0 0 0
-7.4277 0.4656 0.0010 C 0 0 0 0 0 0 0 0 0 0 0 0
-6.1335 0.9702 0.0007 C 0 0 0 0 0 0 0 0 0 0 0 0
-5.0127 0.1248 -0.0001 C 0 0 0 0 0 0 0 0 0 0 0 0
-3.6826 0.7178 -0.0003 C 0 0 0 0 0 0 0 0 0 0 0 0
-2.4895 0.0948 -0.0004 C 0 0 0 0 0 0 0 0 0 0 0 0
-1.2316 0.8614 -0.0006 C 0 0 0 0 0 0 0 0 0 0 0 0
0.0068 0.1276 -0.0004 C 0 0 0 0 0 0 0 0 0 0 0 0
1.2181 0.7915 -0.0003 C 0 0 0 0 0 0 0 0 0 0 0 0
2.4850 0.0846 -0.0002 C 0 0 0 0 0 0 0 0 0 0 0 0
3.6747 0.7193 -0.0002 C 0 0 0 0 0 0 0 0 0 0 0 0
5.0039 0.1244 -0.0000 C 0 0 0 0 0 0 0 0 0 0 0 0
6.1259 0.9686 0.0004 C 0 0 0 0 0 0 0 0 0 0 0 0
7.4195 0.4629 0.0006 C 0 0 0 0 0 0 0 0 0 0 0 0
7.6242 -0.9178 0.0004 C 0 0 0 0 0 0 0 0 0 0 0 0
6.5218 -1.7815 -0.0001 C 0 0 0 0 0 0 0 0 0 0 0 0
5.2391 -1.2652 -0.0003 C 0 0 0 0 0 0 0 0 0 0 0 0
4.4030 -1.9544 -0.0006 H 0 0 0 0 0 0 0 0 0 0 0 0
6.6986 -2.8500 -0.0002 H 0 0 0 0 0 0 0 0 0 0 0 0
8.8624 -1.4840 0.0005 O 0 0 0 0 0 0 0 0 0 0 0 0
9.5317 -0.7914 0.0009 H 0 0 0 0 0 0 0 0 0 0 0 0
8.2691 1.1395 0.0009 H 0 0 0 0 0 0 0 0 0 0 0 0

5.9775 2.0430 0.0005 H 0 0 0 0 0 0 0 0 0 0 0 0
3.6465 1.8051 -0.0003 H 0 0 0 0 0 0 0 0 0 0 0 0
2.4163 -0.9973 -0.0001 H 0 0 0 0 0 0 0 0 0 0 0 0
1.2734 2.1184 -0.0003 O 0 0 0 0 0 0 0 0 0 0 0 0
0.3117 2.4251 -0.0004 H 0 0 0 0 0 0 0 0 0 0 0 0
-0.0032 -0.9535 -0.0003 H 0 0 0 0 0 0 0 0 0 0 0 0
-1.2450 2.1181 -0.0004 O 0 0 0 0 0 0 0 0 0 0 0 0
-2.4055 -0.9868 -0.0003 H 0 0 0 0 0 0 0 0 0 0 0 0
-3.6452 1.8048 -0.0003 H 0 0 0 0 0 0 0 0 0 0 0 0
-5.9832 2.0443 0.0012 H 0 0 0 0 0 0 0 0 0 0 0 0
-8.2770 1.1426 0.0017 H 0 0 0 0 0 0 0 0 0 0 0 0
-8.8719 -1.4800 0.0006 O 0 0 0 0 0 0 0 0 0 0 0 0
-9.5402 -0.7865 0.0010 H 0 0 0 0 0 0 0 0 0 0 0 0
-6.7096 -2.8483 -0.0011 H 0 0 0 0 0 0 0 0 0 0 0 0
-4.4117 -1.9527 -0.0015 H 0 0 0 0 0 0 0 0 0 0 0 0
1 2 2 0 0 0 0
1 6 1 0 0 0 0
2 3 1 0 0 0 0
3 36 1 0 0 0 0
3 4 2 0 0 0 0
4 35 1 0 0 0 0
5 4 1 0 0 0 0
5 34 1 0 0 0 0
6 5 2 0 0 0 0
7 33 1 0 0 0 0
7 6 1 0 0 0 0
8 32 1 0 0 0 0
8 7 2 0 0 0 0
9 10 1 0 0 0 0
9 31 2 0 0 0 0
9 8 1 0 0 0 0
10 11 2 0 0 0 0
10 30 1 0 0 0 0
11 28 1 0 0 0 0
11 12 1 0 0 0 0
12 13 2 0 0 0 0
12 27 1 0 0 0 0
13 14 1 0 0 0 0
14 15 2 0 0 0 0
15 25 1 0 0 0 0
15 16 1 0 0 0 0
16 24 1 0 0 0 0
17 22 1 0 0 0 0
17 16 2 0 0 0 0
18 17 1 0 0 0 0
19 18 2 0 0 0 0
19 14 1 0 0 0 0
20 19 1 0 0 0 0
21 18 1 0 0 0 0
22 23 1 0 0 0 0

26 13 1 0 0 0 0
29 28 1 0 0 0 0
36 37 1 0 0 0 0
38 2 1 0 0 0 0
39 1 1 0 0 0 0
M END

3B: CY S0

49 51 0 0 0 0 0 0 0 0999 V2000
1.2574 0.8426 0.6088 C 0 0 0 0 0 0 0 0 0 0 0 0
-0.0002 1.1189 1.4339 C 0 0 0 0 0 0 0 0 0 0 0 0
-0.0002 2.1570 1.7804 H 0 0 0 0 0 0 0 0 0 0 0 0
-1.2575 0.8427 0.6084 C 0 0 0 0 0 0 0 0 0 0 0 0
-1.2948 -0.5801 0.0960 C 0 0 0 0 0 0 0 0 0 0 0 0
-2.4196 -1.2882 -0.1598 C 0 0 0 0 0 0 0 0 0 0 0 0
-2.2350 -2.3230 -0.4366 H 0 0 0 0 0 0 0 0 0 0 0 0
-3.8300 -0.9223 -0.1036 C 0 0 0 0 0 0 0 0 0 0 0 0
-4.7696 -1.9599 0.0158 C 0 0 0 0 0 0 0 0 0 0 0 0
-6.1346 -1.7058 0.0797 C 0 0 0 0 0 0 0 0 0 0 0 0
-6.6022 -0.4011 -0.0011 C 0 0 0 0 0 0 0 0 0 0 0 0
-5.6795 0.6536 -0.1537 C 0 0 0 0 0 0 0 0 0 0 0 0
-4.3187 0.4009 -0.2011 C 0 0 0 0 0 0 0 0 0 0 0 0
-3.6315 1.2176 -0.3556 H 0 0 0 0 0 0 0 0 0 0 0 0
-6.2763 1.8874 -0.2528 O 0 0 0 0 0 0 0 0 0 0 0 0
-5.4503 3.0323 -0.4190 C 0 0 0 0 0 0 0 0 0 0 0 0
-4.7671 3.1537 0.4283 H 0 0 0 0 0 0 0 0 0 0 0 0
-4.8743 2.9737 -1.3485 H 0 0 0 0 0 0 0 0 0 0 0 0
-6.1248 3.8855 -0.4650 H 0 0 0 0 0 0 0 0 0 0 0 0
-7.9327 -0.1423 0.0487 O 0 0 0 0 0 0 0 0 0 0 0 0
-8.0417 0.8144 -0.0410 H 0 0 0 0 0 0 0 0 0 0 0 0
-6.8544 -2.5087 0.1824 H 0 0 0 0 0 0 0 0 0 0 0 0
-4.4169 -2.9834 0.0682 H 0 0 0 0 0 0 0 0 0 0 0 0
-0.0000 -1.3163 -0.1200 C 0 0 0 0 0 0 0 0 0 0 0 0
1.2947 -0.5800 0.0960 C 0 0 0 0 0 0 0 0 0 0 0 0
2.4195 -1.2881 -0.1602 C 0 0 0 0 0 0 0 0 0 0 0 0
3.8300 -0.9222 -0.1041 C 0 0 0 0 0 0 0 0 0 0 0 0
4.7695 -1.9599 0.0139 C 0 0 0 0 0 0 0 0 0 0 0 0
6.1345 -1.7061 0.0775 C 0 0 0 0 0 0 0 0 0 0 0 0
6.6022 -0.4013 -0.0020 C 0 0 0 0 0 0 0 0 0 0 0 0
5.6795 0.6536 -0.1533 C 0 0 0 0 0 0 0 0 0 0 0 0
4.3188 0.4011 -0.2004 C 0 0 0 0 0 0 0 0 0 0 0 0
3.6315 1.2181 -0.3538 H 0 0 0 0 0 0 0 0 0 0 0 0
6.2764 1.8875 -0.2512 O 0 0 0 0 0 0 0 0 0 0 0 0
5.4504 3.0327 -0.4154 C 0 0 0 0 0 0 0 0 0 0 0 0
4.8739 2.9754 -1.3448 H 0 0 0 0 0 0 0 0 0 0 0 0
4.7677 3.1529 0.4324 H 0 0 0 0 0 0 0 0 0 0 0 0
6.1250 3.8859 -0.4606 H 0 0 0 0 0 0 0 0 0 0 0 0
7.9328 -0.1426 0.0475 O 0 0 0 0 0 0 0 0 0 0 0 0
8.0418 0.8142 -0.0411 H 0 0 0 0 0 0 0 0 0 0 0 0

```
    6.8543   -2.5091    0.1791 H   0  0  0  0  0  0  0  0  0  0  0  0
    4.4168   -2.9834    0.0654 H   0  0  0  0  0  0  0  0  0  0  0  0
    2.2349   -2.3227   -0.4373 H   0  0  0  0  0  0  0  0  0  0  0  0
   -0.0000   -2.4860   -0.4866 O   0  0  0  0  0  0  0  0  0  0  0  0
   -1.2849    1.5533   -0.2317 H   0  0  0  0  0  0  0  0  0  0  0  0
   -2.1487    1.0397    1.2102 H   0  0  0  0  0  0  0  0  0  0  0  0
   -0.0004    0.4836    2.3269 H   0  0  0  0  0  0  0  0  0  0  0  0
    2.1484    1.0392    1.2111 H   0  0  0  0  0  0  0  0  0  0  0  0
    1.2853    1.5534   -0.2310 H   0  0  0  0  0  0  0  0  0  0  0  0
  1 48  1  0  0  0  0
  1  2  1  0  0  0  0
  2  3  1  0  0  0  0
  2 47  1  0  0  0  0
  4 46  1  0  0  0  0
  4  2  1  0  0  0  0
  5  4  1  0  0  0  0
  6  8  1  0  0  0  0
  6  5  2  0  0  0  0
  7  6  1  0  0  0  0
  8  9  2  0  0  0  0
  9 23  1  0  0  0  0
  9 10  1  0  0  0  0
 10 22  1  0  0  0  0
 11 20  1  0  0  0  0
 11 10  2  0  0  0  0
 12 11  1  0  0  0  0
 13 12  2  0  0  0  0
 13  8  1  0  0  0  0
 14 13  1  0  0  0  0
 15 12  1  0  0  0  0
 16 15  1  0  0  0  0
 16 17  1  0  0  0  0
 18 16  1  0  0  0  0
 19 16  1  0  0  0  0
 21 20  1  0  0  0  0
 24  5  1  0  0  0  0
 24 25  1  0  0  0  0
 25  1  1  0  0  0  0
 26 27  1  0  0  0  0
 26 25  2  0  0  0  0
 27 28  2  0  0  0  0
 28 42  1  0  0  0  0
 28 29  1  0  0  0  0
 29 41  1  0  0  0  0
 30 39  1  0  0  0  0
 30 29  2  0  0  0  0
 31 30  1  0  0  0  0
 32 31  2  0  0  0  0
 32 27  1  0  0  0  0
 33 32  1  0  0  0  0
```

```
34 31  1  0  0  0  0
35 34  1  0  0  0  0
35 37  1  0  0  0  0
36 35  1  0  0  0  0
38 35  1  0  0  0  0
40 39  1  0  0  0  0
43 26  1  0  0  0  0
44 24  2  0  0  0  0
45  4  1  0  0  0  0
49  1  1  0  0  0  0
M  END
```

3B': O-CY S0

```
49 51  0  0  0  0  0  0  0  0999 V2000
    1.2573    0.8500    0.6012 C   0  0  0  0  0  0  0  0  0  0  0  0
   -0.0003    1.1258    1.4263 C   0  0  0  0  0  0  0  0  0  0  0  0
   -0.0004    2.1638    1.7729 H   0  0  0  0  0  0  0  0  0  0  0  0
   -1.2576    0.8500    0.6007 C   0  0  0  0  0  0  0  0  0  0  0  0
   -1.2951   -0.5726    0.0880 C   0  0  0  0  0  0  0  0  0  0  0  0
   -2.4192   -1.2812   -0.1680 C   0  0  0  0  0  0  0  0  0  0  0  0
   -2.2341   -2.3154   -0.4463 H   0  0  0  0  0  0  0  0  0  0  0  0
   -3.8305   -0.9165   -0.1087 C   0  0  0  0  0  0  0  0  0  0  0  0
   -4.7659   -1.9531    0.0133 C   0  0  0  0  0  0  0  0  0  0  0  0
   -6.1288   -1.6846    0.0828 C   0  0  0  0  0  0  0  0  0  0  0  0
   -6.6031   -0.3805    0.0057 C   0  0  0  0  0  0  0  0  0  0  0  0
   -5.6831    0.6804   -0.1530 C   0  0  0  0  0  0  0  0  0  0  0  0
   -4.3228    0.4050   -0.2039 C   0  0  0  0  0  0  0  0  0  0  0  0
   -3.6320    1.2174   -0.3615 H   0  0  0  0  0  0  0  0  0  0  0  0
   -6.2375    1.9190   -0.2524 O   0  0  0  0  0  0  0  0  0  0  0  0
   -5.3739    3.0322   -0.4217 C   0  0  0  0  0  0  0  0  0  0  0  0
   -4.6832    3.1383    0.4228 H   0  0  0  0  0  0  0  0  0  0  0  0
   -4.8003    2.9596   -1.3528 H   0  0  0  0  0  0  0  0  0  0  0  0
   -6.0232    3.9049   -0.4670 H   0  0  0  0  0  0  0  0  0  0  0  0
   -7.9233   -0.0572    0.0582 O   0  0  0  0  0  0  0  0  0  0  0  0
   -8.4361   -0.8661    0.1587 H   0  0  0  0  0  0  0  0  0  0  0  0
   -6.8388   -2.4999    0.1900 H   0  0  0  0  0  0  0  0  0  0  0  0
   -4.4197   -2.9786    0.0650 H   0  0  0  0  0  0  0  0  0  0  0  0
   -0.0000   -1.3090   -0.1280 C   0  0  0  0  0  0  0  0  0  0  0  0
    1.2949   -0.5724    0.0880 C   0  0  0  0  0  0  0  0  0  0  0  0
    2.4191   -1.2808   -0.1686 C   0  0  0  0  0  0  0  0  0  0  0  0
    3.8304   -0.9163   -0.1096 C   0  0  0  0  0  0  0  0  0  0  0  0
    4.7658   -1.9532    0.0102 C   0  0  0  0  0  0  0  0  0  0  0  0
    6.1287   -1.6851    0.0792 C   0  0  0  0  0  0  0  0  0  0  0  0
    6.6032   -0.3809    0.0041 C   0  0  0  0  0  0  0  0  0  0  0  0
    5.6832    0.6805   -0.1521 C   0  0  0  0  0  0  0  0  0  0  0  0
    4.3229    0.4054   -0.2028 C   0  0  0  0  0  0  0  0  0  0  0  0
    3.6321    1.2182   -0.3584 H   0  0  0  0  0  0  0  0  0  0  0  0
    6.2378    1.9191   -0.2497 O   0  0  0  0  0  0  0  0  0  0  0  0
    5.3743    3.0329   -0.4156 C   0  0  0  0  0  0  0  0  0  0  0  0
```

```
  4.7998   2.9626  -1.3464 H  0  0  0  0  0  0  0  0  0  0  0  0
  4.6843   3.1371   0.4298 H  0  0  0  0  0  0  0  0  0  0  0  0
  6.0236   3.9055  -0.4595 H  0  0  0  0  0  0  0  0  0  0  0  0
  7.9235  -0.0578   0.0565 O  0  0  0  0  0  0  0  0  0  0  0  0
  8.4362  -0.8670   0.1546 H  0  0  0  0  0  0  0  0  0  0  0  0
  6.8386  -2.5006   0.1847 H  0  0  0  0  0  0  0  0  0  0  0  0
  4.4194  -2.9787   0.0603 H  0  0  0  0  0  0  0  0  0  0  0  0
  2.2339  -2.3149  -0.4474 H  0  0  0  0  0  0  0  0  0  0  0  0
  0.0000  -2.4791  -0.4937 O  0  0  0  0  0  0  0  0  0  0  0  0
 -1.2845   1.5601  -0.2396 H  0  0  0  0  0  0  0  0  0  0  0  0
 -2.1491   1.0475   1.2016 H  0  0  0  0  0  0  0  0  0  0  0  0
 -0.0005   0.4905   2.3194 H  0  0  0  0  0  0  0  0  0  0  0  0
  2.1484   1.0473   1.2025 H  0  0  0  0  0  0  0  0  0  0  0  0
  1.2846   1.5602  -0.2389 H  0  0  0  0  0  0  0  0  0  0  0  0
 1 48  1  0  0  0  0
 1  2  1  0  0  0  0
 2  3  1  0  0  0  0
 2 47  1  0  0  0  0
 4 46  1  0  0  0  0
 4  2  1  0  0  0  0
 5  4  1  0  0  0  0
 6  8  1  0  0  0  0
 6  5  2  0  0  0  0
 7  6  1  0  0  0  0
 8  9  2  0  0  0  0
 9 23  1  0  0  0  0
 9 10  1  0  0  0  0
10 22  1  0  0  0  0
11 20  1  0  0  0  0
11 10  2  0  0  0  0
12 11  1  0  0  0  0
13 12  2  0  0  0  0
13  8  1  0  0  0  0
14 13  1  0  0  0  0
15 12  1  0  0  0  0
16 15  1  0  0  0  0
16 17  1  0  0  0  0
18 16  1  0  0  0  0
19 16  1  0  0  0  0
20 21  1  0  0  0  0
24  5  1  0  0  0  0
24 25  1  0  0  0  0
25  1  1  0  0  0  0
26 27  1  0  0  0  0
26 25  2  0  0  0  0
27 28  2  0  0  0  0
28 42  1  0  0  0  0
28 29  1  0  0  0  0
29 41  1  0  0  0  0
30 39  1  0  0  0  0
```

```
30 29  2  0  0  0  0
31 30  1  0  0  0  0
32 31  2  0  0  0  0
32 27  1  0  0  0  0
33 32  1  0  0  0  0
34 31  1  0  0  0  0
35 34  1  0  0  0  0
35 37  1  0  0  0  0
36 35  1  0  0  0  0
38 35  1  0  0  0  0
39 40  1  0  0  0  0
43 26  1  0  0  0  0
44 24  2  0  0  0  0
45  4  1  0  0  0  0
49  1  1  0  0  0  0
M  END
```

1A: CCE-Curc S1

```
47 48  0  0  0  0  0  0  0  0999 V2000
    8.5487   -2.2371    0.0001 C   0  0  0  0  0  0  0  0  0  0  0  0
    8.5891   -0.8053    0.0002 O   0  0  0  0  0  0  0  0  0  0  0  0
    7.4220   -0.1175    0.0001 C   0  0  0  0  0  0  0  0  0  0  0  0
    7.6107    1.2999    0.0002 C   0  0  0  0  0  0  0  0  0  0  0  0
    6.4952    2.1489    0.0001 C   0  0  0  0  0  0  0  0  0  0  0  0
    5.2253    1.6091    0.0000 C   0  0  0  0  0  0  0  0  0  0  0  0
    5.0124    0.1950   -0.0000 C   0  0  0  0  0  0  0  0  0  0  0  0
    6.1438   -0.6507    0.0000 C   0  0  0  0  0  0  0  0  0  0  0  0
    5.9918   -1.7231   -0.0000 H   0  0  0  0  0  0  0  0  0  0  0  0
    3.6940   -0.4098   -0.0001 C   0  0  0  0  0  0  0  0  0  0  0  0
    2.5058    0.2396   -0.0002 C   0  0  0  0  0  0  0  0  0  0  0  0
    1.2134   -0.5251   -0.0003 C   0  0  0  0  0  0  0  0  0  0  0  0
    0.0289    0.2030   -0.0003 C   0  0  0  0  0  0  0  0  0  0  0  0
   -1.2418   -0.4691   -0.0002 C   0  0  0  0  0  0  0  0  0  0  0  0
   -2.4725    0.2075   -0.0001 C   0  0  0  0  0  0  0  0  0  0  0  0
   -3.7094   -0.4325   -0.0001 C   0  0  0  0  0  0  0  0  0  0  0  0
   -5.0009    0.1799   -0.0000 C   0  0  0  0  0  0  0  0  0  0  0  0
   -5.2121    1.5855   -0.0000 C   0  0  0  0  0  0  0  0  0  0  0  0
   -6.4904    2.1275    0.0001 C   0  0  0  0  0  0  0  0  0  0  0  0
   -7.6128    1.2993    0.0001 C   0  0  0  0  0  0  0  0  0  0  0  0
   -7.4314   -0.1018    0.0001 C   0  0  0  0  0  0  0  0  0  0  0  0
   -6.1620   -0.6496    0.0000 C   0  0  0  0  0  0  0  0  0  0  0  0
   -6.0268   -1.7255   -0.0000 H   0  0  0  0  0  0  0  0  0  0  0  0
   -8.6189   -0.8046    0.0001 O   0  0  0  0  0  0  0  0  0  0  0  0
   -8.5533   -2.2208    0.0001 C   0  0  0  0  0  0  0  0  0  0  0  0
   -9.5853   -2.5755    0.0001 H   0  0  0  0  0  0  0  0  0  0  0  0
   -8.0392   -2.5974   -0.8930 H   0  0  0  0  0  0  0  0  0  0  0  0
   -8.0391   -2.5975    0.8931 H   0  0  0  0  0  0  0  0  0  0  0  0
   -8.8701    1.8323    0.0002 O   0  0  0  0  0  0  0  0  0  0  0  0
   -9.4863    1.0833    0.0002 H   0  0  0  0  0  0  0  0  0  0  0  0
```

-6.6440 3.2022 0.0001 H 0 0 0 0 0 0 0 0 0 0 0 0
-4.3597 2.2560 -0.0000 H 0 0 0 0 0 0 0 0 0 0 0 0
-3.6903 -1.5193 -0.0001 H 0 0 0 0 0 0 0 0 0 0 0 0
-2.4134 1.2916 -0.0001 H 0 0 0 0 0 0 0 0 0 0 0 0
-1.2300 -1.8067 -0.0001 O 0 0 0 0 0 0 0 0 0 0 0 0
-0.2524 -2.0781 -0.0001 H 0 0 0 0 0 0 0 0 0 0 0 0
0.0449 1.2865 -0.0002 H 0 0 0 0 0 0 0 0 0 0 0 0
1.3007 -1.8035 -0.0002 O 0 0 0 0 0 0 0 0 0 0 0 0
2.4429 1.3256 -0.0001 H 0 0 0 0 0 0 0 0 0 0 0 0
3.6359 -1.4957 -0.0002 H 0 0 0 0 0 0 0 0 0 0 0 0
4.3702 2.2740 -0.0000 H 0 0 0 0 0 0 0 0 0 0 0 0
6.6604 3.2203 0.0002 H 0 0 0 0 0 0 0 0 0 0 0 0
8.8486 1.8117 0.0003 O 0 0 0 0 0 0 0 0 0 0 0 0
9.4826 1.0735 0.0003 H 0 0 0 0 0 0 0 0 0 0 0 0
8.0420 -2.6082 -0.8961 H 0 0 0 0 0 0 0 0 0 0 0 0
9.5878 -2.5633 0.0002 H 0 0 0 0 0 0 0 0 0 0 0 0
8.0418 -2.6082 0.8962 H 0 0 0 0 0 0 0 0 0 0 0 0
1 46 1 0 0 0 0
1 2 1 0 0 0 0
1 47 1 0 0 0 0
3 2 1 0 0 0 0
3 4 2 0 0 0 0
4 43 1 0 0 0 0
5 4 1 0 0 0 0
5 42 1 0 0 0 0
6 5 2 0 0 0 0
7 8 2 0 0 0 0
7 6 1 0 0 0 0
8 3 1 0 0 0 0
9 8 1 0 0 0 0
10 7 1 0 0 0 0
11 39 1 0 0 0 0
11 10 2 0 0 0 0
12 13 1 0 0 0 0
12 38 2 0 0 0 0
12 11 1 0 0 0 0
13 37 1 0 0 0 0
13 14 1 0 0 0 0
14 15 2 0 0 0 0
14 35 1 0 0 0 0
15 34 1 0 0 0 0
15 16 1 0 0 0 0
16 17 1 0 0 0 0
17 18 2 0 0 0 0
17 22 1 0 0 0 0
18 19 1 0 0 0 0
19 31 1 0 0 0 0
19 20 2 0 0 0 0
20 29 1 0 0 0 0
21 20 1 0 0 0 0

21 24 1 0 0 0 0
22 21 2 0 0 0 0
23 22 1 0 0 0 0
25 24 1 0 0 0 0
25 26 1 0 0 0 0
25 28 1 0 0 0 0
27 25 1 0 0 0 0
29 30 1 0 0 0 0
32 18 1 0 0 0 0
33 16 1 0 0 0 0
35 36 1 0 0 0 0
40 10 1 0 0 0 0
41 6 1 0 0 0 0
43 44 1 0 0 0 0
45 1 1 0 0 0 0
M END

2A: CCE-Curc-Conf S0

47 48 0 0 0 0 0 0 0 0999 V2000
-5.8461 3.4550 0.0013 C 0 0 0 0 0 0 0 0 0 0 0 0
-6.8772 2.4753 0.0005 O 0 0 0 0 0 0 0 0 0 0 0 0
-6.5235 1.1493 0.0001 C 0 0 0 0 0 0 0 0 0 0 0 0
-7.6357 0.2767 -0.0003 C 0 0 0 0 0 0 0 0 0 0 0 0
-7.4261 -1.0944 -0.0007 C 0 0 0 0 0 0 0 0 0 0 0 0
-6.1287 -1.5984 -0.0006 C 0 0 0 0 0 0 0 0 0 0 0 0
-5.0141 -0.7491 -0.0002 C 0 0 0 0 0 0 0 0 0 0 0 0
-5.2368 0.6463 0.0002 C 0 0 0 0 0 0 0 0 0 0 0 0
-4.3927 1.3220 0.0006 H 0 0 0 0 0 0 0 0 0 0 0 0
-3.6831 -1.3371 -0.0002 C 0 0 0 0 0 0 0 0 0 0 0 0
-2.4892 -0.7139 -0.0000 C 0 0 0 0 0 0 0 0 0 0 0 0
-1.2312 -1.4790 0.0000 C 0 0 0 0 0 0 0 0 0 0 0 0
0.0074 -0.7451 0.0001 C 0 0 0 0 0 0 0 0 0 0 0 0
1.2194 -1.4087 0.0001 C 0 0 0 0 0 0 0 0 0 0 0 0
2.4863 -0.7027 0.0001 C 0 0 0 0 0 0 0 0 0 0 0 0
3.6770 -1.3374 0.0002 C 0 0 0 0 0 0 0 0 0 0 0 0
5.0069 -0.7475 0.0002 C 0 0 0 0 0 0 0 0 0 0 0 0
5.2293 0.6482 -0.0002 C 0 0 0 0 0 0 0 0 0 0 0 0
6.5157 1.1519 -0.0002 C 0 0 0 0 0 0 0 0 0 0 0 0
7.6287 0.2802 0.0003 C 0 0 0 0 0 0 0 0 0 0 0 0
7.4195 -1.0910 0.0007 C 0 0 0 0 0 0 0 0 0 0 0 0
6.1226 -1.5958 0.0006 C 0 0 0 0 0 0 0 0 0 0 0 0
5.9696 -2.6689 0.0009 H 0 0 0 0 0 0 0 0 0 0 0 0
8.2813 -1.7469 0.0010 H 0 0 0 0 0 0 0 0 0 0 0 0
8.8862 0.7852 0.0003 O 0 0 0 0 0 0 0 0 0 0 0 0
8.8093 1.7494 -0.0000 H 0 0 0 0 0 0 0 0 0 0 0 0
6.8683 2.4779 -0.0006 O 0 0 0 0 0 0 0 0 0 0 0 0
5.8369 3.4574 -0.0013 C 0 0 0 0 0 0 0 0 0 0 0 0
5.2117 3.3719 -0.8962 H 0 0 0 0 0 0 0 0 0 0 0 0
5.2113 3.3727 0.8935 H 0 0 0 0 0 0 0 0 0 0 0 0

```
6.3395 4.4229 -0.0016 H 0 0 0 0 0 0 0 0 0 0 0 0
4.3861 1.3250 -0.0006 H 0 0 0 0 0 0 0 0 0 0 0 0
3.6488 -2.4230 0.0003 H 0 0 0 0 0 0 0 0 0 0 0 0
2.4164 0.3793 0.0001 H 0 0 0 0 0 0 0 0 0 0 0 0
1.2745 -2.7355 0.0001 O 0 0 0 0 0 0 0 0 0 0 0 0
0.3129 -3.0425 0.0001 H 0 0 0 0 0 0 0 0 0 0 0 0
-0.0028 0.3362 0.0001 H 0 0 0 0 0 0 0 0 0 0 0 0
-1.2436 -2.7361 -0.0001 O 0 0 0 0 0 0 0 0 0 0 0 0
-2.4041 0.3680 0.0001 H 0 0 0 0 0 0 0 0 0 0 0 0
-3.6457 -2.4238 -0.0003 H 0 0 0 0 0 0 0 0 0 0 0 0
-5.9740 -2.6712 -0.0010 H 0 0 0 0 0 0 0 0 0 0 0 0
-8.2877 -1.7507 -0.0011 H 0 0 0 0 0 0 0 0 0 0 0 0
-8.8936 0.7812 -0.0004 O 0 0 0 0 0 0 0 0 0 0 0 0
-8.8168 1.7454 -0.0000 H 0 0 0 0 0 0 0 0 0 0 0 0
-6.3489 4.4204 0.0017 H 0 0 0 0 0 0 0 0 0 0 0 0
-5.2209 3.3694 0.8962 H 0 0 0 0 0 0 0 0 0 0 0 0
-5.2203 3.3704 -0.8933 H 0 0 0 0 0 0 0 0 0 0 0 0
1 45 1 0 0 0 0
1 46 1 0 0 0 0
2 1 1 0 0 0 0
3 8 1 0 0 0 0
3 2 1 0 0 0 0
4 3 2 0 0 0 0
5 6 2 0 0 0 0
5 4 1 0 0 0 0
6 7 1 0 0 0 0
7 10 1 0 0 0 0
7 8 2 0 0 0 0
8 9 1 0 0 0 0
10 11 2 0 0 0 0
11 12 1 0 0 0 0
11 39 1 0 0 0 0
12 13 1 0 0 0 0
13 14 2 0 0 0 0
13 37 1 0 0 0 0
14 35 1 0 0 0 0
14 15 1 0 0 0 0
15 16 2 0 0 0 0
16 17 1 0 0 0 0
16 33 1 0 0 0 0
17 22 2 0 0 0 0
18 19 2 0 0 0 0
18 17 1 0 0 0 0
19 20 1 0 0 0 0
20 25 1 0 0 0 0
20 21 2 0 0 0 0
21 24 1 0 0 0 0
22 21 1 0 0 0 0
22 23 1 0 0 0 0
26 25 1 0 0 0 0
```

```
27 19  1  0  0  0  0
28 27  1  0  0  0  0
28 30  1  0  0  0  0
29 28  1  0  0  0  0
31 28  1  0  0  0  0
32 18  1  0  0  0  0
34 15  1  0  0  0  0
35 36  1  0  0  0  0
38 12  2  0  0  0  0
40 10  1  0  0  0  0
41  6  1  0  0  0  0
42  5  1  0  0  0  0
43  4  1  0  0  0  0
43 44  1  0  0  0  0
47  1  1  0  0  0  0
M  END
```

2A: CCE-Curc-Conf S1

```
47 48  0  0  0  0  0  0  0  0999 V2000
    5.6899    3.4512   -0.0005 C   0  0  0  0  0  0  0  0  0  0  0  0
    6.7612    2.5216   -0.0003 O   0  0  0  0  0  0  0  0  0  0  0  0
    6.4566    1.1781   -0.0001 C   0  0  0  0  0  0  0  0  0  0  0  0
    7.6049    0.3515    0.0000 C   0  0  0  0  0  0  0  0  0  0  0  0
    7.4462   -1.0360    0.0002 C   0  0  0  0  0  0  0  0  0  0  0  0
    6.1750   -1.5897    0.0002 C   0  0  0  0  0  0  0  0  0  0  0  0
    5.0053   -0.7835    0.0001 C   0  0  0  0  0  0  0  0  0  0  0  0
    5.1862    0.6323   -0.0001 C   0  0  0  0  0  0  0  0  0  0  0  0
    4.3181    1.2792   -0.0002 H   0  0  0  0  0  0  0  0  0  0  0  0
    3.7189   -1.4086    0.0002 C   0  0  0  0  0  0  0  0  0  0  0  0
    2.4763   -0.7937    0.0001 C   0  0  0  0  0  0  0  0  0  0  0  0
    1.2581   -1.5347    0.0002 C   0  0  0  0  0  0  0  0  0  0  0  0
   -0.0164   -0.7985    0.0001 C   0  0  0  0  0  0  0  0  0  0  0  0
   -1.2264   -1.4793    0.0000 C   0  0  0  0  0  0  0  0  0  0  0  0
   -2.4787   -0.7685   -0.0000 C   0  0  0  0  0  0  0  0  0  0  0  0
   -3.6979   -1.3950   -0.0001 C   0  0  0  0  0  0  0  0  0  0  0  0
   -4.9924   -0.7640   -0.0001 C   0  0  0  0  0  0  0  0  0  0  0  0
   -5.1731    0.6464    0.0001 C   0  0  0  0  0  0  0  0  0  0  0  0
   -6.4445    1.1868    0.0001 C   0  0  0  0  0  0  0  0  0  0  0  0
   -7.5908    0.3431   -0.0002 C   0  0  0  0  0  0  0  0  0  0  0  0
   -7.4276   -1.0460   -0.0004 C   0  0  0  0  0  0  0  0  0  0  0  0
   -6.1531   -1.5854   -0.0003 C   0  0  0  0  0  0  0  0  0  0  0  0
   -6.0266   -2.6633   -0.0005 H   0  0  0  0  0  0  0  0  0  0  0  0
   -8.3125   -1.6728   -0.0005 H   0  0  0  0  0  0  0  0  0  0  0  0
   -8.8248    0.8821   -0.0002 O   0  0  0  0  0  0  0  0  0  0  0  0
   -8.7240    1.8491   -0.0000 H   0  0  0  0  0  0  0  0  0  0  0  0
   -6.7651    2.5142    0.0003 O   0  0  0  0  0  0  0  0  0  0  0  0
   -5.7089    3.4716    0.0005 C   0  0  0  0  0  0  0  0  0  0  0  0
   -5.0860    3.3680    0.8961 H   0  0  0  0  0  0  0  0  0  0  0  0
   -5.0858    3.3684   -0.8949 H   0  0  0  0  0  0  0  0  0  0  0  0
```

```
-6.1893 4.4499 0.0007 H 0 0 0 0 0 0 0 0 0 0 0 0
-4.3088 1.2977 0.0003 H 0 0 0 0 0 0 0 0 0 0 0 0
-3.6857 -2.4814 -0.0001 H 0 0 0 0 0 0 0 0 0 0 0 0
-2.4034 0.3153 0.0000 H 0 0 0 0 0 0 0 0 0 0 0 0
-1.2702 -2.8127 -0.0000 O 0 0 0 0 0 0 0 0 0 0 0 0
-0.2687 -3.0832 -0.0000 H 0 0 0 0 0 0 0 0 0 0 0 0
-0.0180 0.2859 0.0001 H 0 0 0 0 0 0 0 0 0 0 0 0
1.2066 -2.8133 -0.0000 O 0 0 0 0 0 0 0 0 0 0 0 0
2.3898 0.2896 0.0001 H 0 0 0 0 0 0 0 0 0 0 0 0
3.7078 -2.4960 0.0002 H 0 0 0 0 0 0 0 0 0 0 0 0
6.0619 -2.6698 0.0004 H 0 0 0 0 0 0 0 0 0 0 0 0
8.3347 -1.6590 0.0003 H 0 0 0 0 0 0 0 0 0 0 0 0
8.8488 0.9041 0.0000 O 0 0 0 0 0 0 0 0 0 0 0 0
8.7276 1.8669 -0.0001 H 0 0 0 0 0 0 0 0 0 0 0 0
6.1454 4.4426 -0.0006 H 0 0 0 0 0 0 0 0 0 0 0 0
5.0637 3.3380 -0.8942 H 0 0 0 0 0 0 0 0 0 0 0 0
5.0636 3.3382 0.8932 H 0 0 0 0 0 0 0 0 0 0 0 0
1 2 1 0 0 0 0
1 47 1 0 0 0 0
2 3 1 0 0 0 0
3 8 2 0 0 0 0
3 4 1 0 0 0 0
4 5 2 0 0 0 0
5 6 1 0 0 0 0
5 42 1 0 0 0 0
6 41 1 0 0 0 0
7 10 1 0 0 0 0
7 6 2 0 0 0 0
8 7 1 0 0 0 0
9 8 1 0 0 0 0
10 40 1 0 0 0 0
11 10 2 0 0 0 0
11 12 1 0 0 0 0
13 12 1 0 0 0 0
14 13 2 0 0 0 0
15 34 1 0 0 0 0
15 14 1 0 0 0 0
16 15 2 0 0 0 0
17 16 1 0 0 0 0
17 18 2 0 0 0 0
18 32 1 0 0 0 0
19 18 1 0 0 0 0
19 27 1 0 0 0 0
20 19 2 0 0 0 0
21 22 2 0 0 0 0
21 20 1 0 0 0 0
22 17 1 0 0 0 0
23 22 1 0 0 0 0
24 21 1 0 0 0 0
25 20 1 0 0 0 0
```

```
25 26  1  0  0  0  0
27 28  1  0  0  0  0
28 31  1  0  0  0  0
28 29  1  0  0  0  0
30 28  1  0  0  0  0
33 16  1  0  0  0  0
35 14  1  0  0  0  0
36 35  1  0  0  0  0
37 13  1  0  0  0  0
38 12  2  0  0  0  0
39 11  1  0  0  0  0
43  4  1  0  0  0  0
44 43  1  0  0  0  0
45  1  1  0  0  0  0
46  1  1  0  0  0  0
M  END
```

3B: CY S1

```
49 51  0  0  0  0  0  0  0  0999 V2000
    1.2610    0.8902    0.6198 C   0  0  0  0  0  0  0  0  0  0  0  0
   -0.0000    1.2125    1.4245 C   0  0  0  0  0  0  0  0  0  0  0  0
   -0.0000    2.2692    1.7155 H   0  0  0  0  0  0  0  0  0  0  0  0
   -1.2610    0.8902    0.6197 C   0  0  0  0  0  0  0  0  0  0  0  0
   -1.2683   -0.5416    0.1288 C   0  0  0  0  0  0  0  0  0  0  0  0
   -2.4087   -1.2962   -0.1032 C   0  0  0  0  0  0  0  0  0  0  0  0
   -2.2348   -2.3484   -0.3161 H   0  0  0  0  0  0  0  0  0  0  0  0
   -3.8081   -0.9383   -0.0722 C   0  0  0  0  0  0  0  0  0  0  0  0
   -4.7552   -1.9833    0.0358 C   0  0  0  0  0  0  0  0  0  0  0  0
   -6.1225   -1.7371    0.0661 C   0  0  0  0  0  0  0  0  0  0  0  0
   -6.5975   -0.4311   -0.0252 C   0  0  0  0  0  0  0  0  0  0  0  0
   -5.6745    0.6290   -0.1607 C   0  0  0  0  0  0  0  0  0  0  0  0
   -4.3101    0.3868   -0.1876 C   0  0  0  0  0  0  0  0  0  0  0  0
   -3.6268    1.2098   -0.3378 H   0  0  0  0  0  0  0  0  0  0  0  0
   -6.2778    1.8610   -0.2697 O   0  0  0  0  0  0  0  0  0  0  0  0
   -5.4518    3.0046   -0.4258 C   0  0  0  0  0  0  0  0  0  0  0  0
   -4.7756    3.1278    0.4292 H   0  0  0  0  0  0  0  0  0  0  0  0
   -4.8605    2.9471   -1.3479 H   0  0  0  0  0  0  0  0  0  0  0  0
   -6.1248    3.8612   -0.4811 H   0  0  0  0  0  0  0  0  0  0  0  0
   -7.9326   -0.1788    0.0014 O   0  0  0  0  0  0  0  0  0  0  0  0
   -8.0413    0.7810   -0.0894 H   0  0  0  0  0  0  0  0  0  0  0  0
   -6.8398   -2.5458    0.1577 H   0  0  0  0  0  0  0  0  0  0  0  0
   -4.3992   -3.0066    0.1070 H   0  0  0  0  0  0  0  0  0  0  0  0
   -0.0000   -1.1737   -0.0868 C   0  0  0  0  0  0  0  0  0  0  0  0
    1.2683   -0.5416    0.1288 C   0  0  0  0  0  0  0  0  0  0  0  0
    2.4087   -1.2961   -0.1032 C   0  0  0  0  0  0  0  0  0  0  0  0
    3.8081   -0.9383   -0.0723 C   0  0  0  0  0  0  0  0  0  0  0  0
    4.7552   -1.9833    0.0355 C   0  0  0  0  0  0  0  0  0  0  0  0
    6.1225   -1.7371    0.0658 C   0  0  0  0  0  0  0  0  0  0  0  0
    6.5975   -0.4311   -0.0253 C   0  0  0  0  0  0  0  0  0  0  0  0
    5.6745    0.6290   -0.1606 C   0  0  0  0  0  0  0  0  0  0  0  0
```

```
4.3101 0.3868 -0.1875 C 0 0 0 0 0 0 0 0 0 0 0 0
3.6268 1.2098 -0.3375 H 0 0 0 0 0 0 0 0 0 0 0 0
6.2778 1.8610 -0.2695 O 0 0 0 0 0 0 0 0 0 0 0 0
5.4518 3.0046 -0.4253 C 0 0 0 0 0 0 0 0 0 0 0 0
4.8604 2.9474 -1.3473 H 0 0 0 0 0 0 0 0 0 0 0 0
4.7757 3.1277 0.4298 H 0 0 0 0 0 0 0 0 0 0 0 0
6.1248 3.8613 -0.4804 H 0 0 0 0 0 0 0 0 0 0 0 0
7.9326 -0.1789 0.0012 O 0 0 0 0 0 0 0 0 0 0 0 0
8.0413 0.7810 -0.0894 H 0 0 0 0 0 0 0 0 0 0 0 0
6.8398 -2.5458 0.1571 H 0 0 0 0 0 0 0 0 0 0 0 0
4.3991 -3.0066 0.1066 H 0 0 0 0 0 0 0 0 0 0 0 0
2.2348 -2.3484 -0.3163 H 0 0 0 0 0 0 0 0 0 0 0 0
-0.0000 -2.3975 -0.5735 O 0 0 0 0 0 0 0 0 0 0 0 0
-1.3376 1.5925 -0.2251 H 0 0 0 0 0 0 0 0 0 0 0 0
-2.1450 1.0542 1.2439 H 0 0 0 0 0 0 0 0 0 0 0 0
-0.0001 0.6219 2.3483 H 0 0 0 0 0 0 0 0 0 0 0 0
2.1449 1.0542 1.2441 H 0 0 0 0 0 0 0 0 0 0 0 0
1.3377 1.5925 -0.2250 H 0 0 0 0 0 0 0 0 0 0 0 0
1 48 1 0 0 0 0
1 2 1 0 0 0 0
2 3 1 0 0 0 0
2 47 1 0 0 0 0
4 46 1 0 0 0 0
4 2 1 0 0 0 0
5 4 1 0 0 0 0
6 8 1 0 0 0 0
6 5 2 0 0 0 0
7 6 1 0 0 0 0
8 9 2 0 0 0 0
9 10 1 0 0 0 0
9 23 1 0 0 0 0
10 22 1 0 0 0 0
11 20 1 0 0 0 0
11 10 2 0 0 0 0
12 11 1 0 0 0 0
13 12 2 0 0 0 0
13 8 1 0 0 0 0
14 13 1 0 0 0 0
15 12 1 0 0 0 0
16 15 1 0 0 0 0
16 17 1 0 0 0 0
18 16 1 0 0 0 0
19 16 1 0 0 0 0
21 20 1 0 0 0 0
24 25 2 0 0 0 0
24 5 1 0 0 0 0
25 1 1 0 0 0 0
26 27 1 0 0 0 0
26 25 1 0 0 0 0
27 28 2 0 0 0 0
```

28 29 1 0 0 0 0
28 42 1 0 0 0 0
29 41 1 0 0 0 0
30 39 1 0 0 0 0
30 29 2 0 0 0 0
31 30 1 0 0 0 0
32 31 2 0 0 0 0
32 27 1 0 0 0 0
33 32 1 0 0 0 0
34 31 1 0 0 0 0
35 34 1 0 0 0 0
35 37 1 0 0 0 0
36 35 1 0 0 0 0
38 35 1 0 0 0 0
40 39 1 0 0 0 0
43 26 1 0 0 0 0
44 24 1 0 0 0 0
45 4 1 0 0 0 0
49 1 1 0 0 0 0
M END

3B': O-CY S1

49 51 0 0 0 0 0 0 0 0999 V2000
1.2614 0.9010 0.6106 C 0 0 0 0 0 0 0 0 0 0 0 0
0.0000 1.2252 1.4141 C 0 0 0 0 0 0 0 0 0 0 0 0
0.0000 2.2826 1.7022 H 0 0 0 0 0 0 0 0 0 0 0 0
-1.2613 0.9011 0.6105 C 0 0 0 0 0 0 0 0 0 0 0 0
-1.2685 -0.5319 0.1225 C 0 0 0 0 0 0 0 0 0 0 0 0
-2.4077 -1.2881 -0.1078 C 0 0 0 0 0 0 0 0 0 0 0 0
-2.2322 -2.3400 -0.3215 H 0 0 0 0 0 0 0 0 0 0 0 0
-3.8083 -0.9327 -0.0743 C 0 0 0 0 0 0 0 0 0 0 0 0
-4.7509 -1.9771 0.0360 C 0 0 0 0 0 0 0 0 0 0 0 0
-6.1165 -1.7174 0.0687 C 0 0 0 0 0 0 0 0 0 0 0 0
-6.5992 -0.4129 -0.0212 C 0 0 0 0 0 0 0 0 0 0 0 0
-5.6789 0.6539 -0.1613 C 0 0 0 0 0 0 0 0 0 0 0 0
-4.3149 0.3900 -0.1893 C 0 0 0 0 0 0 0 0 0 0 0 0
-3.6282 1.2088 -0.3424 H 0 0 0 0 0 0 0 0 0 0 0 0
-6.2411 1.8913 -0.2720 O 0 0 0 0 0 0 0 0 0 0 0 0
-5.3782 3.0050 -0.4239 C 0 0 0 0 0 0 0 0 0 0 0 0
-4.6980 3.1113 0.4314 H 0 0 0 0 0 0 0 0 0 0 0 0
-4.7851 2.9373 -1.3454 H 0 0 0 0 0 0 0 0 0 0 0 0
-6.0263 3.8808 -0.4785 H 0 0 0 0 0 0 0 0 0 0 0 0
-7.9245 -0.0964 0.0042 O 0 0 0 0 0 0 0 0 0 0 0 0
-8.4331 -0.9131 0.0954 H 0 0 0 0 0 0 0 0 0 0 0 0
-6.8244 -2.5384 0.1641 H 0 0 0 0 0 0 0 0 0 0 0 0
-4.4010 -3.0023 0.1077 H 0 0 0 0 0 0 0 0 0 0 0 0
0.0000 -1.1642 -0.0928 C 0 0 0 0 0 0 0 0 0 0 0 0
1.2685 -0.5319 0.1225 C 0 0 0 0 0 0 0 0 0 0 0 0
2.4077 -1.2880 -0.1081 C 0 0 0 0 0 0 0 0 0 0 0 0

3.8083	-0.9327	-0.0746	C	0	0	0	0	0	0	0	0	0	0	0	0
4.7509	-1.9771	0.0350	C	0	0	0	0	0	0	0	0	0	0	0	0
6.1165	-1.7175	0.0677	C	0	0	0	0	0	0	0	0	0	0	0	0
6.5992	-0.4129	-0.0216	C	0	0	0	0	0	0	0	0	0	0	0	0
5.6789	0.6539	-0.1611	C	0	0	0	0	0	0	0	0	0	0	0	0
4.3149	0.3900	-0.1890	C	0	0	0	0	0	0	0	0	0	0	0	0
3.6282	1.2090	-0.3416	H	0	0	0	0	0	0	0	0	0	0	0	0
6.2411	1.8914	-0.2712	O	0	0	0	0	0	0	0	0	0	0	0	0
5.3782	3.0052	-0.4223	C	0	0	0	0	0	0	0	0	0	0	0	0
4.7849	2.9380	-1.3437	H	0	0	0	0	0	0	0	0	0	0	0	0
4.6982	3.1110	0.4332	H	0	0	0	0	0	0	0	0	0	0	0	0
6.0263	3.8810	-0.4765	H	0	0	0	0	0	0	0	0	0	0	0	0
7.9245	-0.0965	0.0038	O	0	0	0	0	0	0	0	0	0	0	0	0
8.4331	-0.9133	0.0945	H	0	0	0	0	0	0	0	0	0	0	0	0
6.8244	-2.5385	0.1626	H	0	0	0	0	0	0	0	0	0	0	0	0
4.4010	-3.0024	0.1063	H	0	0	0	0	0	0	0	0	0	0	0	0
2.2322	-2.3399	-0.3220	H	0	0	0	0	0	0	0	0	0	0	0	0
-0.0001	-2.3886	-0.5777	O	0	0	0	0	0	0	0	0	0	0	0	0
-1.3386	1.6009	-0.2362	H	0	0	0	0	0	0	0	0	0	0	0	0
-2.1453	1.0669	1.2342	H	0	0	0	0	0	0	0	0	0	0	0	0
-0.0001	0.6372	2.3397	H	0	0	0	0	0	0	0	0	0	0	0	0
2.1453	1.0667	1.2344	H	0	0	0	0	0	0	0	0	0	0	0	0
1.3389	1.6009	-0.2360	H	0	0	0	0	0	0	0	0	0	0	0	0

1	48	1	0	0	0	0
1	2	1	0	0	0	0
2	3	1	0	0	0	0
2	47	1	0	0	0	0
4	46	1	0	0	0	0
4	2	1	0	0	0	0
5	4	1	0	0	0	0
6	8	1	0	0	0	0
6	5	2	0	0	0	0
7	6	1	0	0	0	0
8	9	2	0	0	0	0
9	10	1	0	0	0	0
9	23	1	0	0	0	0
10	22	1	0	0	0	0
11	20	1	0	0	0	0
11	10	2	0	0	0	0
12	11	1	0	0	0	0
13	12	2	0	0	0	0
13	8	1	0	0	0	0
14	13	1	0	0	0	0
15	12	1	0	0	0	0
16	15	1	0	0	0	0
16	17	1	0	0	0	0
18	16	1	0	0	0	0
19	16	1	0	0	0	0
20	21	1	0	0	0	0
24	25	2	0	0	0	0

```
24 5 1 0 0 0 0
25 1 1 0 0 0 0
26 27 1 0 0 0 0
26 25 1 0 0 0 0
27 28 2 0 0 0 0
28 29 1 0 0 0 0
28 42 1 0 0 0 0
29 41 1 0 0 0 0
30 39 1 0 0 0 0
30 29 2 0 0 0 0
31 30 1 0 0 0 0
32 31 2 0 0 0 0
32 27 1 0 0 0 0
33 32 1 0 0 0 0
34 31 1 0 0 0 0
35 34 1 0 0 0 0
35 37 1 0 0 0 0
36 35 1 0 0 0 0
38 35 1 0 0 0 0
39 40 1 0 0 0 0
43 26 1 0 0 0 0
44 24 1 0 0 0 0
45 4 1 0 0 0 0
49 1 1 0 0 0 0
M END
```

Curcuminoid radicals

1A: CCE-Curc S_0

```
47 48 0 0 0 0 0 0 0 0999 V2000
  -8.4773   2.2854   0.0001 C  0 0 0 0 0 0 0 0 0 0 0 0
  -8.5754   0.8658   0.0001 O  0 0 0 0 0 0 0 0 0 0 0 0
  -7.4167   0.1301   0.0000 C  0 0 0 0 0 0 0 0 0 0 0 0
  -7.6335  -1.2616   0.0000 C  0 0 0 0 0 0 0 0 0 0 0 0
  -6.5418  -2.1237  -0.0000 C  0 0 0 0 0 0 0 0 0 0 0 0
  -5.2487  -1.6172  -0.0000 C  0 0 0 0 0 0 0 0 0 0 0 0
  -5.0125  -0.2342  -0.0000 C  0 0 0 0 0 0 0 0 0 0 0 0
  -6.1264   0.6326   0.0000 C  0 0 0 0 0 0 0 0 0 0 0 0
  -5.9539   1.7012   0.0000 H  0 0 0 0 0 0 0 0 0 0 0 0
  -3.6816   0.3568  -0.0000 C  0 0 0 0 0 0 0 0 0 0 0 0
  -2.4882  -0.2666  -0.0001 C  0 0 0 0 0 0 0 0 0 0 0 0
  -1.2314   0.5002  -0.0001 C  0 0 0 0 0 0 0 0 0 0 0 0
   0.0071  -0.2330  -0.0001 C  0 0 0 0 0 0 0 0 0 0 0 0
   1.2188   0.4306  -0.0001 C  0 0 0 0 0 0 0 0 0 0 0 0
   2.4851  -0.2760  -0.0001 C  0 0 0 0 0 0 0 0 0 0 0 0
   3.6749   0.3596  -0.0000 C  0 0 0 0 0 0 0 0 0 0 0 0
   5.0049  -0.2332   0.0000 C  0 0 0 0 0 0 0 0 0 0 0 0
   5.2405  -1.6164   0.0000 C  0 0 0 0 0 0 0 0 0 0 0 0
   6.5332  -2.1238   0.0001 C  0 0 0 0 0 0 0 0 0 0 0 0
   7.6258  -1.2631   0.0001 C  0 0 0 0 0 0 0 0 0 0 0 0
```

7.4099 0.1288 0.0000 C 0 0 0 0 0 0 0 0 0 0 0 0
6.1200 0.6325 0.0000 C 0 0 0 0 0 0 0 0 0 0 0 0
5.9497 1.7015 -0.0000 H 0 0 0 0 0 0 0 0 0 0 0 0
8.5689 0.8635 0.0000 O 0 0 0 0 0 0 0 0 0 0 0 0
8.4727 2.2833 -0.0000 C 0 0 0 0 0 0 0 0 0 0 0 0
9.4962 2.6536 -0.0000 H 0 0 0 0 0 0 0 0 0 0 0 0
7.9546 2.6438 -0.8946 H 0 0 0 0 0 0 0 0 0 0 0 0
7.9546 2.6438 0.8946 H 0 0 0 0 0 0 0 0 0 0 0 0
8.8875 -1.7594 0.0001 O 0 0 0 0 0 0 0 0 0 0 0 0
9.4931 -1.0052 0.0001 H 0 0 0 0 0 0 0 0 0 0 0 0
6.7193 -3.1909 0.0001 H 0 0 0 0 0 0 0 0 0 0 0 0
4.4083 -2.3092 0.0001 H 0 0 0 0 0 0 0 0 0 0 0 0
3.6429 1.4454 0.0000 H 0 0 0 0 0 0 0 0 0 0 0 0
2.4174 -1.3578 -0.0001 H 0 0 0 0 0 0 0 0 0 0 0 0
1.2732 1.7583 -0.0000 O 0 0 0 0 0 0 0 0 0 0 0 0
0.3108 2.0636 -0.0000 H 0 0 0 0 0 0 0 0 0 0 0 0
-0.0031 -1.3142 -0.0001 H 0 0 0 0 0 0 0 0 0 0 0 0
-1.2446 1.7580 -0.0000 O 0 0 0 0 0 0 0 0 0 0 0 0
-2.4045 -1.3481 -0.0001 H 0 0 0 0 0 0 0 0 0 0 0 0
-3.6407 1.4437 -0.0000 H 0 0 0 0 0 0 0 0 0 0 0 0
-4.4158 -2.3091 -0.0001 H 0 0 0 0 0 0 0 0 0 0 0 0
-6.7292 -3.1905 -0.0000 H 0 0 0 0 0 0 0 0 0 0 0 0
-8.8958 -1.7567 0.0000 O 0 0 0 0 0 0 0 0 0 0 0 0
-9.5003 -1.0016 0.0001 H 0 0 0 0 0 0 0 0 0 0 0 0
-7.9586 2.6453 -0.8944 H 0 0 0 0 0 0 0 0 0 0 0 0
-9.5004 2.6571 0.0001 H 0 0 0 0 0 0 0 0 0 0 0 0
-7.9586 2.6452 0.8946 H 0 0 0 0 0 0 0 0 0 0 0 0
1 46 1 0 0 0 0
1 47 1 0 0 0 0
2 1 1 0 0 0 0
3 2 1 0 0 0 0
4 3 2 0 0 0 0
4 43 1 0 0 0 0
5 4 1 0 0 0 0
6 5 2 0 0 0 0
6 7 1 0 0 0 0
7 8 2 0 0 0 0
8 3 1 0 0 0 0
8 9 1 0 0 0 0
10 7 1 0 0 0 0
10 40 1 0 0 0 0
11 10 2 0 0 0 0
12 11 1 0 0 0 0
12 38 2 0 0 0 0
13 12 1 0 0 0 0
13 14 2 0 0 0 0
14 15 1 0 0 0 0
14 35 1 0 0 0 0
15 16 2 0 0 0 0
16 33 1 0 0 0 0

16 17 1 0 0 0 0
17 18 1 0 0 0 0
18 32 1 0 0 0 0
18 19 2 0 0 0 0
19 31 1 0 0 0 0
20 19 1 0 0 0 0
20 29 1 0 0 0 0
21 24 1 0 0 0 0
21 20 2 0 0 0 0
22 17 2 0 0 0 0
22 21 1 0 0 0 0
23 22 1 0 0 0 0
25 26 1 0 0 0 0
25 24 1 0 0 0 0
25 28 1 0 0 0 0
27 25 1 0 0 0 0
30 29 1 0 0 0 0
34 15 1 0 0 0 0
35 36 1 0 0 0 0
37 13 1 0 0 0 0
39 11 1 0 0 0 0
41 6 1 0 0 0 0
42 5 1 0 0 0 0
43 44 1 0 0 0 0
45 1 1 0 0 0 0
M END

3A:T-DIK-Curc S_0

47 48 0 0 0 0 0 0 0 0999 V2000
-0.0025 3.2765 -0.2923 C 0 0 0 0 0 0 0 0 0 0 0 0
-0.9756 2.3132 -0.9800 C 0 0 0 0 0 0 0 0 0 0 0 0
-2.0359 1.7492 -0.1217 C 0 0 0 0 0 0 0 0 0 0 0 0
-2.9698 0.9268 -0.6386 C 0 0 0 0 0 0 0 0 0 0 0 0
-4.0816 0.2793 0.0395 C 0 0 0 0 0 0 0 0 0 0 0 0
-4.3325 0.4115 1.4138 C 0 0 0 0 0 0 0 0 0 0 0 0
-5.4091 -0.2315 2.0090 C 0 0 0 0 0 0 0 0 0 0 0 0
-6.2629 -1.0227 1.2468 C 0 0 0 0 0 0 0 0 0 0 0 0
-6.0265 -1.1681 -0.1347 C 0 0 0 0 0 0 0 0 0 0 0 0
-4.9520 -0.5264 -0.7261 C 0 0 0 0 0 0 0 0 0 0 0 0
-4.7643 -0.6340 -1.7867 H 0 0 0 0 0 0 0 0 0 0 0 0
-6.9407 -1.9736 -0.7654 O 0 0 0 0 0 0 0 0 0 0 0 0
-6.8051 -2.1862 -2.1662 C 0 0 0 0 0 0 0 0 0 0 0 0
-7.6266 -2.8408 -2.4514 H 0 0 0 0 0 0 0 0 0 0 0 0
-6.8816 -1.2432 -2.7172 H 0 0 0 0 0 0 0 0 0 0 0 0
-5.8528 -2.6727 -2.4014 H 0 0 0 0 0 0 0 0 0 0 0 0
-7.3137 -1.6493 1.8283 O 0 0 0 0 0 0 0 0 0 0 0 0
-7.7754 -2.1411 1.1349 H 0 0 0 0 0 0 0 0 0 0 0 0
-5.6078 -0.1343 3.0694 H 0 0 0 0 0 0 0 0 0 0 0 0
-3.6802 1.0200 2.0275 H 0 0 0 0 0 0 0 0 0 0 0 0

```
-2.8770 0.7249 -1.7040 H 0 0 0 0 0 0 0 0 0 0 0 0
-2.0217 2.0250 0.9269 H 0 0 0 0 0 0 0 0 0 0 0 0
-0.8412 2.0305 -2.1588 O 0 0 0 0 0 0 0 0 0 0 0 0
1.0072 2.4999 0.5626 C 0 0 0 0 0 0 0 0 0 0 0 0
2.0291 1.7402 -0.1840 C 0 0 0 0 0 0 0 0 0 0 0 0
2.9940 1.0653 0.4707 C 0 0 0 0 0 0 0 0 0 0 0 0
4.0743 0.2657 -0.0854 C 0 0 0 0 0 0 0 0 0 0 0 0
4.9858 -0.3389 0.8071 C 0 0 0 0 0 0 0 0 0 0 0 0
6.0309 -1.1137 0.3341 C 0 0 0 0 0 0 0 0 0 0 0 0
6.1952 -1.3086 -1.0517 C 0 0 0 0 0 0 0 0 0 0 0 0
5.3010 -0.7166 -1.9382 C 0 0 0 0 0 0 0 0 0 0 0 0
4.2540 0.0606 -1.4620 C 0 0 0 0 0 0 0 0 0 0 0 0
3.5688 0.5087 -2.1704 H 0 0 0 0 0 0 0 0 0 0 0 0
5.4445 -0.8799 -2.9993 H 0 0 0 0 0 0 0 0 0 0 0 0
7.2164 -2.0674 -1.5170 O 0 0 0 0 0 0 0 0 0 0 0 0
7.7135 -2.3827 -0.7493 H 0 0 0 0 0 0 0 0 0 0 0 0
6.9794 -1.7516 1.0932 O 0 0 0 0 0 0 0 0 0 0 0 0
6.9164 -1.6202 2.5089 C 0 0 0 0 0 0 0 0 0 0 0 0
5.9787 -2.0263 2.9019 H 0 0 0 0 0 0 0 0 0 0 0 0
7.7530 -2.1954 2.9013 H 0 0 0 0 0 0 0 0 0 0 0 0
7.0192 -0.5734 2.8129 H 0 0 0 0 0 0 0 0 0 0 0 0
4.8531 -0.1864 1.8705 H 0 0 0 0 0 0 0 0 0 0 0 0
2.9565 1.1294 1.5564 H 0 0 0 0 0 0 0 0 0 0 0 0
1.9589 1.7505 -1.2660 H 0 0 0 0 0 0 0 0 0 0 0 0
0.9319 2.5148 1.7793 O 0 0 0 0 0 0 0 0 0 0 0 0
0.5144 3.8337 -1.0757 H 0 0 0 0 0 0 0 0 0 0 0 0
-0.5361 3.9581 0.3717 H 0 0 0 0 0 0 0 0 0 0 0 0
1 47 1 0 0 0 0
1 24 1 0 0 0 0
2 1 1 0 0 0 0
2 3 1 0 0 0 0
3 22 1 0 0 0 0
4 3 2 0 0 0 0
4 5 1 0 0 0 0
5 6 2 0 0 0 0
6 7 1 0 0 0 0
6 20 1 0 0 0 0
7 19 1 0 0 0 0
8 17 1 0 0 0 0
8 7 2 0 0 0 0
9 8 1 0 0 0 0
10 9 2 0 0 0 0
10 5 1 0 0 0 0
11 10 1 0 0 0 0
12 9 1 0 0 0 0
13 12 1 0 0 0 0
14 13 1 0 0 0 0
15 13 1 0 0 0 0
16 13 1 0 0 0 0
18 17 1 0 0 0 0
```

21 4 1 0 0 0 0
23 2 2 0 0 0 0
24 45 2 0 0 0 0
25 26 2 0 0 0 0
25 24 1 0 0 0 0
26 43 1 0 0 0 0
27 26 1 0 0 0 0
27 28 2 0 0 0 0
28 42 1 0 0 0 0
29 28 1 0 0 0 0
29 37 1 0 0 0 0
30 29 2 0 0 0 0
31 32 2 0 0 0 0
31 30 1 0 0 0 0
32 27 1 0 0 0 0
33 32 1 0 0 0 0
34 31 1 0 0 0 0
35 30 1 0 0 0 0
35 36 1 0 0 0 0
37 38 1 0 0 0 0
38 41 1 0 0 0 0
38 40 1 0 0 0 0
38 39 1 0 0 0 0
44 25 1 0 0 0 0
46 1 1 0 0 0 0
M END

4A:OCE-Curc S_0

47 48 0 0 0 0 0 0 0 0999 V2000
-8.6970 -1.9094 -0.0765 C 0 0 0 0 0 0 0 0 0 0 0 0
-8.6289 -0.4966 0.0759 O 0 0 0 0 0 0 0 0 0 0 0 0
-7.3919 0.0992 0.1049 C 0 0 0 0 0 0 0 0 0 0 0 0
-7.4445 1.4979 0.2575 C 0 0 0 0 0 0 0 0 0 0 0 0
-6.2597 2.2248 0.3018 C 0 0 0 0 0 0 0 0 0 0 0 0
-5.0352 1.5771 0.1976 C 0 0 0 0 0 0 0 0 0 0 0 0
-4.9623 0.1845 0.0460 C 0 0 0 0 0 0 0 0 0 0 0 0
-6.1697 -0.5443 0.0015 C 0 0 0 0 0 0 0 0 0 0 0 0
-6.1238 -1.6195 -0.1149 H 0 0 0 0 0 0 0 0 0 0 0 0
-3.7085 -0.5510 -0.0667 C 0 0 0 0 0 0 0 0 0 0 0 0
-2.4519 -0.0738 -0.0518 C 0 0 0 0 0 0 0 0 0 0 0 0
-1.2893 -0.9976 -0.1818 C 0 0 0 0 0 0 0 0 0 0 0 0
0.0219 -0.3340 -0.1682 C 0 0 0 0 0 0 0 0 0 0 0 0
1.2407 -0.9218 -0.3204 C 0 0 0 0 0 0 0 0 0 0 0 0
2.4569 -0.1182 -0.3078 C 0 0 0 0 0 0 0 0 0 0 0 0
3.7048 -0.6124 -0.1734 C 0 0 0 0 0 0 0 0 0 0 0 0
4.9613 0.1298 -0.1752 C 0 0 0 0 0 0 0 0 0 0 0 0
5.0563 1.4840 -0.5263 C 0 0 0 0 0 0 0 0 0 0 0 0
6.2768 2.1472 -0.5017 C 0 0 0 0 0 0 0 0 0 0 0 0
7.4351 1.4751 -0.1286 C 0 0 0 0 0 0 0 0 0 0 0 0

```
7.3605 0.1124 0.2204 C 0 0 0 0 0 0 0 0 0 0 0 0
6.1431 -0.5477 0.1934 C 0 0 0 0 0 0 0 0 0 0 0 0
6.0851 -1.5942 0.4654 H 0 0 0 0 0 0 0 0 0 0 0 0
8.5716 -0.4322 0.5646 O 0 0 0 0 0 0 0 0 0 0 0 0
8.6182 -1.8013 0.9492 C 0 0 0 0 0 0 0 0 0 0 0 0
9.6603 -2.0128 1.1812 H 0 0 0 0 0 0 0 0 0 0 0 0
8.0032 -1.9831 1.8366 H 0 0 0 0 0 0 0 0 0 0 0 0
8.2878 -2.4526 0.1333 H 0 0 0 0 0 0 0 0 0 0 0 0
8.6259 2.1229 -0.1074 O 0 0 0 0 0 0 0 0 0 0 0 0
9.2963 1.4848 0.1730 H 0 0 0 0 0 0 0 0 0 0 0 0
6.3544 3.1919 -0.7770 H 0 0 0 0 0 0 0 0 0 0 0 0
4.1733 2.0282 -0.8372 H 0 0 0 0 0 0 0 0 0 0 0 0
3.8227 -1.6828 -0.0080 H 0 0 0 0 0 0 0 0 0 0 0 0
2.3002 0.9513 -0.3865 H 0 0 0 0 0 0 0 0 0 0 0 0
1.3523 -2.2624 -0.4564 O 0 0 0 0 0 0 0 0 0 0 0 0
2.2271 -2.4662 -0.8052 H 0 0 0 0 0 0 0 0 0 0 0 0
0.0292 0.7406 -0.0314 H 0 0 0 0 0 0 0 0 0 0 0 0
-1.4416 -2.2087 -0.2981 O 0 0 0 0 0 0 0 0 0 0 0 0
-2.2461 0.9867 0.0535 H 0 0 0 0 0 0 0 0 0 0 0 0
-3.7925 -1.6304 -0.1772 H 0 0 0 0 0 0 0 0 0 0 0 0
-4.1269 2.1653 0.2358 H 0 0 0 0 0 0 0 0 0 0 0 0
-6.3204 3.3000 0.4193 H 0 0 0 0 0 0 0 0 0 0 0 0
-8.6409 2.1302 0.3603 O 0 0 0 0 0 0 0 0 0 0 0 0
-9.3277 1.4513 0.3093 H 0 0 0 0 0 0 0 0 0 0 0 0
-8.1996 -2.4212 0.7539 H 0 0 0 0 0 0 0 0 0 0 0 0
-9.7562 -2.1610 -0.0749 H 0 0 0 0 0 0 0 0 0 0 0 0
-8.2483 -2.2268 -1.0235 H 0 0 0 0 0 0 0 0 0 0 0 0
1 46 1 0 0 0 0
1 2 1 0 0 0 0
1 45 1 0 0 0 0
2 3 1 0 0 0 0
3 4 2 0 0 0 0
4 5 1 0 0 0 0
4 43 1 0 0 0 0
5 42 1 0 0 0 0
6 41 1 0 0 0 0
6 5 2 0 0 0 0
7 6 1 0 0 0 0
8 7 2 0 0 0 0
8 3 1 0 0 0 0
9 8 1 0 0 0 0
10 11 2 0 0 0 0
10 7 1 0 0 0 0
11 39 1 0 0 0 0
12 13 1 0 0 0 0
12 11 1 0 0 0 0
13 37 1 0 0 0 0
14 15 1 0 0 0 0
14 13 2 0 0 0 0
15 16 2 0 0 0 0
```

16 33 1 0 0 0 0
17 16 1 0 0 0 0
17 22 2 0 0 0 0
18 19 2 0 0 0 0
18 17 1 0 0 0 0
19 20 1 0 0 0 0
20 29 1 0 0 0 0
20 21 2 0 0 0 0
21 24 1 0 0 0 0
22 21 1 0 0 0 0
22 23 1 0 0 0 0
24 25 1 0 0 0 0
25 26 1 0 0 0 0
25 27 1 0 0 0 0
28 25 1 0 0 0 0
29 30 1 0 0 0 0
31 19 1 0 0 0 0
32 18 1 0 0 0 0
34 15 1 0 0 0 0
35 14 1 0 0 0 0
36 35 1 0 0 0 0
38 12 2 0 0 0 0
40 10 1 0 0 0 0
44 43 1 0 0 0 0
47 1 1 0 0 0 0
M END

5A:T-E-Curc S_0

47 48 0 0 0 0 0 0 0 0999 V2000
8.4372 -0.5324 1.3578 C 0 0 0 0 0 0 0 0 0 0 0 0
8.0226 0.6190 0.6306 O 0 0 0 0 0 0 0 0 0 0 0 0
6.7363 0.6654 0.1563 C 0 0 0 0 0 0 0 0 0 0 0 0
6.4422 1.8421 -0.5606 C 0 0 0 0 0 0 0 0 0 0 0 0
5.1720 2.0147 -1.1013 C 0 0 0 0 0 0 0 0 0 0 0 0
4.1999 1.0374 -0.9330 C 0 0 0 0 0 0 0 0 0 0 0 0
4.4726 -0.1383 -0.2180 C 0 0 0 0 0 0 0 0 0 0 0 0
5.7656 -0.3078 0.3217 C 0 0 0 0 0 0 0 0 0 0 0 0
5.9855 -1.2148 0.8701 H 0 0 0 0 0 0 0 0 0 0 0 0
3.4958 -1.1974 -0.0050 C 0 0 0 0 0 0 0 0 0 0 0 0
2.1915 -1.2184 -0.3416 C 0 0 0 0 0 0 0 0 0 0 0 0
1.3649 -2.4342 -0.0832 C 0 0 0 0 0 0 0 0 0 0 0 0
-0.0973 -2.3367 -0.1708 C 0 0 0 0 0 0 0 0 0 0 0 0
-0.8941 -1.2464 0.0131 C 0 0 0 0 0 0 0 0 0 0 0 0
-2.3358 -1.3278 -0.1220 C 0 0 0 0 0 0 0 0 0 0 0 0
-3.1684 -0.2908 0.1012 C 0 0 0 0 0 0 0 0 0 0 0 0
-4.6208 -0.2745 -0.0230 C 0 0 0 0 0 0 0 0 0 0 0 0
-5.3807 -1.3824 -0.4270 C 0 0 0 0 0 0 0 0 0 0 0 0
-6.7644 -1.3075 -0.5225 C 0 0 0 0 0 0 0 0 0 0 0 0
-7.4273 -0.1236 -0.2180 C 0 0 0 0 0 0 0 0 0 0 0 0

-6.6820 1.0002 0.1876 C 0 0 0 0 0 0 0 0 0 0 0 0
-5.3024 0.9232 0.2824 C 0 0 0 0 0 0 0 0 0 0 0 0
-4.7270 1.7855 0.5943 H 0 0 0 0 0 0 0 0 0 0 0 0
-7.4539 2.1030 0.4571 O 0 0 0 0 0 0 0 0 0 0 0 0
-6.8067 3.2988 0.8746 C 0 0 0 0 0 0 0 0 0 0 0 0
-7.5977 4.0304 1.0294 H 0 0 0 0 0 0 0 0 0 0 0 0
-6.2625 3.1468 1.8126 H 0 0 0 0 0 0 0 0 0 0 0 0
-6.1176 3.6642 0.1060 H 0 0 0 0 0 0 0 0 0 0 0 0
-8.7786 -0.0513 -0.3118 O 0 0 0 0 0 0 0 0 0 0 0 0
-9.0358 0.8466 -0.0611 H 0 0 0 0 0 0 0 0 0 0 0 0
-7.3537 -2.1613 -0.8341 H 0 0 0 0 0 0 0 0 0 0 0 0
-4.8918 -2.3170 -0.6722 H 0 0 0 0 0 0 0 0 0 0 0 0
-2.7151 0.6460 0.4109 H 0 0 0 0 0 0 0 0 0 0 0 0
-2.7119 -2.2966 -0.4289 H 0 0 0 0 0 0 0 0 0 0 0 0
-0.4422 -0.0064 0.3496 O 0 0 0 0 0 0 0 0 0 0 0 0
0.4903 -0.0588 0.5948 H 0 0 0 0 0 0 0 0 0 0 0 0
-0.5935 -3.2887 -0.3148 H 0 0 0 0 0 0 0 0 0 0 0 0
1.8962 -3.5130 0.1674 O 0 0 0 0 0 0 0 0 0 0 0 0
1.7326 -0.3849 -0.8652 H 0 0 0 0 0 0 0 0 0 0 0 0
3.8632 -2.0942 0.4892 H 0 0 0 0 0 0 0 0 0 0 0 0
3.2221 1.1909 -1.3727 H 0 0 0 0 0 0 0 0 0 0 0 0
4.9685 2.9226 -1.6560 H 0 0 0 0 0 0 0 0 0 0 0 0
7.3906 2.7955 -0.7261 O 0 0 0 0 0 0 0 0 0 0 0 0
8.1932 2.4819 -0.2864 H 0 0 0 0 0 0 0 0 0 0 0 0
8.3674 -1.4346 0.7416 H 0 0 0 0 0 0 0 0 0 0 0 0
9.4767 -0.3577 1.6290 H 0 0 0 0 0 0 0 0 0 0 0 0
7.8403 -0.6614 2.2665 H 0 0 0 0 0 0 0 0 0 0 0 0
1 46 1 0 0 0 0
1 47 1 0 0 0 0
2 1 1 0 0 0 0
3 8 1 0 0 0 0
3 2 1 0 0 0 0
4 3 2 0 0 0 0
5 6 2 0 0 0 0
5 4 1 0 0 0 0
6 7 1 0 0 0 0
7 10 1 0 0 0 0
7 8 2 0 0 0 0
8 9 1 0 0 0 0
10 40 1 0 0 0 0
11 12 1 0 0 0 0
11 10 2 0 0 0 0
12 38 2 0 0 0 0
13 12 1 0 0 0 0
13 14 2 0 0 0 0
14 35 1 0 0 0 0
15 14 1 0 0 0 0
15 16 2 0 0 0 0
16 33 1 0 0 0 0
17 16 1 0 0 0 0

17 22 2 0 0 0 0
18 17 1 0 0 0 0
19 18 2 0 0 0 0
19 20 1 0 0 0 0
20 21 2 0 0 0 0
21 22 1 0 0 0 0
21 24 1 0 0 0 0
22 23 1 0 0 0 0
24 25 1 0 0 0 0
25 26 1 0 0 0 0
25 27 1 0 0 0 0
28 25 1 0 0 0 0
29 20 1 0 0 0 0
29 30 1 0 0 0 0
31 19 1 0 0 0 0
32 18 1 0 0 0 0
34 15 1 0 0 0 0
35 36 1 0 0 0 0
37 13 1 0 0 0 0
39 11 1 0 0 0 0
41 6 1 0 0 0 0
42 5 1 0 0 0 0
43 4 1 0 0 0 0
43 44 1 0 0 0 0
45 1 1 0 0 0 0
M END

1B:DM-Curc S_0

53 54 0 0 0 0 0 0 0 0999 V2000
9.2180 1.4284 -0.9729 C 0 0 0 0 0 0 0 0 0 0 0 0
8.4339 1.2354 0.2141 O 0 0 0 0 0 0 0 0 0 0 0 0
7.3838 0.3573 0.0765 C 0 0 0 0 0 0 0 0 0 0 0 0
7.5970 -1.0248 -0.0617 C 0 0 0 0 0 0 0 0 0 0 0 0
6.4955 -1.8725 -0.1968 C 0 0 0 0 0 0 0 0 0 0 0 0
5.2032 -1.3717 -0.1722 C 0 0 0 0 0 0 0 0 0 0 0 0
4.9694 0.0043 -0.0006 C 0 0 0 0 0 0 0 0 0 0 0 0
6.0850 0.8499 0.1149 C 0 0 0 0 0 0 0 0 0 0 0 0
5.9528 1.9192 0.2364 H 0 0 0 0 0 0 0 0 0 0 0 0
3.6370 0.5969 0.0518 C 0 0 0 0 0 0 0 0 0 0 0 0
2.4531 -0.0379 -0.0531 C 0 0 0 0 0 0 0 0 0 0 0 0
1.1790 0.6542 0.0175 C 0 0 0 0 0 0 0 0 0 0 0 0
-0.0237 -0.0151 -0.0880 C 0 0 0 0 0 0 0 0 0 0 0 0
-1.2714 0.6997 -0.0088 C 0 0 0 0 0 0 0 0 0 0 0 0
-2.5189 -0.0757 -0.1229 C 0 0 0 0 0 0 0 0 0 0 0 0
-3.7207 0.5240 -0.0437 C 0 0 0 0 0 0 0 0 0 0 0 0
-5.0410 -0.0890 -0.1314 C 0 0 0 0 0 0 0 0 0 0 0 0
-5.2473 -1.4552 -0.3668 C 0 0 0 0 0 0 0 0 0 0 0 0
-6.5357 -1.9677 -0.4241 C 0 0 0 0 0 0 0 0 0 0 0 0
-7.6519 -1.1496 -0.2629 C 0 0 0 0 0 0 0 0 0 0 0 0

-7.4635 0.2337 -0.0483 C 0 0 0 0 0 0 0 0 0 0 0 0
-6.1691 0.7399 0.0223 C 0 0 0 0 0 0 0 0 0 0 0 0
-6.0089 1.7971 0.1882 H 0 0 0 0 0 0 0 0 0 0 0 0
-8.5949 0.9882 0.0596 O 0 0 0 0 0 0 0 0 0 0 0 0
-8.4592 2.3949 0.2199 C 0 0 0 0 0 0 0 0 0 0 0 0
-9.4739 2.7874 0.2562 H 0 0 0 0 0 0 0 0 0 0 0 0
-7.9399 2.6454 1.1513 H 0 0 0 0 0 0 0 0 0 0 0 0
-7.9276 2.8430 -0.6262 H 0 0 0 0 0 0 0 0 0 0 0 0
-8.8899 -1.7122 -0.3944 O 0 0 0 0 0 0 0 0 0 0 0 0
-9.7693 -1.6345 0.7356 C 0 0 0 0 0 0 0 0 0 0 0 0
-10.6500 -2.2159 0.4651 H 0 0 0 0 0 0 0 0 0 0 0 0
-9.2985 -2.0797 1.6190 H 0 0 0 0 0 0 0 0 0 0 0 0
-10.0561 -0.6042 0.9484 H 0 0 0 0 0 0 0 0 0 0 0 0
-6.7126 -3.0210 -0.6066 H 0 0 0 0 0 0 0 0 0 0 0 0
-4.4051 -2.1225 -0.5002 H 0 0 0 0 0 0 0 0 0 0 0 0
-3.7002 1.6005 0.1092 H 0 0 0 0 0 0 0 0 0 0 0 0
-2.4203 -1.1461 -0.2685 H 0 0 0 0 0 0 0 0 0 0 0 0
-1.2997 1.9451 0.1550 O 0 0 0 0 0 0 0 0 0 0 0 0
-0.0212 -1.0872 -0.2285 H 0 0 0 0 0 0 0 0 0 0 0 0
1.2221 1.9699 0.1910 O 0 0 0 0 0 0 0 0 0 0 0 0
0.2590 2.2677 0.2169 H 0 0 0 0 0 0 0 0 0 0 0 0
2.3937 -1.1108 -0.1959 H 0 0 0 0 0 0 0 0 0 0 0 0
3.6049 1.6731 0.1927 H 0 0 0 0 0 0 0 0 0 0 0 0
4.3719 -2.0583 -0.2745 H 0 0 0 0 0 0 0 0 0 0 0 0
6.6838 -2.9328 -0.3183 H 0 0 0 0 0 0 0 0 0 0 0 0
8.8578 -1.5630 -0.1138 O 0 0 0 0 0 0 0 0 0 0 0 0
9.6161 -1.4774 1.1038 C 0 0 0 0 0 0 0 0 0 0 0 0
9.1125 -2.0285 1.9047 H 0 0 0 0 0 0 0 0 0 0 0 0
10.5775 -1.9442 0.8925 H 0 0 0 0 0 0 0 0 0 0 0 0
9.7629 -0.4388 1.4073 H 0 0 0 0 0 0 0 0 0 0 0 0
9.6710 0.4910 -1.3048 H 0 0 0 0 0 0 0 0 0 0 0 0
8.6007 1.8438 -1.7763 H 0 0 0 0 0 0 0 0 0 0 0 0
9.9962 2.1436 -0.7088 H 0 0 0 0 0 0 0 0 0 0 0 0
1 53 1 0 0 0 0
1 2 1 0 0 0 0
3 8 1 0 0 0 0
3 2 1 0 0 0 0
4 3 2 0 0 0 0
5 6 2 0 0 0 0
5 4 1 0 0 0 0
6 7 1 0 0 0 0
7 10 1 0 0 0 0
7 8 2 0 0 0 0
8 9 1 0 0 0 0
10 43 1 0 0 0 0
11 12 1 0 0 0 0
11 10 2 0 0 0 0
12 40 1 0 0 0 0
13 14 1 0 0 0 0
13 12 2 0 0 0 0

```
14 38 2 0 0 0 0
15 16 2 0 0 0 0
15 14 1 0 0 0 0
16 36 1 0 0 0 0
17 16 1 0 0 0 0
17 22 2 0 0 0 0
18 17 1 0 0 0 0
19 18 2 0 0 0 0
19 20 1 0 0 0 0
20 21 2 0 0 0 0
21 22 1 0 0 0 0
21 24 1 0 0 0 0
22 23 1 0 0 0 0
24 25 1 0 0 0 0
25 26 1 0 0 0 0
25 27 1 0 0 0 0
28 25 1 0 0 0 0
29 20 1 0 0 0 0
29 30 1 0 0 0 0
30 33 1 0 0 0 0
30 32 1 0 0 0 0
31 30 1 0 0 0 0
34 19 1 0 0 0 0
35 18 1 0 0 0 0
37 15 1 0 0 0 0
39 13 1 0 0 0 0
40 41 1 0 0 0 0
42 11 1 0 0 0 0
44 6 1 0 0 0 0
45 5 1 0 0 0 0
46 4 1 0 0 0 0
46 47 1 0 0 0 0
47 50 1 0 0 0 0
47 48 1 0 0 0 0
49 47 1 0 0 0 0
51 1 1 0 0 0 0
52 1 1 0 0 0 0
M END
```

2B:BDeM-Curc S_0

```
39 40 0 0 0 0 0 0 0 0999 V2000
-5.2485 -1.2645 -0.0008 C 0 0 0 0 0 0 0 0 0 0 0 0
-6.5315 -1.7799 -0.0005 C 0 0 0 0 0 0 0 0 0 0 0 0
-7.6331 -0.9151 0.0004 C 0 0 0 0 0 0 0 0 0 0 0 0
-7.4277 0.4656 0.0010 C 0 0 0 0 0 0 0 0 0 0 0 0
-6.1335 0.9702 0.0007 C 0 0 0 0 0 0 0 0 0 0 0 0
-5.0127 0.1248 -0.0001 C 0 0 0 0 0 0 0 0 0 0 0 0
-3.6826 0.7178 -0.0003 C 0 0 0 0 0 0 0 0 0 0 0 0
-2.4895 0.0948 -0.0004 C 0 0 0 0 0 0 0 0 0 0 0 0
```

```
-1.2316  0.8614 -0.0006 C  0 0 0 0 0 0 0 0 0 0 0 0
 0.0068  0.1276 -0.0004 C  0 0 0 0 0 0 0 0 0 0 0 0
 1.2181  0.7915 -0.0003 C  0 0 0 0 0 0 0 0 0 0 0 0
 2.4850  0.0846 -0.0002 C  0 0 0 0 0 0 0 0 0 0 0 0
 3.6747  0.7193 -0.0002 C  0 0 0 0 0 0 0 0 0 0 0 0
 5.0039  0.1244 -0.0000 C  0 0 0 0 0 0 0 0 0 0 0 0
 6.1259  0.9686  0.0004 C  0 0 0 0 0 0 0 0 0 0 0 0
 7.4195  0.4629  0.0006 C  0 0 0 0 0 0 0 0 0 0 0 0
 7.6242 -0.9178  0.0004 C  0 0 0 0 0 0 0 0 0 0 0 0
 6.5218 -1.7815 -0.0001 C  0 0 0 0 0 0 0 0 0 0 0 0
 5.2391 -1.2652 -0.0003 C  0 0 0 0 0 0 0 0 0 0 0 0
 4.4030 -1.9544 -0.0006 H  0 0 0 0 0 0 0 0 0 0 0 0
 6.6986 -2.8500 -0.0002 H  0 0 0 0 0 0 0 0 0 0 0 0
 8.8624 -1.4840  0.0005 O  0 0 0 0 0 0 0 0 0 0 0 0
 9.5317 -0.7914  0.0009 H  0 0 0 0 0 0 0 0 0 0 0 0
 8.2691  1.1395  0.0009 H  0 0 0 0 0 0 0 0 0 0 0 0
 5.9775  2.0430  0.0005 H  0 0 0 0 0 0 0 0 0 0 0 0
 3.6465  1.8051 -0.0003 H  0 0 0 0 0 0 0 0 0 0 0 0
 2.4163 -0.9973 -0.0001 H  0 0 0 0 0 0 0 0 0 0 0 0
 1.2734  2.1184 -0.0003 O  0 0 0 0 0 0 0 0 0 0 0 0
 0.3117  2.4251 -0.0004 H  0 0 0 0 0 0 0 0 0 0 0 0
-0.0032 -0.9535 -0.0003 H  0 0 0 0 0 0 0 0 0 0 0 0
-1.2450  2.1181 -0.0004 O  0 0 0 0 0 0 0 0 0 0 0 0
-2.4055 -0.9868 -0.0003 H  0 0 0 0 0 0 0 0 0 0 0 0
-3.6452  1.8048 -0.0003 H  0 0 0 0 0 0 0 0 0 0 0 0
-5.9832  2.0443  0.0012 H  0 0 0 0 0 0 0 0 0 0 0 0
-8.2770  1.1426  0.0017 H  0 0 0 0 0 0 0 0 0 0 0 0
-8.8719 -1.4800  0.0006 O  0 0 0 0 0 0 0 0 0 0 0 0
-9.5402 -0.7865  0.0010 H  0 0 0 0 0 0 0 0 0 0 0 0
-6.7096 -2.8483 -0.0011 H  0 0 0 0 0 0 0 0 0 0 0 0
-4.4117 -1.9527 -0.0015 H  0 0 0 0 0 0 0 0 0 0 0 0
1 2 2 0 0 0 0
1 6 1 0 0 0 0
2 3 1 0 0 0 0
3 36 1 0 0 0 0
3 4 2 0 0 0 0
4 35 1 0 0 0 0
5 4 1 0 0 0 0
5 34 1 0 0 0 0
6 5 2 0 0 0 0
7 33 1 0 0 0 0
7 6 1 0 0 0 0
8 32 1 0 0 0 0
8 7 2 0 0 0 0
9 10 1 0 0 0 0
9 31 2 0 0 0 0
9 8 1 0 0 0 0
10 11 2 0 0 0 0
10 30 1 0 0 0 0
11 28 1 0 0 0 0
```

```
11 12 1 0 0 0 0
12 13 2 0 0 0 0
12 27 1 0 0 0 0
13 14 1 0 0 0 0
14 15 2 0 0 0 0
15 25 1 0 0 0 0
15 16 1 0 0 0 0
16 24 1 0 0 0 0
17 22 1 0 0 0 0
17 16 2 0 0 0 0
18 17 1 0 0 0 0
19 18 2 0 0 0 0
19 14 1 0 0 0 0
20 19 1 0 0 0 0
21 18 1 0 0 0 0
22 23 1 0 0 0 0
26 13 1 0 0 0 0
29 28 1 0 0 0 0
36 37 1 0 0 0 0
38 2 1 0 0 0 0
39 1 1 0 0 0 0
M END
```

3B: CY S$_0$

```
49 51 0 0 0 0 0 0 0 0999 V2000
  1.2574   0.8426   0.6088 C  0 0 0 0 0 0 0 0 0 0 0 0
 -0.0002   1.1189   1.4339 C  0 0 0 0 0 0 0 0 0 0 0 0
 -0.0002   2.1570   1.7804 H  0 0 0 0 0 0 0 0 0 0 0 0
 -1.2575   0.8427   0.6084 C  0 0 0 0 0 0 0 0 0 0 0 0
 -1.2948  -0.5801   0.0960 C  0 0 0 0 0 0 0 0 0 0 0 0
 -2.4196  -1.2882  -0.1598 C  0 0 0 0 0 0 0 0 0 0 0 0
 -2.2350  -2.3230  -0.4366 H  0 0 0 0 0 0 0 0 0 0 0 0
 -3.8300  -0.9223  -0.1036 C  0 0 0 0 0 0 0 0 0 0 0 0
 -4.7696  -1.9599   0.0158 C  0 0 0 0 0 0 0 0 0 0 0 0
 -6.1346  -1.7058   0.0797 C  0 0 0 0 0 0 0 0 0 0 0 0
 -6.6022  -0.4011  -0.0011 C  0 0 0 0 0 0 0 0 0 0 0 0
 -5.6795   0.6536  -0.1537 C  0 0 0 0 0 0 0 0 0 0 0 0
 -4.3187   0.4009  -0.2011 C  0 0 0 0 0 0 0 0 0 0 0 0
 -3.6315   1.2176  -0.3556 H  0 0 0 0 0 0 0 0 0 0 0 0
 -6.2763   1.8874  -0.2528 O  0 0 0 0 0 0 0 0 0 0 0 0
 -5.4503   3.0323  -0.4190 C  0 0 0 0 0 0 0 0 0 0 0 0
 -4.7671   3.1537   0.4283 H  0 0 0 0 0 0 0 0 0 0 0 0
 -4.8743   2.9737  -1.3485 H  0 0 0 0 0 0 0 0 0 0 0 0
 -6.1248   3.8855  -0.4650 H  0 0 0 0 0 0 0 0 0 0 0 0
 -7.9327  -0.1423   0.0487 O  0 0 0 0 0 0 0 0 0 0 0 0
 -8.0417   0.8144  -0.0410 H  0 0 0 0 0 0 0 0 0 0 0 0
 -6.8544  -2.5087   0.1824 H  0 0 0 0 0 0 0 0 0 0 0 0
 -4.4169  -2.9834   0.0682 H  0 0 0 0 0 0 0 0 0 0 0 0
 -0.0000  -1.3163  -0.1200 C  0 0 0 0 0 0 0 0 0 0 0 0
```

```
 1.2947  -0.5800   0.0960 C  0 0 0 0 0 0 0 0 0 0 0 0 0
 2.4195  -1.2881  -0.1602 C  0 0 0 0 0 0 0 0 0 0 0 0 0
 3.8300  -0.9222  -0.1041 C  0 0 0 0 0 0 0 0 0 0 0 0 0
 4.7695  -1.9599   0.0139 C  0 0 0 0 0 0 0 0 0 0 0 0 0
 6.1345  -1.7061   0.0775 C  0 0 0 0 0 0 0 0 0 0 0 0 0
 6.6022  -0.4013  -0.0020 C  0 0 0 0 0 0 0 0 0 0 0 0 0
 5.6795   0.6536  -0.1533 C  0 0 0 0 0 0 0 0 0 0 0 0 0
 4.3188   0.4011  -0.2004 C  0 0 0 0 0 0 0 0 0 0 0 0 0
 3.6315   1.2181  -0.3538 H  0 0 0 0 0 0 0 0 0 0 0 0 0
 6.2764   1.8875  -0.2512 O  0 0 0 0 0 0 0 0 0 0 0 0 0
 5.4504   3.0327  -0.4154 C  0 0 0 0 0 0 0 0 0 0 0 0 0
 4.8739   2.9754  -1.3448 H  0 0 0 0 0 0 0 0 0 0 0 0 0
 4.7677   3.1529   0.4324 H  0 0 0 0 0 0 0 0 0 0 0 0 0
 6.1250   3.8859  -0.4606 H  0 0 0 0 0 0 0 0 0 0 0 0 0
 7.9328  -0.1426   0.0475 O  0 0 0 0 0 0 0 0 0 0 0 0 0
 8.0418   0.8142  -0.0411 H  0 0 0 0 0 0 0 0 0 0 0 0 0
 6.8543  -2.5091   0.1791 H  0 0 0 0 0 0 0 0 0 0 0 0 0
 4.4168  -2.9834   0.0654 H  0 0 0 0 0 0 0 0 0 0 0 0 0
 2.2349  -2.3227  -0.4373 H  0 0 0 0 0 0 0 0 0 0 0 0 0
-0.0000  -2.4860  -0.4866 O  0 0 0 0 0 0 0 0 0 0 0 0 0
-1.2849   1.5533  -0.2317 H  0 0 0 0 0 0 0 0 0 0 0 0 0
-2.1487   1.0397   1.2102 H  0 0 0 0 0 0 0 0 0 0 0 0 0
-0.0004   0.4836   2.3269 H  0 0 0 0 0 0 0 0 0 0 0 0 0
 2.1484   1.0392   1.2111 H  0 0 0 0 0 0 0 0 0 0 0 0 0
 1.2853   1.5534  -0.2310 H  0 0 0 0 0 0 0 0 0 0 0 0 0
1 48  1  0  0  0  0
1  2  1  0  0  0  0
2  3  1  0  0  0  0
2 47  1  0  0  0  0
4 46  1  0  0  0  0
4  2  1  0  0  0  0
5  4  1  0  0  0  0
6  8  1  0  0  0  0
6  5  2  0  0  0  0
7  6  1  0  0  0  0
8  9  2  0  0  0  0
9 23  1  0  0  0  0
9 10  1  0  0  0  0
10 22  1  0  0  0  0
11 20  1  0  0  0  0
11 10  2  0  0  0  0
12 11  1  0  0  0  0
13 12  2  0  0  0  0
13  8  1  0  0  0  0
14 13  1  0  0  0  0
15 12  1  0  0  0  0
16 15  1  0  0  0  0
16 17  1  0  0  0  0
18 16  1  0  0  0  0
19 16  1  0  0  0  0
```

21 20 1 0 0 0 0
24 5 1 0 0 0 0
24 25 1 0 0 0 0
25 1 1 0 0 0 0
26 27 1 0 0 0 0
26 25 2 0 0 0 0
27 28 2 0 0 0 0
28 42 1 0 0 0 0
28 29 1 0 0 0 0
29 41 1 0 0 0 0
30 39 1 0 0 0 0
30 29 2 0 0 0 0
31 30 1 0 0 0 0
32 31 2 0 0 0 0
32 27 1 0 0 0 0
33 32 1 0 0 0 0
34 31 1 0 0 0 0
35 34 1 0 0 0 0
35 37 1 0 0 0 0
36 35 1 0 0 0 0
38 35 1 0 0 0 0
40 39 1 0 0 0 0
43 26 1 0 0 0 0
44 24 2 0 0 0 0
45 4 1 0 0 0 0
49 1 1 0 0 0 0
M END

3B': O-CY S_0

49 51 0 0 0 0 0 0 0 0999 V2000
1.2573 0.8500 0.6012 C 0 0 0 0 0 0 0 0 0 0 0 0
-0.0003 1.1258 1.4263 C 0 0 0 0 0 0 0 0 0 0 0 0
-0.0004 2.1638 1.7729 H 0 0 0 0 0 0 0 0 0 0 0 0
-1.2576 0.8500 0.6007 C 0 0 0 0 0 0 0 0 0 0 0 0
-1.2951 -0.5726 0.0880 C 0 0 0 0 0 0 0 0 0 0 0 0
-2.4192 -1.2812 -0.1680 C 0 0 0 0 0 0 0 0 0 0 0 0
-2.2341 -2.3154 -0.4463 H 0 0 0 0 0 0 0 0 0 0 0 0
-3.8305 -0.9165 -0.1087 C 0 0 0 0 0 0 0 0 0 0 0 0
-4.7659 -1.9531 0.0133 C 0 0 0 0 0 0 0 0 0 0 0 0
-6.1288 -1.6846 0.0828 C 0 0 0 0 0 0 0 0 0 0 0 0
-6.6031 -0.3805 0.0057 C 0 0 0 0 0 0 0 0 0 0 0 0
-5.6831 0.6804 -0.1530 C 0 0 0 0 0 0 0 0 0 0 0 0
-4.3228 0.4050 -0.2039 C 0 0 0 0 0 0 0 0 0 0 0 0
-3.6320 1.2174 -0.3615 H 0 0 0 0 0 0 0 0 0 0 0 0
-6.2375 1.9190 -0.2524 O 0 0 0 0 0 0 0 0 0 0 0 0
-5.3739 3.0322 -0.4217 C 0 0 0 0 0 0 0 0 0 0 0 0
-4.6832 3.1383 0.4228 H 0 0 0 0 0 0 0 0 0 0 0 0
-4.8003 2.9596 -1.3528 H 0 0 0 0 0 0 0 0 0 0 0 0
-6.0232 3.9049 -0.4670 H 0 0 0 0 0 0 0 0 0 0 0 0

-7.9233 -0.0572 0.0582 O 0 0 0 0 0 0 0 0 0 0 0 0
-8.4361 -0.8661 0.1587 H 0 0 0 0 0 0 0 0 0 0 0 0
-6.8388 -2.4999 0.1900 H 0 0 0 0 0 0 0 0 0 0 0 0
-4.4197 -2.9786 0.0650 H 0 0 0 0 0 0 0 0 0 0 0 0
-0.0000 -1.3090 -0.1280 C 0 0 0 0 0 0 0 0 0 0 0 0
1.2949 -0.5724 0.0880 C 0 0 0 0 0 0 0 0 0 0 0 0
2.4191 -1.2808 -0.1686 C 0 0 0 0 0 0 0 0 0 0 0 0
3.8304 -0.9163 -0.1096 C 0 0 0 0 0 0 0 0 0 0 0 0
4.7658 -1.9532 0.0102 C 0 0 0 0 0 0 0 0 0 0 0 0
6.1287 -1.6851 0.0792 C 0 0 0 0 0 0 0 0 0 0 0 0
6.6032 -0.3809 0.0041 C 0 0 0 0 0 0 0 0 0 0 0 0
5.6832 0.6805 -0.1521 C 0 0 0 0 0 0 0 0 0 0 0 0
4.3229 0.4054 -0.2028 C 0 0 0 0 0 0 0 0 0 0 0 0
3.6321 1.2182 -0.3584 H 0 0 0 0 0 0 0 0 0 0 0 0
6.2378 1.9191 -0.2497 O 0 0 0 0 0 0 0 0 0 0 0 0
5.3743 3.0329 -0.4156 C 0 0 0 0 0 0 0 0 0 0 0 0
4.7998 2.9626 -1.3464 H 0 0 0 0 0 0 0 0 0 0 0 0
4.6843 3.1371 0.4298 H 0 0 0 0 0 0 0 0 0 0 0 0
6.0236 3.9055 -0.4595 H 0 0 0 0 0 0 0 0 0 0 0 0
7.9235 -0.0578 0.0565 O 0 0 0 0 0 0 0 0 0 0 0 0
8.4362 -0.8670 0.1546 H 0 0 0 0 0 0 0 0 0 0 0 0
6.8386 -2.5006 0.1847 H 0 0 0 0 0 0 0 0 0 0 0 0
4.4194 -2.9787 0.0603 H 0 0 0 0 0 0 0 0 0 0 0 0
2.2339 -2.3149 -0.4474 H 0 0 0 0 0 0 0 0 0 0 0 0
0.0000 -2.4791 -0.4937 O 0 0 0 0 0 0 0 0 0 0 0 0
-1.2845 1.5601 -0.2396 H 0 0 0 0 0 0 0 0 0 0 0 0
-2.1491 1.0475 1.2016 H 0 0 0 0 0 0 0 0 0 0 0 0
-0.0005 0.4905 2.3194 H 0 0 0 0 0 0 0 0 0 0 0 0
2.1484 1.0473 1.2025 H 0 0 0 0 0 0 0 0 0 0 0 0
1.2846 1.5602 -0.2389 H 0 0 0 0 0 0 0 0 0 0 0 0
1 48 1 0 0 0 0
1 2 1 0 0 0 0
2 3 1 0 0 0 0
2 47 1 0 0 0 0
4 46 1 0 0 0 0
4 2 1 0 0 0 0
5 4 1 0 0 0 0
6 8 1 0 0 0 0
6 5 2 0 0 0 0
7 6 1 0 0 0 0
8 9 2 0 0 0 0
9 23 1 0 0 0 0
9 10 1 0 0 0 0
10 22 1 0 0 0 0
11 20 1 0 0 0 0
11 10 2 0 0 0 0
12 11 1 0 0 0 0
13 12 2 0 0 0 0
13 8 1 0 0 0 0
14 13 1 0 0 0 0

```
15 12  1  0  0  0  0
16 15  1  0  0  0  0
16 17  1  0  0  0  0
18 16  1  0  0  0  0
19 16  1  0  0  0  0
20 21  1  0  0  0  0
24  5  1  0  0  0  0
24 25  1  0  0  0  0
25  1  1  0  0  0  0
26 27  1  0  0  0  0
26 25  2  0  0  0  0
27 28  2  0  0  0  0
28 42  1  0  0  0  0
28 29  1  0  0  0  0
29 41  1  0  0  0  0
30 39  1  0  0  0  0
30 29  2  0  0  0  0
31 30  1  0  0  0  0
32 31  2  0  0  0  0
32 27  1  0  0  0  0
33 32  1  0  0  0  0
34 31  1  0  0  0  0
35 34  1  0  0  0  0
35 37  1  0  0  0  0
36 35  1  0  0  0  0
38 35  1  0  0  0  0
39 40  1  0  0  0  0
43 26  1  0  0  0  0
44 24  2  0  0  0  0
45  4  1  0  0  0  0
49  1  1  0  0  0  0
M  END
```

1A: CCE-Curc S_1

```
47 48  0  0  0  0  0  0  0  0999 V2000
    8.5487   -2.2371    0.0001 C   0  0  0  0  0  0  0  0  0  0  0  0
    8.5891   -0.8053    0.0002 O   0  0  0  0  0  0  0  0  0  0  0  0
    7.4220   -0.1175    0.0001 C   0  0  0  0  0  0  0  0  0  0  0  0
    7.6107    1.2999    0.0002 C   0  0  0  0  0  0  0  0  0  0  0  0
    6.4952    2.1489    0.0001 C   0  0  0  0  0  0  0  0  0  0  0  0
    5.2253    1.6091    0.0000 C   0  0  0  0  0  0  0  0  0  0  0  0
    5.0124    0.1950   -0.0000 C   0  0  0  0  0  0  0  0  0  0  0  0
    6.1438   -0.6507    0.0000 C   0  0  0  0  0  0  0  0  0  0  0  0
    5.9918   -1.7231   -0.0000 H   0  0  0  0  0  0  0  0  0  0  0  0
    3.6940   -0.4098   -0.0001 C   0  0  0  0  0  0  0  0  0  0  0  0
    2.5058    0.2396   -0.0002 C   0  0  0  0  0  0  0  0  0  0  0  0
    1.2134   -0.5251   -0.0003 C   0  0  0  0  0  0  0  0  0  0  0  0
    0.0289    0.2030   -0.0003 C   0  0  0  0  0  0  0  0  0  0  0  0
   -1.2418   -0.4691   -0.0002 C   0  0  0  0  0  0  0  0  0  0  0  0
```

-2.4725 0.2075 -0.0001 C 0 0 0 0 0 0 0 0 0 0 0 0
-3.7094 -0.4325 -0.0001 C 0 0 0 0 0 0 0 0 0 0 0 0
-5.0009 0.1799 -0.0000 C 0 0 0 0 0 0 0 0 0 0 0 0
-5.2121 1.5855 -0.0000 C 0 0 0 0 0 0 0 0 0 0 0 0
-6.4904 2.1275 0.0001 C 0 0 0 0 0 0 0 0 0 0 0 0
-7.6128 1.2993 0.0001 C 0 0 0 0 0 0 0 0 0 0 0 0
-7.4314 -0.1018 0.0001 C 0 0 0 0 0 0 0 0 0 0 0 0
-6.1620 -0.6496 0.0000 C 0 0 0 0 0 0 0 0 0 0 0 0
-6.0268 -1.7255 -0.0000 H 0 0 0 0 0 0 0 0 0 0 0 0
-8.6189 -0.8046 0.0001 O 0 0 0 0 0 0 0 0 0 0 0 0
-8.5533 -2.2208 0.0001 C 0 0 0 0 0 0 0 0 0 0 0 0
-9.5853 -2.5755 0.0001 H 0 0 0 0 0 0 0 0 0 0 0 0
-8.0392 -2.5974 -0.8930 H 0 0 0 0 0 0 0 0 0 0 0 0
-8.0391 -2.5975 0.8931 H 0 0 0 0 0 0 0 0 0 0 0 0
-8.8701 1.8323 0.0002 O 0 0 0 0 0 0 0 0 0 0 0 0
-9.4863 1.0833 0.0002 H 0 0 0 0 0 0 0 0 0 0 0 0
-6.6440 3.2022 0.0001 H 0 0 0 0 0 0 0 0 0 0 0 0
-4.3597 2.2560 -0.0000 H 0 0 0 0 0 0 0 0 0 0 0 0
-3.6903 -1.5193 -0.0001 H 0 0 0 0 0 0 0 0 0 0 0 0
-2.4134 1.2916 -0.0001 H 0 0 0 0 0 0 0 0 0 0 0 0
-1.2300 -1.8067 -0.0001 O 0 0 0 0 0 0 0 0 0 0 0 0
-0.2524 -2.0781 -0.0001 H 0 0 0 0 0 0 0 0 0 0 0 0
0.0449 1.2865 -0.0002 H 0 0 0 0 0 0 0 0 0 0 0 0
1.3007 -1.8035 -0.0002 O 0 0 0 0 0 0 0 0 0 0 0 0
2.4429 1.3256 -0.0001 H 0 0 0 0 0 0 0 0 0 0 0 0
3.6359 -1.4957 -0.0002 H 0 0 0 0 0 0 0 0 0 0 0 0
4.3702 2.2740 -0.0000 H 0 0 0 0 0 0 0 0 0 0 0 0
6.6604 3.2203 0.0002 H 0 0 0 0 0 0 0 0 0 0 0 0
8.8486 1.8117 0.0003 O 0 0 0 0 0 0 0 0 0 0 0 0
9.4826 1.0735 0.0003 H 0 0 0 0 0 0 0 0 0 0 0 0
8.0420 -2.6082 -0.8961 H 0 0 0 0 0 0 0 0 0 0 0 0
9.5878 -2.5633 0.0002 H 0 0 0 0 0 0 0 0 0 0 0 0
8.0418 -2.6082 0.8962 H 0 0 0 0 0 0 0 0 0 0 0 0
1 46 1 0 0 0 0
1 2 1 0 0 0 0
1 47 1 0 0 0 0
3 2 1 0 0 0 0
3 4 2 0 0 0 0
4 43 1 0 0 0 0
5 4 1 0 0 0 0
5 42 1 0 0 0 0
6 5 2 0 0 0 0
7 8 2 0 0 0 0
7 6 1 0 0 0 0
8 3 1 0 0 0 0
9 8 1 0 0 0 0
10 7 1 0 0 0 0
11 39 1 0 0 0 0
11 10 2 0 0 0 0
12 13 1 0 0 0 0

12 38 2 0 0 0 0
12 11 1 0 0 0 0
13 37 1 0 0 0 0
13 14 1 0 0 0 0
14 15 2 0 0 0 0
14 35 1 0 0 0 0
15 34 1 0 0 0 0
15 16 1 0 0 0 0
16 17 1 0 0 0 0
17 18 2 0 0 0 0
17 22 1 0 0 0 0
18 19 1 0 0 0 0
19 31 1 0 0 0 0
19 20 2 0 0 0 0
20 29 1 0 0 0 0
21 20 1 0 0 0 0
21 24 1 0 0 0 0
22 21 2 0 0 0 0
23 22 1 0 0 0 0
25 24 1 0 0 0 0
25 26 1 0 0 0 0
25 28 1 0 0 0 0
27 25 1 0 0 0 0
29 30 1 0 0 0 0
32 18 1 0 0 0 0
33 16 1 0 0 0 0
35 36 1 0 0 0 0
40 10 1 0 0 0 0
41 6 1 0 0 0 0
43 44 1 0 0 0 0
45 1 1 0 0 0 0
M END

2A: CCE-Curc-Conf S_0

47 48 0 0 0 0 0 0 0 0999 V2000
-5.8461 3.4550 0.0013 C 0 0 0 0 0 0 0 0 0 0 0 0
-6.8772 2.4753 0.0005 O 0 0 0 0 0 0 0 0 0 0 0 0
-6.5235 1.1493 0.0001 C 0 0 0 0 0 0 0 0 0 0 0 0
-7.6357 0.2767 -0.0003 C 0 0 0 0 0 0 0 0 0 0 0 0
-7.4261 -1.0944 -0.0007 C 0 0 0 0 0 0 0 0 0 0 0 0
-6.1287 -1.5984 -0.0006 C 0 0 0 0 0 0 0 0 0 0 0 0
-5.0141 -0.7491 -0.0002 C 0 0 0 0 0 0 0 0 0 0 0 0
-5.2368 0.6463 0.0002 C 0 0 0 0 0 0 0 0 0 0 0 0
-4.3927 1.3220 0.0006 H 0 0 0 0 0 0 0 0 0 0 0 0
-3.6831 -1.3371 -0.0002 C 0 0 0 0 0 0 0 0 0 0 0 0
-2.4892 -0.7139 -0.0000 C 0 0 0 0 0 0 0 0 0 0 0 0
-1.2312 -1.4790 0.0000 C 0 0 0 0 0 0 0 0 0 0 0 0
0.0074 -0.7451 0.0001 C 0 0 0 0 0 0 0 0 0 0 0 0
1.2194 -1.4087 0.0001 C 0 0 0 0 0 0 0 0 0 0 0 0

2.4863 -0.7027 0.0001 C 0 0 0 0 0 0 0 0 0 0 0 0
3.6770 -1.3374 0.0002 C 0 0 0 0 0 0 0 0 0 0 0 0
5.0069 -0.7475 0.0002 C 0 0 0 0 0 0 0 0 0 0 0 0
5.2293 0.6482 -0.0002 C 0 0 0 0 0 0 0 0 0 0 0 0
6.5157 1.1519 -0.0002 C 0 0 0 0 0 0 0 0 0 0 0 0
7.6287 0.2802 0.0003 C 0 0 0 0 0 0 0 0 0 0 0 0
7.4195 -1.0910 0.0007 C 0 0 0 0 0 0 0 0 0 0 0 0
6.1226 -1.5958 0.0006 C 0 0 0 0 0 0 0 0 0 0 0 0
5.9696 -2.6689 0.0009 H 0 0 0 0 0 0 0 0 0 0 0 0
8.2813 -1.7469 0.0010 H 0 0 0 0 0 0 0 0 0 0 0 0
8.8862 0.7852 0.0003 O 0 0 0 0 0 0 0 0 0 0 0 0
8.8093 1.7494 -0.0000 H 0 0 0 0 0 0 0 0 0 0 0 0
6.8683 2.4779 -0.0006 O 0 0 0 0 0 0 0 0 0 0 0 0
5.8369 3.4574 -0.0013 C 0 0 0 0 0 0 0 0 0 0 0 0
5.2117 3.3719 -0.8962 H 0 0 0 0 0 0 0 0 0 0 0 0
5.2113 3.3727 0.8935 H 0 0 0 0 0 0 0 0 0 0 0 0
6.3395 4.4229 -0.0016 H 0 0 0 0 0 0 0 0 0 0 0 0
4.3861 1.3250 -0.0006 H 0 0 0 0 0 0 0 0 0 0 0 0
3.6488 -2.4230 0.0003 H 0 0 0 0 0 0 0 0 0 0 0 0
2.4164 0.3793 0.0001 H 0 0 0 0 0 0 0 0 0 0 0 0
1.2745 -2.7355 0.0001 O 0 0 0 0 0 0 0 0 0 0 0 0
0.3129 -3.0425 0.0001 H 0 0 0 0 0 0 0 0 0 0 0 0
-0.0028 0.3362 0.0001 H 0 0 0 0 0 0 0 0 0 0 0 0
-1.2436 -2.7361 -0.0001 O 0 0 0 0 0 0 0 0 0 0 0 0
-2.4041 0.3680 0.0001 H 0 0 0 0 0 0 0 0 0 0 0 0
-3.6457 -2.4238 -0.0003 H 0 0 0 0 0 0 0 0 0 0 0 0
-5.9740 -2.6712 -0.0010 H 0 0 0 0 0 0 0 0 0 0 0 0
-8.2877 -1.7507 -0.0011 H 0 0 0 0 0 0 0 0 0 0 0 0
-8.8936 0.7812 -0.0004 O 0 0 0 0 0 0 0 0 0 0 0 0
-8.8168 1.7454 -0.0000 H 0 0 0 0 0 0 0 0 0 0 0 0
-6.3489 4.4204 0.0017 H 0 0 0 0 0 0 0 0 0 0 0 0
-5.2209 3.3694 0.8962 H 0 0 0 0 0 0 0 0 0 0 0 0
-5.2203 3.3704 -0.8933 H 0 0 0 0 0 0 0 0 0 0 0 0
1 45 1 0 0 0 0
1 46 1 0 0 0 0
2 1 1 0 0 0 0
3 8 1 0 0 0 0
3 2 1 0 0 0 0
4 3 2 0 0 0 0
5 6 2 0 0 0 0
5 4 1 0 0 0 0
6 7 1 0 0 0 0
7 10 1 0 0 0 0
7 8 2 0 0 0 0
8 9 1 0 0 0 0
10 11 2 0 0 0 0
11 12 1 0 0 0 0
11 39 1 0 0 0 0
12 13 1 0 0 0 0
13 14 2 0 0 0 0

13 37 1 0 0 0 0
14 35 1 0 0 0 0
14 15 1 0 0 0 0
15 16 2 0 0 0 0
16 17 1 0 0 0 0
16 33 1 0 0 0 0
17 22 2 0 0 0 0
18 19 2 0 0 0 0
18 17 1 0 0 0 0
19 20 1 0 0 0 0
20 25 1 0 0 0 0
20 21 2 0 0 0 0
21 24 1 0 0 0 0
22 21 1 0 0 0 0
22 23 1 0 0 0 0
26 25 1 0 0 0 0
27 19 1 0 0 0 0
28 27 1 0 0 0 0
28 30 1 0 0 0 0
29 28 1 0 0 0 0
31 28 1 0 0 0 0
32 18 1 0 0 0 0
34 15 1 0 0 0 0
35 36 1 0 0 0 0
38 12 2 0 0 0 0
40 10 1 0 0 0 0
41 6 1 0 0 0 0
42 5 1 0 0 0 0
43 4 1 0 0 0 0
43 44 1 0 0 0 0
47 1 1 0 0 0 0
M END

2A: CCE-Curc-Conf S_1

47 48 0 0 0 0 0 0 0 0999 V2000
5.6899 3.4512 -0.0005 C 0 0 0 0 0 0 0 0 0 0 0 0
6.7612 2.5216 -0.0003 O 0 0 0 0 0 0 0 0 0 0 0 0
6.4566 1.1781 -0.0001 C 0 0 0 0 0 0 0 0 0 0 0 0
7.6049 0.3515 0.0000 C 0 0 0 0 0 0 0 0 0 0 0 0
7.4462 -1.0360 0.0002 C 0 0 0 0 0 0 0 0 0 0 0 0
6.1750 -1.5897 0.0002 C 0 0 0 0 0 0 0 0 0 0 0 0
5.0053 -0.7835 0.0001 C 0 0 0 0 0 0 0 0 0 0 0 0
5.1862 0.6323 -0.0001 C 0 0 0 0 0 0 0 0 0 0 0 0
4.3181 1.2792 -0.0002 H 0 0 0 0 0 0 0 0 0 0 0 0
3.7189 -1.4086 0.0002 C 0 0 0 0 0 0 0 0 0 0 0 0
2.4763 -0.7937 0.0001 C 0 0 0 0 0 0 0 0 0 0 0 0
1.2581 -1.5347 0.0002 C 0 0 0 0 0 0 0 0 0 0 0 0
-0.0164 -0.7985 0.0001 C 0 0 0 0 0 0 0 0 0 0 0 0
-1.2264 -1.4793 0.0000 C 0 0 0 0 0 0 0 0 0 0 0 0

```
-2.4787 -0.7685 -0.0000 C 0 0 0 0 0 0 0 0 0 0 0 0 0
-3.6979 -1.3950 -0.0001 C 0 0 0 0 0 0 0 0 0 0 0 0 0
-4.9924 -0.7640 -0.0001 C 0 0 0 0 0 0 0 0 0 0 0 0 0
-5.1731 0.6464 0.0001 C 0 0 0 0 0 0 0 0 0 0 0 0 0
-6.4445 1.1868 0.0001 C 0 0 0 0 0 0 0 0 0 0 0 0 0
-7.5908 0.3431 -0.0002 C 0 0 0 0 0 0 0 0 0 0 0 0 0
-7.4276 -1.0460 -0.0004 C 0 0 0 0 0 0 0 0 0 0 0 0 0
-6.1531 -1.5854 -0.0003 C 0 0 0 0 0 0 0 0 0 0 0 0 0
-6.0266 -2.6633 -0.0005 H 0 0 0 0 0 0 0 0 0 0 0 0 0
-8.3125 -1.6728 -0.0005 H 0 0 0 0 0 0 0 0 0 0 0 0 0
-8.8248 0.8821 -0.0002 O 0 0 0 0 0 0 0 0 0 0 0 0 0
-8.7240 1.8491 -0.0000 H 0 0 0 0 0 0 0 0 0 0 0 0 0
-6.7651 2.5142 0.0003 O 0 0 0 0 0 0 0 0 0 0 0 0 0
-5.7089 3.4716 0.0005 C 0 0 0 0 0 0 0 0 0 0 0 0 0
-5.0860 3.3680 0.8961 H 0 0 0 0 0 0 0 0 0 0 0 0 0
-5.0858 3.3684 -0.8949 H 0 0 0 0 0 0 0 0 0 0 0 0 0
-6.1893 4.4499 0.0007 H 0 0 0 0 0 0 0 0 0 0 0 0 0
-4.3088 1.2977 0.0003 H 0 0 0 0 0 0 0 0 0 0 0 0 0
-3.6857 -2.4814 -0.0001 H 0 0 0 0 0 0 0 0 0 0 0 0 0
-2.4034 0.3153 0.0000 H 0 0 0 0 0 0 0 0 0 0 0 0 0
-1.2702 -2.8127 -0.0000 O 0 0 0 0 0 0 0 0 0 0 0 0 0
-0.2687 -3.0832 -0.0000 H 0 0 0 0 0 0 0 0 0 0 0 0 0
-0.0180 0.2859 0.0001 H 0 0 0 0 0 0 0 0 0 0 0 0 0
1.2066 -2.8133 -0.0000 O 0 0 0 0 0 0 0 0 0 0 0 0 0
2.3898 0.2896 0.0001 H 0 0 0 0 0 0 0 0 0 0 0 0 0
3.7078 -2.4960 0.0002 H 0 0 0 0 0 0 0 0 0 0 0 0 0
6.0619 -2.6698 0.0004 H 0 0 0 0 0 0 0 0 0 0 0 0 0
8.3347 -1.6590 0.0003 H 0 0 0 0 0 0 0 0 0 0 0 0 0
8.8488 0.9041 0.0000 O 0 0 0 0 0 0 0 0 0 0 0 0 0
8.7276 1.8669 -0.0001 H 0 0 0 0 0 0 0 0 0 0 0 0 0
6.1454 4.4426 -0.0006 H 0 0 0 0 0 0 0 0 0 0 0 0 0
5.0637 3.3380 -0.8942 H 0 0 0 0 0 0 0 0 0 0 0 0 0
5.0636 3.3382 0.8932 H 0 0 0 0 0 0 0 0 0 0 0 0 0
1 2 1 0 0 0 0
1 47 1 0 0 0 0
2 3 1 0 0 0 0
3 8 2 0 0 0 0
3 4 1 0 0 0 0
4 5 2 0 0 0 0
5 6 1 0 0 0 0
5 42 1 0 0 0 0
6 41 1 0 0 0 0
7 10 1 0 0 0 0
7 6 2 0 0 0 0
8 7 1 0 0 0 0
9 8 1 0 0 0 0
10 40 1 0 0 0 0
11 10 2 0 0 0 0
11 12 1 0 0 0 0
13 12 1 0 0 0 0
```

14 13 2 0 0 0 0
15 34 1 0 0 0 0
15 14 1 0 0 0 0
16 15 2 0 0 0 0
17 16 1 0 0 0 0
17 18 2 0 0 0 0
18 32 1 0 0 0 0
19 18 1 0 0 0 0
19 27 1 0 0 0 0
20 19 2 0 0 0 0
21 22 2 0 0 0 0
21 20 1 0 0 0 0
22 17 1 0 0 0 0
23 22 1 0 0 0 0
24 21 1 0 0 0 0
25 20 1 0 0 0 0
25 26 1 0 0 0 0
27 28 1 0 0 0 0
28 31 1 0 0 0 0
28 29 1 0 0 0 0
30 28 1 0 0 0 0
33 16 1 0 0 0 0
35 14 1 0 0 0 0
36 35 1 0 0 0 0
37 13 1 0 0 0 0
38 12 2 0 0 0 0
39 11 1 0 0 0 0
43 4 1 0 0 0 0
44 43 1 0 0 0 0
45 1 1 0 0 0 0
46 1 1 0 0 0 0
M END

3B: CY S_1

49 51 0 0 0 0 0 0 0 0999 V2000
1.2610 0.8902 0.6198 C 0 0 0 0 0 0 0 0 0 0 0 0
-0.0000 1.2125 1.4245 C 0 0 0 0 0 0 0 0 0 0 0 0
-0.0000 2.2692 1.7155 H 0 0 0 0 0 0 0 0 0 0 0 0
-1.2610 0.8902 0.6197 C 0 0 0 0 0 0 0 0 0 0 0 0
-1.2683 -0.5416 0.1288 C 0 0 0 0 0 0 0 0 0 0 0 0
-2.4087 -1.2962 -0.1032 C 0 0 0 0 0 0 0 0 0 0 0 0
-2.2348 -2.3484 -0.3161 H 0 0 0 0 0 0 0 0 0 0 0 0
-3.8081 -0.9383 -0.0722 C 0 0 0 0 0 0 0 0 0 0 0 0
-4.7552 -1.9833 0.0358 C 0 0 0 0 0 0 0 0 0 0 0 0
-6.1225 -1.7371 0.0661 C 0 0 0 0 0 0 0 0 0 0 0 0
-6.5975 -0.4311 -0.0252 C 0 0 0 0 0 0 0 0 0 0 0 0
-5.6745 0.6290 -0.1607 C 0 0 0 0 0 0 0 0 0 0 0 0
-4.3101 0.3868 -0.1876 C 0 0 0 0 0 0 0 0 0 0 0 0
-3.6268 1.2098 -0.3378 H 0 0 0 0 0 0 0 0 0 0 0 0

```
-6.2778 1.8610 -0.2697 O 0 0 0 0 0 0 0 0 0 0 0 0
-5.4518 3.0046 -0.4258 C 0 0 0 0 0 0 0 0 0 0 0 0
-4.7756 3.1278 0.4292 H 0 0 0 0 0 0 0 0 0 0 0 0
-4.8605 2.9471 -1.3479 H 0 0 0 0 0 0 0 0 0 0 0 0
-6.1248 3.8612 -0.4811 H 0 0 0 0 0 0 0 0 0 0 0 0
-7.9326 -0.1788 0.0014 O 0 0 0 0 0 0 0 0 0 0 0 0
-8.0413 0.7810 -0.0894 H 0 0 0 0 0 0 0 0 0 0 0 0
-6.8398 -2.5458 0.1577 H 0 0 0 0 0 0 0 0 0 0 0 0
-4.3992 -3.0066 0.1070 H 0 0 0 0 0 0 0 0 0 0 0 0
-0.0000 -1.1737 -0.0868 C 0 0 0 0 0 0 0 0 0 0 0 0
1.2683 -0.5416 0.1288 C 0 0 0 0 0 0 0 0 0 0 0 0
2.4087 -1.2961 -0.1032 C 0 0 0 0 0 0 0 0 0 0 0 0
3.8081 -0.9383 -0.0723 C 0 0 0 0 0 0 0 0 0 0 0 0
4.7552 -1.9833 0.0355 C 0 0 0 0 0 0 0 0 0 0 0 0
6.1225 -1.7371 0.0658 C 0 0 0 0 0 0 0 0 0 0 0 0
6.5975 -0.4311 -0.0253 C 0 0 0 0 0 0 0 0 0 0 0 0
5.6745 0.6290 -0.1606 C 0 0 0 0 0 0 0 0 0 0 0 0
4.3101 0.3868 -0.1875 C 0 0 0 0 0 0 0 0 0 0 0 0
3.6268 1.2098 -0.3375 H 0 0 0 0 0 0 0 0 0 0 0 0
6.2778 1.8610 -0.2695 O 0 0 0 0 0 0 0 0 0 0 0 0
5.4518 3.0046 -0.4253 C 0 0 0 0 0 0 0 0 0 0 0 0
4.8604 2.9474 -1.3473 H 0 0 0 0 0 0 0 0 0 0 0 0
4.7757 3.1277 0.4298 H 0 0 0 0 0 0 0 0 0 0 0 0
6.1248 3.8613 -0.4804 H 0 0 0 0 0 0 0 0 0 0 0 0
7.9326 -0.1789 0.0012 O 0 0 0 0 0 0 0 0 0 0 0 0
8.0413 0.7810 -0.0894 H 0 0 0 0 0 0 0 0 0 0 0 0
6.8398 -2.5458 0.1571 H 0 0 0 0 0 0 0 0 0 0 0 0
4.3991 -3.0066 0.1066 H 0 0 0 0 0 0 0 0 0 0 0 0
2.2348 -2.3484 -0.3163 H 0 0 0 0 0 0 0 0 0 0 0 0
-0.0000 -2.3975 -0.5735 O 0 0 0 0 0 0 0 0 0 0 0 0
-1.3376 1.5925 -0.2251 H 0 0 0 0 0 0 0 0 0 0 0 0
-2.1450 1.0542 1.2439 H 0 0 0 0 0 0 0 0 0 0 0 0
-0.0001 0.6219 2.3483 H 0 0 0 0 0 0 0 0 0 0 0 0
2.1449 1.0542 1.2441 H 0 0 0 0 0 0 0 0 0 0 0 0
1.3377 1.5925 -0.2250 H 0 0 0 0 0 0 0 0 0 0 0 0
1 48 1 0 0 0 0
1 2 1 0 0 0 0
2 3 1 0 0 0 0
2 47 1 0 0 0 0
4 46 1 0 0 0 0
4 2 1 0 0 0 0
5 4 1 0 0 0 0
6 8 1 0 0 0 0
6 5 2 0 0 0 0
7 6 1 0 0 0 0
8 9 2 0 0 0 0
9 10 1 0 0 0 0
9 23 1 0 0 0 0
10 22 1 0 0 0 0
11 20 1 0 0 0 0
```

```
 11 10  2  0  0  0  0
 12 11  1  0  0  0  0
 13 12  2  0  0  0  0
 13  8  1  0  0  0  0
 14 13  1  0  0  0  0
 15 12  1  0  0  0  0
 16 15  1  0  0  0  0
 16 17  1  0  0  0  0
 18 16  1  0  0  0  0
 19 16  1  0  0  0  0
 21 20  1  0  0  0  0
 24 25  2  0  0  0  0
 24  5  1  0  0  0  0
 25  1  1  0  0  0  0
 26 27  1  0  0  0  0
 26 25  1  0  0  0  0
 27 28  2  0  0  0  0
 28 29  1  0  0  0  0
 28 42  1  0  0  0  0
 29 41  1  0  0  0  0
 30 39  1  0  0  0  0
 30 29  2  0  0  0  0
 31 30  1  0  0  0  0
 32 31  2  0  0  0  0
 32 27  1  0  0  0  0
 33 32  1  0  0  0  0
 34 31  1  0  0  0  0
 35 34  1  0  0  0  0
 35 37  1  0  0  0  0
 36 35  1  0  0  0  0
 38 35  1  0  0  0  0
 40 39  1  0  0  0  0
 43 26  1  0  0  0  0
 44 24  1  0  0  0  0
 45  4  1  0  0  0  0
 49  1  1  0  0  0  0
M  END
```

3B': O-CY S$_1$

```
 49 51  0  0  0  0  0  0  0  0999 V2000
    1.2614    0.9010    0.6106 C   0  0  0  0  0  0  0  0  0  0  0  0
    0.0000    1.2252    1.4141 C   0  0  0  0  0  0  0  0  0  0  0  0
    0.0000    2.2826    1.7022 H   0  0  0  0  0  0  0  0  0  0  0  0
   -1.2613    0.9011    0.6105 C   0  0  0  0  0  0  0  0  0  0  0  0
   -1.2685   -0.5319    0.1225 C   0  0  0  0  0  0  0  0  0  0  0  0
   -2.4077   -1.2881   -0.1078 C   0  0  0  0  0  0  0  0  0  0  0  0
   -2.2322   -2.3400   -0.3215 H   0  0  0  0  0  0  0  0  0  0  0  0
   -3.8083   -0.9327   -0.0743 C   0  0  0  0  0  0  0  0  0  0  0  0
   -4.7509   -1.9771    0.0360 C   0  0  0  0  0  0  0  0  0  0  0  0
```

```
-6.1165 -1.7174 0.0687 C 0 0 0 0 0 0 0 0 0 0 0 0
-6.5992 -0.4129 -0.0212 C 0 0 0 0 0 0 0 0 0 0 0 0
-5.6789 0.6539 -0.1613 C 0 0 0 0 0 0 0 0 0 0 0 0
-4.3149 0.3900 -0.1893 C 0 0 0 0 0 0 0 0 0 0 0 0
-3.6282 1.2088 -0.3424 H 0 0 0 0 0 0 0 0 0 0 0 0
-6.2411 1.8913 -0.2720 O 0 0 0 0 0 0 0 0 0 0 0 0
-5.3782 3.0050 -0.4239 C 0 0 0 0 0 0 0 0 0 0 0 0
-4.6980 3.1113 0.4314 H 0 0 0 0 0 0 0 0 0 0 0 0
-4.7851 2.9373 -1.3454 H 0 0 0 0 0 0 0 0 0 0 0 0
-6.0263 3.8808 -0.4785 H 0 0 0 0 0 0 0 0 0 0 0 0
-7.9245 -0.0964 0.0042 O 0 0 0 0 0 0 0 0 0 0 0 0
-8.4331 -0.9131 0.0954 H 0 0 0 0 0 0 0 0 0 0 0 0
-6.8244 -2.5384 0.1641 H 0 0 0 0 0 0 0 0 0 0 0 0
-4.4010 -3.0023 0.1077 H 0 0 0 0 0 0 0 0 0 0 0 0
0.0000 -1.1642 -0.0928 C 0 0 0 0 0 0 0 0 0 0 0 0
1.2685 -0.5319 0.1225 C 0 0 0 0 0 0 0 0 0 0 0 0
2.4077 -1.2880 -0.1081 C 0 0 0 0 0 0 0 0 0 0 0 0
3.8083 -0.9327 -0.0746 C 0 0 0 0 0 0 0 0 0 0 0 0
4.7509 -1.9771 0.0350 C 0 0 0 0 0 0 0 0 0 0 0 0
6.1165 -1.7175 0.0677 C 0 0 0 0 0 0 0 0 0 0 0 0
6.5992 -0.4129 -0.0216 C 0 0 0 0 0 0 0 0 0 0 0 0
5.6789 0.6539 -0.1611 C 0 0 0 0 0 0 0 0 0 0 0 0
4.3149 0.3900 -0.1890 C 0 0 0 0 0 0 0 0 0 0 0 0
3.6282 1.2090 -0.3416 H 0 0 0 0 0 0 0 0 0 0 0 0
6.2411 1.8914 -0.2712 O 0 0 0 0 0 0 0 0 0 0 0 0
5.3782 3.0052 -0.4223 C 0 0 0 0 0 0 0 0 0 0 0 0
4.7849 2.9380 -1.3437 H 0 0 0 0 0 0 0 0 0 0 0 0
4.6982 3.1110 0.4332 H 0 0 0 0 0 0 0 0 0 0 0 0
6.0263 3.8810 -0.4765 H 0 0 0 0 0 0 0 0 0 0 0 0
7.9245 -0.0965 0.0038 O 0 0 0 0 0 0 0 0 0 0 0 0
8.4331 -0.9133 0.0945 H 0 0 0 0 0 0 0 0 0 0 0 0
6.8244 -2.5385 0.1626 H 0 0 0 0 0 0 0 0 0 0 0 0
4.4010 -3.0024 0.1063 H 0 0 0 0 0 0 0 0 0 0 0 0
2.2322 -2.3399 -0.3220 H 0 0 0 0 0 0 0 0 0 0 0 0
-0.0001 -2.3886 -0.5777 O 0 0 0 0 0 0 0 0 0 0 0 0
-1.3386 1.6009 -0.2362 H 0 0 0 0 0 0 0 0 0 0 0 0
-2.1453 1.0669 1.2342 H 0 0 0 0 0 0 0 0 0 0 0 0
-0.0001 0.6372 2.3397 H 0 0 0 0 0 0 0 0 0 0 0 0
2.1453 1.0667 1.2344 H 0 0 0 0 0 0 0 0 0 0 0 0
1.3389 1.6009 -0.2360 H 0 0 0 0 0 0 0 0 0 0 0 0
1 48 1 0 0 0 0
1 2 1 0 0 0 0
2 3 1 0 0 0 0
2 47 1 0 0 0 0
4 46 1 0 0 0 0
4 2 1 0 0 0 0
5 4 1 0 0 0 0
6 8 1 0 0 0 0
6 5 2 0 0 0 0
7 6 1 0 0 0 0
```

8 9 2 0 0 0 0
9 10 1 0 0 0 0
9 23 1 0 0 0 0
10 22 1 0 0 0 0
11 20 1 0 0 0 0
11 10 2 0 0 0 0
12 11 1 0 0 0 0
13 12 2 0 0 0 0
13 8 1 0 0 0 0
14 13 1 0 0 0 0
15 12 1 0 0 0 0
16 15 1 0 0 0 0
16 17 1 0 0 0 0
18 16 1 0 0 0 0
19 16 1 0 0 0 0
20 21 1 0 0 0 0
24 25 2 0 0 0 0
24 5 1 0 0 0 0
25 1 1 0 0 0 0
26 27 1 0 0 0 0
26 25 1 0 0 0 0
27 28 2 0 0 0 0
28 29 1 0 0 0 0
28 42 1 0 0 0 0
29 41 1 0 0 0 0
30 39 1 0 0 0 0
30 29 2 0 0 0 0
31 30 1 0 0 0 0
32 31 2 0 0 0 0
32 27 1 0 0 0 0
33 32 1 0 0 0 0
34 31 1 0 0 0 0
35 34 1 0 0 0 0
35 37 1 0 0 0 0
36 35 1 0 0 0 0
38 35 1 0 0 0 0
39 40 1 0 0 0 0
43 26 1 0 0 0 0
44 24 1 0 0 0 0
45 4 1 0 0 0 0
49 1 1 0 0 0 0
M END

TEMPO Adducts

1A: CCE-Curc S_0

47 48 0 0 0 0 0 0 0999 V2000
-8.4773 2.2854 0.0001 C 0 0 0 0 0 0 0 0 0 0 0 0
-8.5754 0.8658 0.0001 O 0 0 0 0 0 0 0 0 0 0 0 0
-7.4167 0.1301 0.0000 C 0 0 0 0 0 0 0 0 0 0 0 0

```
-7.6335 -1.2616 0.0000 C 0 0 0 0 0 0 0 0 0 0 0 0
-6.5418 -2.1237 -0.0000 C 0 0 0 0 0 0 0 0 0 0 0 0
-5.2487 -1.6172 -0.0000 C 0 0 0 0 0 0 0 0 0 0 0 0
-5.0125 -0.2342 -0.0000 C 0 0 0 0 0 0 0 0 0 0 0 0
-6.1264 0.6326 0.0000 C 0 0 0 0 0 0 0 0 0 0 0 0
-5.9539 1.7012 0.0000 H 0 0 0 0 0 0 0 0 0 0 0 0
-3.6816 0.3568 -0.0000 C 0 0 0 0 0 0 0 0 0 0 0 0
-2.4882 -0.2666 -0.0001 C 0 0 0 0 0 0 0 0 0 0 0 0
-1.2314 0.5002 -0.0001 C 0 0 0 0 0 0 0 0 0 0 0 0
0.0071 -0.2330 -0.0001 C 0 0 0 0 0 0 0 0 0 0 0 0
1.2188 0.4306 -0.0001 C 0 0 0 0 0 0 0 0 0 0 0 0
2.4851 -0.2760 -0.0001 C 0 0 0 0 0 0 0 0 0 0 0 0
3.6749 0.3596 -0.0000 C 0 0 0 0 0 0 0 0 0 0 0 0
5.0049 -0.2332 0.0000 C 0 0 0 0 0 0 0 0 0 0 0 0
5.2405 -1.6164 0.0000 C 0 0 0 0 0 0 0 0 0 0 0 0
6.5332 -2.1238 0.0001 C 0 0 0 0 0 0 0 0 0 0 0 0
7.6258 -1.2631 0.0001 C 0 0 0 0 0 0 0 0 0 0 0 0
7.4099 0.1288 0.0000 C 0 0 0 0 0 0 0 0 0 0 0 0
6.1200 0.6325 0.0000 C 0 0 0 0 0 0 0 0 0 0 0 0
5.9497 1.7015 -0.0000 H 0 0 0 0 0 0 0 0 0 0 0 0
8.5689 0.8635 0.0000 O 0 0 0 0 0 0 0 0 0 0 0 0
8.4727 2.2833 -0.0000 C 0 0 0 0 0 0 0 0 0 0 0 0
9.4962 2.6536 -0.0000 H 0 0 0 0 0 0 0 0 0 0 0 0
7.9546 2.6438 -0.8946 H 0 0 0 0 0 0 0 0 0 0 0 0
7.9546 2.6438 0.8946 H 0 0 0 0 0 0 0 0 0 0 0 0
8.8875 -1.7594 0.0001 O 0 0 0 0 0 0 0 0 0 0 0 0
9.4931 -1.0052 0.0001 H 0 0 0 0 0 0 0 0 0 0 0 0
6.7193 -3.1909 0.0001 H 0 0 0 0 0 0 0 0 0 0 0 0
4.4083 -2.3092 0.0001 H 0 0 0 0 0 0 0 0 0 0 0 0
3.6429 1.4454 0.0000 H 0 0 0 0 0 0 0 0 0 0 0 0
2.4174 -1.3578 -0.0001 H 0 0 0 0 0 0 0 0 0 0 0 0
1.2732 1.7583 -0.0000 O 0 0 0 0 0 0 0 0 0 0 0 0
0.3108 2.0636 -0.0000 H 0 0 0 0 0 0 0 0 0 0 0 0
-0.0031 -1.3142 -0.0001 H 0 0 0 0 0 0 0 0 0 0 0 0
-1.2446 1.7580 -0.0000 O 0 0 0 0 0 0 0 0 0 0 0 0
-2.4045 -1.3481 -0.0001 H 0 0 0 0 0 0 0 0 0 0 0 0
-3.6407 1.4437 -0.0000 H 0 0 0 0 0 0 0 0 0 0 0 0
-4.4158 -2.3091 -0.0001 H 0 0 0 0 0 0 0 0 0 0 0 0
-6.7292 -3.1905 -0.0000 H 0 0 0 0 0 0 0 0 0 0 0 0
-8.8958 -1.7567 0.0000 O 0 0 0 0 0 0 0 0 0 0 0 0
-9.5003 -1.0016 0.0001 H 0 0 0 0 0 0 0 0 0 0 0 0
-7.9586 2.6453 -0.8944 H 0 0 0 0 0 0 0 0 0 0 0 0
-9.5004 2.6571 0.0001 H 0 0 0 0 0 0 0 0 0 0 0 0
-7.9586 2.6452 0.8946 H 0 0 0 0 0 0 0 0 0 0 0 0
1 46 1 0 0 0 0
1 47 1 0 0 0 0
2 1 1 0 0 0 0
3 2 1 0 0 0 0
4 3 2 0 0 0 0
4 43 1 0 0 0 0
```

```
5 4 1 0 0 0 0
6 5 2 0 0 0 0
6 7 1 0 0 0 0
7 8 2 0 0 0 0
8 3 1 0 0 0 0
8 9 1 0 0 0 0
10 7 1 0 0 0 0
10 40 1 0 0 0 0
11 10 2 0 0 0 0
12 11 1 0 0 0 0
12 38 2 0 0 0 0
13 12 1 0 0 0 0
13 14 2 0 0 0 0
14 15 1 0 0 0 0
14 35 1 0 0 0 0
15 16 2 0 0 0 0
16 33 1 0 0 0 0
16 17 1 0 0 0 0
17 18 1 0 0 0 0
18 32 1 0 0 0 0
18 19 2 0 0 0 0
19 31 1 0 0 0 0
20 19 1 0 0 0 0
20 29 1 0 0 0 0
21 24 1 0 0 0 0
21 20 2 0 0 0 0
22 17 2 0 0 0 0
22 21 1 0 0 0 0
23 22 1 0 0 0 0
25 26 1 0 0 0 0
25 24 1 0 0 0 0
25 28 1 0 0 0 0
27 25 1 0 0 0 0
30 29 1 0 0 0 0
34 15 1 0 0 0 0
35 36 1 0 0 0 0
37 13 1 0 0 0 0
39 11 1 0 0 0 0
41 6 1 0 0 0 0
42 5 1 0 0 0 0
43 44 1 0 0 0 0
45 1 1 0 0 0 0
M END
```

3A:T-DIK-Curc S_0

```
47 48 0 0 0 0 0 0 0 0999 V2000
 -0.0025 3.2765 -0.2923 C 0 0 0 0 0 0 0 0 0 0 0 0
 -0.9756 2.3132 -0.9800 C 0 0 0 0 0 0 0 0 0 0 0 0
 -2.0359 1.7492 -0.1217 C 0 0 0 0 0 0 0 0 0 0 0 0
```

```
-2.9698   0.9268  -0.6386 C  0 0 0 0 0 0 0 0 0 0 0 0
-4.0816   0.2793   0.0395 C  0 0 0 0 0 0 0 0 0 0 0 0
-4.3325   0.4115   1.4138 C  0 0 0 0 0 0 0 0 0 0 0 0
-5.4091  -0.2315   2.0090 C  0 0 0 0 0 0 0 0 0 0 0 0
-6.2629  -1.0227   1.2468 C  0 0 0 0 0 0 0 0 0 0 0 0
-6.0265  -1.1681  -0.1347 C  0 0 0 0 0 0 0 0 0 0 0 0
-4.9520  -0.5264  -0.7261 C  0 0 0 0 0 0 0 0 0 0 0 0
-4.7643  -0.6340  -1.7867 H  0 0 0 0 0 0 0 0 0 0 0 0
-6.9407  -1.9736  -0.7654 O  0 0 0 0 0 0 0 0 0 0 0 0
-6.8051  -2.1862  -2.1662 C  0 0 0 0 0 0 0 0 0 0 0 0
-7.6266  -2.8408  -2.4514 H  0 0 0 0 0 0 0 0 0 0 0 0
-6.8816  -1.2432  -2.7172 H  0 0 0 0 0 0 0 0 0 0 0 0
-5.8528  -2.6727  -2.4014 H  0 0 0 0 0 0 0 0 0 0 0 0
-7.3137  -1.6493   1.8283 O  0 0 0 0 0 0 0 0 0 0 0 0
-7.7754  -2.1411   1.1349 H  0 0 0 0 0 0 0 0 0 0 0 0
-5.6078  -0.1343   3.0694 H  0 0 0 0 0 0 0 0 0 0 0 0
-3.6802   1.0200   2.0275 H  0 0 0 0 0 0 0 0 0 0 0 0
-2.8770   0.7249  -1.7040 H  0 0 0 0 0 0 0 0 0 0 0 0
-2.0217   2.0250   0.9269 H  0 0 0 0 0 0 0 0 0 0 0 0
-0.8412   2.0305  -2.1588 O  0 0 0 0 0 0 0 0 0 0 0 0
 1.0072   2.4999   0.5626 C  0 0 0 0 0 0 0 0 0 0 0 0
 2.0291   1.7402  -0.1840 C  0 0 0 0 0 0 0 0 0 0 0 0
 2.9940   1.0653   0.4707 C  0 0 0 0 0 0 0 0 0 0 0 0
 4.0743   0.2657  -0.0854 C  0 0 0 0 0 0 0 0 0 0 0 0
 4.9858  -0.3389   0.8071 C  0 0 0 0 0 0 0 0 0 0 0 0
 6.0309  -1.1137   0.3341 C  0 0 0 0 0 0 0 0 0 0 0 0
 6.1952  -1.3086  -1.0517 C  0 0 0 0 0 0 0 0 0 0 0 0
 5.3010  -0.7166  -1.9382 C  0 0 0 0 0 0 0 0 0 0 0 0
 4.2540   0.0606  -1.4620 C  0 0 0 0 0 0 0 0 0 0 0 0
 3.5688   0.5087  -2.1704 H  0 0 0 0 0 0 0 0 0 0 0 0
 5.4445  -0.8799  -2.9993 H  0 0 0 0 0 0 0 0 0 0 0 0
 7.2164  -2.0674  -1.5170 O  0 0 0 0 0 0 0 0 0 0 0 0
 7.7135  -2.3827  -0.7493 H  0 0 0 0 0 0 0 0 0 0 0 0
 6.9794  -1.7516   1.0932 O  0 0 0 0 0 0 0 0 0 0 0 0
 6.9164  -1.6202   2.5089 C  0 0 0 0 0 0 0 0 0 0 0 0
 5.9787  -2.0263   2.9019 H  0 0 0 0 0 0 0 0 0 0 0 0
 7.7530  -2.1954   2.9013 H  0 0 0 0 0 0 0 0 0 0 0 0
 7.0192  -0.5734   2.8129 H  0 0 0 0 0 0 0 0 0 0 0 0
 4.8531  -0.1864   1.8705 H  0 0 0 0 0 0 0 0 0 0 0 0
 2.9565   1.1294   1.5564 H  0 0 0 0 0 0 0 0 0 0 0 0
 1.9589   1.7505  -1.2660 H  0 0 0 0 0 0 0 0 0 0 0 0
 0.9319   2.5148   1.7793 O  0 0 0 0 0 0 0 0 0 0 0 0
 0.5144   3.8337  -1.0757 H  0 0 0 0 0 0 0 0 0 0 0 0
-0.5361   3.9581   0.3717 H  0 0 0 0 0 0 0 0 0 0 0 0
1 47 1 0 0 0 0
1 24 1 0 0 0 0
2 1 1 0 0 0 0
2 3 1 0 0 0 0
3 22 1 0 0 0 0
4 3 2 0 0 0 0
```

4 5 1 0 0 0 0
5 6 2 0 0 0 0
6 7 1 0 0 0 0
6 20 1 0 0 0 0
7 19 1 0 0 0 0
8 17 1 0 0 0 0
8 7 2 0 0 0 0
9 8 1 0 0 0 0
10 9 2 0 0 0 0
10 5 1 0 0 0 0
11 10 1 0 0 0 0
12 9 1 0 0 0 0
13 12 1 0 0 0 0
14 13 1 0 0 0 0
15 13 1 0 0 0 0
16 13 1 0 0 0 0
18 17 1 0 0 0 0
21 4 1 0 0 0 0
23 2 2 0 0 0 0
24 45 2 0 0 0 0
25 26 2 0 0 0 0
25 24 1 0 0 0 0
26 43 1 0 0 0 0
27 26 1 0 0 0 0
27 28 2 0 0 0 0
28 42 1 0 0 0 0
29 28 1 0 0 0 0
29 37 1 0 0 0 0
30 29 2 0 0 0 0
31 32 2 0 0 0 0
31 30 1 0 0 0 0
32 27 1 0 0 0 0
33 32 1 0 0 0 0
34 31 1 0 0 0 0
35 30 1 0 0 0 0
35 36 1 0 0 0 0
37 38 1 0 0 0 0
38 41 1 0 0 0 0
38 40 1 0 0 0 0
38 39 1 0 0 0 0
44 25 1 0 0 0 0
46 1 1 0 0 0 0
M END

4A:OCE-Curc S_0

47 48 0 0 0 0 0 0 0999 V2000
-8.6970 -1.9094 -0.0765 C 0 0 0 0 0 0 0 0 0 0 0 0
-8.6289 -0.4966 0.0759 O 0 0 0 0 0 0 0 0 0 0 0 0
-7.3919 0.0992 0.1049 C 0 0 0 0 0 0 0 0 0 0 0 0

-7.4445 1.4979 0.2575 C 0 0 0 0 0 0 0 0 0 0 0 0

-6.2597 2.2248 0.3018 C 0 0 0 0 0 0 0 0 0 0 0 0

-5.0352 1.5771 0.1976 C 0 0 0 0 0 0 0 0 0 0 0 0

-4.9623 0.1845 0.0460 C 0 0 0 0 0 0 0 0 0 0 0 0

-6.1697 -0.5443 0.0015 C 0 0 0 0 0 0 0 0 0 0 0 0

-6.1238 -1.6195 -0.1149 H 0 0 0 0 0 0 0 0 0 0 0 0

-3.7085 -0.5510 -0.0667 C 0 0 0 0 0 0 0 0 0 0 0 0

-2.4519 -0.0738 -0.0518 C 0 0 0 0 0 0 0 0 0 0 0 0

-1.2893 -0.9976 -0.1818 C 0 0 0 0 0 0 0 0 0 0 0 0

0.0219 -0.3340 -0.1682 C 0 0 0 0 0 0 0 0 0 0 0 0

1.2407 -0.9218 -0.3204 C 0 0 0 0 0 0 0 0 0 0 0 0

2.4569 -0.1182 -0.3078 C 0 0 0 0 0 0 0 0 0 0 0 0

3.7048 -0.6124 -0.1734 C 0 0 0 0 0 0 0 0 0 0 0 0

4.9613 0.1298 -0.1752 C 0 0 0 0 0 0 0 0 0 0 0 0

5.0563 1.4840 -0.5263 C 0 0 0 0 0 0 0 0 0 0 0 0

6.2768 2.1472 -0.5017 C 0 0 0 0 0 0 0 0 0 0 0 0

7.4351 1.4751 -0.1286 C 0 0 0 0 0 0 0 0 0 0 0 0

7.3605 0.1124 0.2204 C 0 0 0 0 0 0 0 0 0 0 0 0

6.1431 -0.5477 0.1934 C 0 0 0 0 0 0 0 0 0 0 0 0

6.0851 -1.5942 0.4654 H 0 0 0 0 0 0 0 0 0 0 0 0

8.5716 -0.4322 0.5646 O 0 0 0 0 0 0 0 0 0 0 0 0

8.6182 -1.8013 0.9492 C 0 0 0 0 0 0 0 0 0 0 0 0

9.6603 -2.0128 1.1812 H 0 0 0 0 0 0 0 0 0 0 0 0

8.0032 -1.9831 1.8366 H 0 0 0 0 0 0 0 0 0 0 0 0

8.2878 -2.4526 0.1333 H 0 0 0 0 0 0 0 0 0 0 0 0

8.6259 2.1229 -0.1074 O 0 0 0 0 0 0 0 0 0 0 0 0

9.2963 1.4848 0.1730 H 0 0 0 0 0 0 0 0 0 0 0 0

6.3544 3.1919 -0.7770 H 0 0 0 0 0 0 0 0 0 0 0 0

4.1733 2.0282 -0.8372 H 0 0 0 0 0 0 0 0 0 0 0 0

3.8227 -1.6828 -0.0080 H 0 0 0 0 0 0 0 0 0 0 0 0

2.3002 0.9513 -0.3865 H 0 0 0 0 0 0 0 0 0 0 0 0

1.3523 -2.2624 -0.4564 O 0 0 0 0 0 0 0 0 0 0 0 0

2.2271 -2.4662 -0.8052 H 0 0 0 0 0 0 0 0 0 0 0 0

0.0292 0.7406 -0.0314 H 0 0 0 0 0 0 0 0 0 0 0 0

-1.4416 -2.2087 -0.2981 O 0 0 0 0 0 0 0 0 0 0 0 0

-2.2461 0.9867 0.0535 H 0 0 0 0 0 0 0 0 0 0 0 0

-3.7925 -1.6304 -0.1772 H 0 0 0 0 0 0 0 0 0 0 0 0

-4.1269 2.1653 0.2358 H 0 0 0 0 0 0 0 0 0 0 0 0

-6.3204 3.3000 0.4193 H 0 0 0 0 0 0 0 0 0 0 0 0

-8.6409 2.1302 0.3603 O 0 0 0 0 0 0 0 0 0 0 0 0

-9.3277 1.4513 0.3093 H 0 0 0 0 0 0 0 0 0 0 0 0

-8.1996 -2.4212 0.7539 H 0 0 0 0 0 0 0 0 0 0 0 0

-9.7562 -2.1610 -0.0749 H 0 0 0 0 0 0 0 0 0 0 0 0

-8.2483 -2.2268 -1.0235 H 0 0 0 0 0 0 0 0 0 0 0 0

1 46 1 0 0 0 0

1 2 1 0 0 0 0

1 45 1 0 0 0 0

2 3 1 0 0 0 0

3 4 2 0 0 0 0

4 5 1 0 0 0 0

4 43 1 0 0 0 0
5 42 1 0 0 0 0
6 41 1 0 0 0 0
6 5 2 0 0 0 0
7 6 1 0 0 0 0
8 7 2 0 0 0 0
8 3 1 0 0 0 0
9 8 1 0 0 0 0
10 11 2 0 0 0 0
10 7 1 0 0 0 0
11 39 1 0 0 0 0
12 13 1 0 0 0 0
12 11 1 0 0 0 0
13 37 1 0 0 0 0
14 15 1 0 0 0 0
14 13 2 0 0 0 0
15 16 2 0 0 0 0
16 33 1 0 0 0 0
17 16 1 0 0 0 0
17 22 2 0 0 0 0
18 19 2 0 0 0 0
18 17 1 0 0 0 0
19 20 1 0 0 0 0
20 29 1 0 0 0 0
20 21 2 0 0 0 0
21 24 1 0 0 0 0
22 21 1 0 0 0 0
22 23 1 0 0 0 0
24 25 1 0 0 0 0
25 26 1 0 0 0 0
25 27 1 0 0 0 0
28 25 1 0 0 0 0
29 30 1 0 0 0 0
31 19 1 0 0 0 0
32 18 1 0 0 0 0
34 15 1 0 0 0 0
35 14 1 0 0 0 0
36 35 1 0 0 0 0
38 12 2 0 0 0 0
40 10 1 0 0 0 0
44 43 1 0 0 0 0
47 1 1 0 0 0 0
M END

5A:T-E-Curc S₀

47 48 0 0 0 0 0 0 0 0999 V2000
8.4372 -0.5324 1.3578 C 0 0 0 0 0 0 0 0 0 0 0 0
8.0226 0.6190 0.6306 O 0 0 0 0 0 0 0 0 0 0 0 0
6.7363 0.6654 0.1563 C 0 0 0 0 0 0 0 0 0 0 0 0

```
6.4422 1.8421 -0.5606 C 0 0 0 0 0 0 0 0 0 0 0 0
5.1720 2.0147 -1.1013 C 0 0 0 0 0 0 0 0 0 0 0 0
4.1999 1.0374 -0.9330 C 0 0 0 0 0 0 0 0 0 0 0 0
4.4726 -0.1383 -0.2180 C 0 0 0 0 0 0 0 0 0 0 0 0
5.7656 -0.3078 0.3217 C 0 0 0 0 0 0 0 0 0 0 0 0
5.9855 -1.2148 0.8701 H 0 0 0 0 0 0 0 0 0 0 0 0
3.4958 -1.1974 -0.0050 C 0 0 0 0 0 0 0 0 0 0 0 0
2.1915 -1.2184 -0.3416 C 0 0 0 0 0 0 0 0 0 0 0 0
1.3649 -2.4342 -0.0832 C 0 0 0 0 0 0 0 0 0 0 0 0
-0.0973 -2.3367 -0.1708 C 0 0 0 0 0 0 0 0 0 0 0 0
-0.8941 -1.2464 0.0131 C 0 0 0 0 0 0 0 0 0 0 0 0
-2.3358 -1.3278 -0.1220 C 0 0 0 0 0 0 0 0 0 0 0 0
-3.1684 -0.2908 0.1012 C 0 0 0 0 0 0 0 0 0 0 0 0
-4.6208 -0.2745 -0.0230 C 0 0 0 0 0 0 0 0 0 0 0 0
-5.3807 -1.3824 -0.4270 C 0 0 0 0 0 0 0 0 0 0 0 0
-6.7644 -1.3075 -0.5225 C 0 0 0 0 0 0 0 0 0 0 0 0
-7.4273 -0.1236 -0.2180 C 0 0 0 0 0 0 0 0 0 0 0 0
-6.6820 1.0002 0.1876 C 0 0 0 0 0 0 0 0 0 0 0 0
-5.3024 0.9232 0.2824 C 0 0 0 0 0 0 0 0 0 0 0 0
-4.7270 1.7855 0.5943 H 0 0 0 0 0 0 0 0 0 0 0 0
-7.4539 2.1030 0.4571 O 0 0 0 0 0 0 0 0 0 0 0 0
-6.8067 3.2988 0.8746 C 0 0 0 0 0 0 0 0 0 0 0 0
-7.5977 4.0304 1.0294 H 0 0 0 0 0 0 0 0 0 0 0 0
-6.2625 3.1468 1.8126 H 0 0 0 0 0 0 0 0 0 0 0 0
-6.1176 3.6642 0.1060 H 0 0 0 0 0 0 0 0 0 0 0 0
-8.7786 -0.0513 -0.3118 O 0 0 0 0 0 0 0 0 0 0 0 0
-9.0358 0.8466 -0.0611 H 0 0 0 0 0 0 0 0 0 0 0 0
-7.3537 -2.1613 -0.8341 H 0 0 0 0 0 0 0 0 0 0 0 0
-4.8918 -2.3170 -0.6722 H 0 0 0 0 0 0 0 0 0 0 0 0
-2.7151 0.6460 0.4109 H 0 0 0 0 0 0 0 0 0 0 0 0
-2.7119 -2.2966 -0.4289 H 0 0 0 0 0 0 0 0 0 0 0 0
-0.4422 -0.0064 0.3496 O 0 0 0 0 0 0 0 0 0 0 0 0
0.4903 -0.0588 0.5948 H 0 0 0 0 0 0 0 0 0 0 0 0
-0.5935 -3.2887 -0.3148 H 0 0 0 0 0 0 0 0 0 0 0 0
1.8962 -3.5130 0.1674 O 0 0 0 0 0 0 0 0 0 0 0 0
1.7326 -0.3849 -0.8652 H 0 0 0 0 0 0 0 0 0 0 0 0
3.8632 -2.0942 0.4892 H 0 0 0 0 0 0 0 0 0 0 0 0
3.2221 1.1909 -1.3727 H 0 0 0 0 0 0 0 0 0 0 0 0
4.9685 2.9226 -1.6560 H 0 0 0 0 0 0 0 0 0 0 0 0
7.3906 2.7955 -0.7261 O 0 0 0 0 0 0 0 0 0 0 0 0
8.1932 2.4819 -0.2864 H 0 0 0 0 0 0 0 0 0 0 0 0
8.3674 -1.4346 0.7416 H 0 0 0 0 0 0 0 0 0 0 0 0
9.4767 -0.3577 1.6290 H 0 0 0 0 0 0 0 0 0 0 0 0
7.8403 -0.6614 2.2665 H 0 0 0 0 0 0 0 0 0 0 0 0
1 46 1 0 0 0 0
1 47 1 0 0 0 0
2 1 1 0 0 0 0
3 8 1 0 0 0 0
3 2 1 0 0 0 0
4 3 2 0 0 0 0
```

```
 5  6  2  0  0  0  0
 5  4  1  0  0  0  0
 6  7  1  0  0  0  0
 7 10  1  0  0  0  0
 7  8  2  0  0  0  0
 8  9  1  0  0  0  0
10 40  1  0  0  0  0
11 12  1  0  0  0  0
11 10  2  0  0  0  0
12 38  2  0  0  0  0
13 12  1  0  0  0  0
13 14  2  0  0  0  0
14 35  1  0  0  0  0
15 14  1  0  0  0  0
15 16  2  0  0  0  0
16 33  1  0  0  0  0
17 16  1  0  0  0  0
17 22  2  0  0  0  0
18 17  1  0  0  0  0
19 18  2  0  0  0  0
19 20  1  0  0  0  0
20 21  2  0  0  0  0
21 22  1  0  0  0  0
21 24  1  0  0  0  0
22 23  1  0  0  0  0
24 25  1  0  0  0  0
25 26  1  0  0  0  0
25 27  1  0  0  0  0
28 25  1  0  0  0  0
29 20  1  0  0  0  0
29 30  1  0  0  0  0
31 19  1  0  0  0  0
32 18  1  0  0  0  0
34 15  1  0  0  0  0
35 36  1  0  0  0  0
37 13  1  0  0  0  0
39 11  1  0  0  0  0
41  6  1  0  0  0  0
42  5  1  0  0  0  0
43  4  1  0  0  0  0
43 44  1  0  0  0  0
45  1  1  0  0  0  0
M  END
```

1B:DM-Curc S_0

```
53 54  0  0  0  0  0  0  0  0999 V2000
    9.2180    1.4284   -0.9729 C   0  0  0  0  0  0  0  0  0  0  0  0
    8.4339    1.2354    0.2141 O   0  0  0  0  0  0  0  0  0  0  0  0
    7.3838    0.3573    0.0765 C   0  0  0  0  0  0  0  0  0  0  0  0
```

7.5970	-1.0248	-0.0617	C	0	0	0	0	0	0	0	0	0	0	0	0
6.4955	-1.8725	-0.1968	C	0	0	0	0	0	0	0	0	0	0	0	0
5.2032	-1.3717	-0.1722	C	0	0	0	0	0	0	0	0	0	0	0	0
4.9694	0.0043	-0.0006	C	0	0	0	0	0	0	0	0	0	0	0	0
6.0850	0.8499	0.1149	C	0	0	0	0	0	0	0	0	0	0	0	0
5.9528	1.9192	0.2364	H	0	0	0	0	0	0	0	0	0	0	0	0
3.6370	0.5969	0.0518	C	0	0	0	0	0	0	0	0	0	0	0	0
2.4531	-0.0379	-0.0531	C	0	0	0	0	0	0	0	0	0	0	0	0
1.1790	0.6542	0.0175	C	0	0	0	0	0	0	0	0	0	0	0	0
-0.0237	-0.0151	-0.0880	C	0	0	0	0	0	0	0	0	0	0	0	0
-1.2714	0.6997	-0.0088	C	0	0	0	0	0	0	0	0	0	0	0	0
-2.5189	-0.0757	-0.1229	C	0	0	0	0	0	0	0	0	0	0	0	0
-3.7207	0.5240	-0.0437	C	0	0	0	0	0	0	0	0	0	0	0	0
-5.0410	-0.0890	-0.1314	C	0	0	0	0	0	0	0	0	0	0	0	0
-5.2473	-1.4552	-0.3668	C	0	0	0	0	0	0	0	0	0	0	0	0
-6.5357	-1.9677	-0.4241	C	0	0	0	0	0	0	0	0	0	0	0	0
-7.6519	-1.1496	-0.2629	C	0	0	0	0	0	0	0	0	0	0	0	0
-7.4635	0.2337	-0.0483	C	0	0	0	0	0	0	0	0	0	0	0	0
-6.1691	0.7399	0.0223	C	0	0	0	0	0	0	0	0	0	0	0	0
-6.0089	1.7971	0.1882	H	0	0	0	0	0	0	0	0	0	0	0	0
-8.5949	0.9882	0.0596	O	0	0	0	0	0	0	0	0	0	0	0	0
-8.4592	2.3949	0.2199	C	0	0	0	0	0	0	0	0	0	0	0	0
-9.4739	2.7874	0.2562	H	0	0	0	0	0	0	0	0	0	0	0	0
-7.9399	2.6454	1.1513	H	0	0	0	0	0	0	0	0	0	0	0	0
-7.9276	2.8430	-0.6262	H	0	0	0	0	0	0	0	0	0	0	0	0
-8.8899	-1.7122	-0.3944	O	0	0	0	0	0	0	0	0	0	0	0	0
-9.7693	-1.6345	0.7356	C	0	0	0	0	0	0	0	0	0	0	0	0
-10.6500	-2.2159	0.4651	H	0	0	0	0	0	0	0	0	0	0	0	0
-9.2985	-2.0797	1.6190	H	0	0	0	0	0	0	0	0	0	0	0	0
-10.0561	-0.6042	0.9484	H	0	0	0	0	0	0	0	0	0	0	0	0
-6.7126	-3.0210	-0.6066	H	0	0	0	0	0	0	0	0	0	0	0	0
-4.4051	-2.1225	-0.5002	H	0	0	0	0	0	0	0	0	0	0	0	0
-3.7002	1.6005	0.1092	H	0	0	0	0	0	0	0	0	0	0	0	0
-2.4203	-1.1461	-0.2685	H	0	0	0	0	0	0	0	0	0	0	0	0
-1.2997	1.9451	0.1550	O	0	0	0	0	0	0	0	0	0	0	0	0
-0.0212	-1.0872	-0.2285	H	0	0	0	0	0	0	0	0	0	0	0	0
1.2221	1.9699	0.1910	O	0	0	0	0	0	0	0	0	0	0	0	0
0.2590	2.2677	0.2169	H	0	0	0	0	0	0	0	0	0	0	0	0
2.3937	-1.1108	-0.1959	H	0	0	0	0	0	0	0	0	0	0	0	0
3.6049	1.6731	0.1927	H	0	0	0	0	0	0	0	0	0	0	0	0
4.3719	-2.0583	-0.2745	H	0	0	0	0	0	0	0	0	0	0	0	0
6.6838	-2.9328	-0.3183	H	0	0	0	0	0	0	0	0	0	0	0	0
8.8578	-1.5630	-0.1138	O	0	0	0	0	0	0	0	0	0	0	0	0
9.6161	-1.4774	1.1038	C	0	0	0	0	0	0	0	0	0	0	0	0
9.1125	-2.0285	1.9047	H	0	0	0	0	0	0	0	0	0	0	0	0
10.5775	-1.9442	0.8925	H	0	0	0	0	0	0	0	0	0	0	0	0
9.7629	-0.4388	1.4073	H	0	0	0	0	0	0	0	0	0	0	0	0
9.6710	0.4910	-1.3048	H	0	0	0	0	0	0	0	0	0	0	0	0
8.6007	1.8438	-1.7763	H	0	0	0	0	0	0	0	0	0	0	0	0
9.9962	2.1436	-0.7088	H	0	0	0	0	0	0	0	0	0	0	0	0

1	53	1	0	0	0	0
1	2	1	0	0	0	0
3	8	1	0	0	0	0
3	2	1	0	0	0	0
4	3	2	0	0	0	0
5	6	2	0	0	0	0
5	4	1	0	0	0	0
6	7	1	0	0	0	0
7	10	1	0	0	0	0
7	8	2	0	0	0	0
8	9	1	0	0	0	0
10	43	1	0	0	0	0
11	12	1	0	0	0	0
11	10	2	0	0	0	0
12	40	1	0	0	0	0
13	14	1	0	0	0	0
13	12	2	0	0	0	0
14	38	2	0	0	0	0
15	16	2	0	0	0	0
15	14	1	0	0	0	0
16	36	1	0	0	0	0
17	16	1	0	0	0	0
17	22	2	0	0	0	0
18	17	1	0	0	0	0
19	18	2	0	0	0	0
19	20	1	0	0	0	0
20	21	2	0	0	0	0
21	22	1	0	0	0	0
21	24	1	0	0	0	0
22	23	1	0	0	0	0
24	25	1	0	0	0	0
25	26	1	0	0	0	0
25	27	1	0	0	0	0
28	25	1	0	0	0	0
29	20	1	0	0	0	0
29	30	1	0	0	0	0
30	33	1	0	0	0	0
30	32	1	0	0	0	0
31	30	1	0	0	0	0
34	19	1	0	0	0	0
35	18	1	0	0	0	0
37	15	1	0	0	0	0
39	13	1	0	0	0	0
40	41	1	0	0	0	0
42	11	1	0	0	0	0
44	6	1	0	0	0	0
45	5	1	0	0	0	0
46	4	1	0	0	0	0
46	47	1	0	0	0	0
47	50	1	0	0	0	0

```
47 48  1  0  0  0  0
49 47  1  0  0  0  0
51  1  1  0  0  0  0
52  1  1  0  0  0  0
M  END
```

2B:BDeM-Curc S_0

```
39 40  0  0  0  0  0  0  0  0999 V2000
  -5.2485   -1.2645   -0.0008 C   0  0  0  0  0  0  0  0  0  0  0  0
  -6.5315   -1.7799   -0.0005 C   0  0  0  0  0  0  0  0  0  0  0  0
  -7.6331   -0.9151    0.0004 C   0  0  0  0  0  0  0  0  0  0  0  0
  -7.4277    0.4656    0.0010 C   0  0  0  0  0  0  0  0  0  0  0  0
  -6.1335    0.9702    0.0007 C   0  0  0  0  0  0  0  0  0  0  0  0
  -5.0127    0.1248   -0.0001 C   0  0  0  0  0  0  0  0  0  0  0  0
  -3.6826    0.7178   -0.0003 C   0  0  0  0  0  0  0  0  0  0  0  0
  -2.4895    0.0948   -0.0004 C   0  0  0  0  0  0  0  0  0  0  0  0
  -1.2316    0.8614   -0.0006 C   0  0  0  0  0  0  0  0  0  0  0  0
   0.0068    0.1276   -0.0004 C   0  0  0  0  0  0  0  0  0  0  0  0
   1.2181    0.7915   -0.0003 C   0  0  0  0  0  0  0  0  0  0  0  0
   2.4850    0.0846   -0.0002 C   0  0  0  0  0  0  0  0  0  0  0  0
   3.6747    0.7193   -0.0002 C   0  0  0  0  0  0  0  0  0  0  0  0
   5.0039    0.1244   -0.0000 C   0  0  0  0  0  0  0  0  0  0  0  0
   6.1259    0.9686    0.0004 C   0  0  0  0  0  0  0  0  0  0  0  0
   7.4195    0.4629    0.0006 C   0  0  0  0  0  0  0  0  0  0  0  0
   7.6242   -0.9178    0.0004 C   0  0  0  0  0  0  0  0  0  0  0  0
   6.5218   -1.7815   -0.0001 C   0  0  0  0  0  0  0  0  0  0  0  0
   5.2391   -1.2652   -0.0003 C   0  0  0  0  0  0  0  0  0  0  0  0
   4.4030   -1.9544   -0.0006 H   0  0  0  0  0  0  0  0  0  0  0  0
   6.6986   -2.8500   -0.0002 H   0  0  0  0  0  0  0  0  0  0  0  0
   8.8624   -1.4840    0.0005 O   0  0  0  0  0  0  0  0  0  0  0  0
   9.5317   -0.7914    0.0009 H   0  0  0  0  0  0  0  0  0  0  0  0
   8.2691    1.1395    0.0009 H   0  0  0  0  0  0  0  0  0  0  0  0
   5.9775    2.0430    0.0005 H   0  0  0  0  0  0  0  0  0  0  0  0
   3.6465    1.8051   -0.0003 H   0  0  0  0  0  0  0  0  0  0  0  0
   2.4163   -0.9973   -0.0001 H   0  0  0  0  0  0  0  0  0  0  0  0
   1.2734    2.1184   -0.0003 O   0  0  0  0  0  0  0  0  0  0  0  0
   0.3117    2.4251   -0.0004 H   0  0  0  0  0  0  0  0  0  0  0  0
  -0.0032   -0.9535   -0.0003 H   0  0  0  0  0  0  0  0  0  0  0  0
  -1.2450    2.1181   -0.0004 O   0  0  0  0  0  0  0  0  0  0  0  0
  -2.4055   -0.9868   -0.0003 H   0  0  0  0  0  0  0  0  0  0  0  0
  -3.6452    1.8048   -0.0003 H   0  0  0  0  0  0  0  0  0  0  0  0
  -5.9832    2.0443    0.0012 H   0  0  0  0  0  0  0  0  0  0  0  0
  -8.2770    1.1426    0.0017 H   0  0  0  0  0  0  0  0  0  0  0  0
  -8.8719   -1.4800    0.0006 O   0  0  0  0  0  0  0  0  0  0  0  0
  -9.5402   -0.7865    0.0010 H   0  0  0  0  0  0  0  0  0  0  0  0
  -6.7096   -2.8483   -0.0011 H   0  0  0  0  0  0  0  0  0  0  0  0
  -4.4117   -1.9527   -0.0015 H   0  0  0  0  0  0  0  0  0  0  0  0
  1  2  2  0  0  0  0
  1  6  1  0  0  0  0
```

```
2 3 1 0 0 0 0
3 36 1 0 0 0 0
3 4 2 0 0 0 0
4 35 1 0 0 0 0
5 4 1 0 0 0 0
5 34 1 0 0 0 0
6 5 2 0 0 0 0
7 33 1 0 0 0 0
7 6 1 0 0 0 0
8 32 1 0 0 0 0
8 7 2 0 0 0 0
9 10 1 0 0 0 0
9 31 2 0 0 0 0
9 8 1 0 0 0 0
10 11 2 0 0 0 0
10 30 1 0 0 0 0
11 28 1 0 0 0 0
11 12 1 0 0 0 0
12 13 2 0 0 0 0
12 27 1 0 0 0 0
13 14 1 0 0 0 0
14 15 2 0 0 0 0
15 25 1 0 0 0 0
15 16 1 0 0 0 0
16 24 1 0 0 0 0
17 22 1 0 0 0 0
17 16 2 0 0 0 0
18 17 1 0 0 0 0
19 18 2 0 0 0 0
19 14 1 0 0 0 0
20 19 1 0 0 0 0
21 18 1 0 0 0 0
22 23 1 0 0 0 0
26 13 1 0 0 0 0
29 28 1 0 0 0 0
36 37 1 0 0 0 0
38 2 1 0 0 0 0
39 1 1 0 0 0 0
M END
```

3B: CY S_0

```
49 51 0 0 0 0 0 0 0 0999 V2000
 1.2574 0.8426 0.6088 C 0 0 0 0 0 0 0 0 0 0 0 0
 -0.0002 1.1189 1.4339 C 0 0 0 0 0 0 0 0 0 0 0 0
 -0.0002 2.1570 1.7804 H 0 0 0 0 0 0 0 0 0 0 0 0
 -1.2575 0.8427 0.6084 C 0 0 0 0 0 0 0 0 0 0 0 0
 -1.2948 -0.5801 0.0960 C 0 0 0 0 0 0 0 0 0 0 0 0
 -2.4196 -1.2882 -0.1598 C 0 0 0 0 0 0 0 0 0 0 0 0
 -2.2350 -2.3230 -0.4366 H 0 0 0 0 0 0 0 0 0 0 0 0
```

```
-3.8300 -0.9223 -0.1036 C 0 0 0 0 0 0 0 0 0 0 0 0
-4.7696 -1.9599 0.0158 C 0 0 0 0 0 0 0 0 0 0 0 0
-6.1346 -1.7058 0.0797 C 0 0 0 0 0 0 0 0 0 0 0 0
-6.6022 -0.4011 -0.0011 C 0 0 0 0 0 0 0 0 0 0 0 0
-5.6795 0.6536 -0.1537 C 0 0 0 0 0 0 0 0 0 0 0 0
-4.3187 0.4009 -0.2011 C 0 0 0 0 0 0 0 0 0 0 0 0
-3.6315 1.2176 -0.3556 H 0 0 0 0 0 0 0 0 0 0 0 0
-6.2763 1.8874 -0.2528 O 0 0 0 0 0 0 0 0 0 0 0 0
-5.4503 3.0323 -0.4190 C 0 0 0 0 0 0 0 0 0 0 0 0
-4.7671 3.1537 0.4283 H 0 0 0 0 0 0 0 0 0 0 0 0
-4.8743 2.9737 -1.3485 H 0 0 0 0 0 0 0 0 0 0 0 0
-6.1248 3.8855 -0.4650 H 0 0 0 0 0 0 0 0 0 0 0 0
-7.9327 -0.1423 0.0487 O 0 0 0 0 0 0 0 0 0 0 0 0
-8.0417 0.8144 -0.0410 H 0 0 0 0 0 0 0 0 0 0 0 0
-6.8544 -2.5087 0.1824 H 0 0 0 0 0 0 0 0 0 0 0 0
-4.4169 -2.9834 0.0682 H 0 0 0 0 0 0 0 0 0 0 0 0
-0.0000 -1.3163 -0.1200 C 0 0 0 0 0 0 0 0 0 0 0 0
1.2947 -0.5800 0.0960 C 0 0 0 0 0 0 0 0 0 0 0 0
2.4195 -1.2881 -0.1602 C 0 0 0 0 0 0 0 0 0 0 0 0
3.8300 -0.9222 -0.1041 C 0 0 0 0 0 0 0 0 0 0 0 0
4.7695 -1.9599 0.0139 C 0 0 0 0 0 0 0 0 0 0 0 0
6.1345 -1.7061 0.0775 C 0 0 0 0 0 0 0 0 0 0 0 0
6.6022 -0.4013 -0.0020 C 0 0 0 0 0 0 0 0 0 0 0 0
5.6795 0.6536 -0.1533 C 0 0 0 0 0 0 0 0 0 0 0 0
4.3188 0.4011 -0.2004 C 0 0 0 0 0 0 0 0 0 0 0 0
3.6315 1.2181 -0.3538 H 0 0 0 0 0 0 0 0 0 0 0 0
6.2764 1.8875 -0.2512 O 0 0 0 0 0 0 0 0 0 0 0 0
5.4504 3.0327 -0.4154 C 0 0 0 0 0 0 0 0 0 0 0 0
4.8739 2.9754 -1.3448 H 0 0 0 0 0 0 0 0 0 0 0 0
4.7677 3.1529 0.4324 H 0 0 0 0 0 0 0 0 0 0 0 0
6.1250 3.8859 -0.4606 H 0 0 0 0 0 0 0 0 0 0 0 0
7.9328 -0.1426 0.0475 O 0 0 0 0 0 0 0 0 0 0 0 0
8.0418 0.8142 -0.0411 H 0 0 0 0 0 0 0 0 0 0 0 0
6.8543 -2.5091 0.1791 H 0 0 0 0 0 0 0 0 0 0 0 0
4.4168 -2.9834 0.0654 H 0 0 0 0 0 0 0 0 0 0 0 0
2.2349 -2.3227 -0.4373 H 0 0 0 0 0 0 0 0 0 0 0 0
-0.0000 -2.4860 -0.4866 O 0 0 0 0 0 0 0 0 0 0 0 0
-1.2849 1.5533 -0.2317 H 0 0 0 0 0 0 0 0 0 0 0 0
-2.1487 1.0397 1.2102 H 0 0 0 0 0 0 0 0 0 0 0 0
-0.0004 0.4836 2.3269 H 0 0 0 0 0 0 0 0 0 0 0 0
2.1484 1.0392 1.2111 H 0 0 0 0 0 0 0 0 0 0 0 0
1.2853 1.5534 -0.2310 H 0 0 0 0 0 0 0 0 0 0 0 0
1 48 1 0 0 0 0
1 2 1 0 0 0 0
2 3 1 0 0 0 0
2 47 1 0 0 0 0
4 46 1 0 0 0 0
4 2 1 0 0 0 0
5 4 1 0 0 0 0
6 8 1 0 0 0 0
```

```
  6  5  2  0  0  0  0
  7  6  1  0  0  0  0
  8  9  2  0  0  0  0
  9 23  1  0  0  0  0
  9 10  1  0  0  0  0
 10 22  1  0  0  0  0
 11 20  1  0  0  0  0
 11 10  2  0  0  0  0
 12 11  1  0  0  0  0
 13 12  2  0  0  0  0
 13  8  1  0  0  0  0
 14 13  1  0  0  0  0
 15 12  1  0  0  0  0
 16 15  1  0  0  0  0
 16 17  1  0  0  0  0
 18 16  1  0  0  0  0
 19 16  1  0  0  0  0
 21 20  1  0  0  0  0
 24  5  1  0  0  0  0
 24 25  1  0  0  0  0
 25  1  1  0  0  0  0
 26 27  1  0  0  0  0
 26 25  2  0  0  0  0
 27 28  2  0  0  0  0
 28 42  1  0  0  0  0
 28 29  1  0  0  0  0
 29 41  1  0  0  0  0
 30 39  1  0  0  0  0
 30 29  2  0  0  0  0
 31 30  1  0  0  0  0
 32 31  2  0  0  0  0
 32 27  1  0  0  0  0
 33 32  1  0  0  0  0
 34 31  1  0  0  0  0
 35 34  1  0  0  0  0
 35 37  1  0  0  0  0
 36 35  1  0  0  0  0
 38 35  1  0  0  0  0
 40 39  1  0  0  0  0
 43 26  1  0  0  0  0
 44 24  2  0  0  0  0
 45  4  1  0  0  0  0
 49  1  1  0  0  0  0
M  END
```

3B': O-CY S_0

```
 49 51  0  0  0  0  0  0  0  0999 V2000
    1.2573    0.8500    0.6012 C   0  0  0  0  0  0  0  0  0  0  0  0
   -0.0003    1.1258    1.4263 C   0  0  0  0  0  0  0  0  0  0  0  0
```

-0.0004 2.1638 1.7729 H 0 0 0 0 0 0 0 0 0 0 0 0 0
-1.2576 0.8500 0.6007 C 0 0 0 0 0 0 0 0 0 0 0 0 0
-1.2951 -0.5726 0.0880 C 0 0 0 0 0 0 0 0 0 0 0 0 0
-2.4192 -1.2812 -0.1680 C 0 0 0 0 0 0 0 0 0 0 0 0 0
-2.2341 -2.3154 -0.4463 H 0 0 0 0 0 0 0 0 0 0 0 0 0
-3.8305 -0.9165 -0.1087 C 0 0 0 0 0 0 0 0 0 0 0 0 0
-4.7659 -1.9531 0.0133 C 0 0 0 0 0 0 0 0 0 0 0 0 0
-6.1288 -1.6846 0.0828 C 0 0 0 0 0 0 0 0 0 0 0 0 0
-6.6031 -0.3805 0.0057 C 0 0 0 0 0 0 0 0 0 0 0 0 0
-5.6831 0.6804 -0.1530 C 0 0 0 0 0 0 0 0 0 0 0 0 0
-4.3228 0.4050 -0.2039 C 0 0 0 0 0 0 0 0 0 0 0 0 0
-3.6320 1.2174 -0.3615 H 0 0 0 0 0 0 0 0 0 0 0 0 0
-6.2375 1.9190 -0.2524 O 0 0 0 0 0 0 0 0 0 0 0 0 0
-5.3739 3.0322 -0.4217 C 0 0 0 0 0 0 0 0 0 0 0 0 0
-4.6832 3.1383 0.4228 H 0 0 0 0 0 0 0 0 0 0 0 0 0
-4.8003 2.9596 -1.3528 H 0 0 0 0 0 0 0 0 0 0 0 0 0
-6.0232 3.9049 -0.4670 H 0 0 0 0 0 0 0 0 0 0 0 0 0
-7.9233 -0.0572 0.0582 O 0 0 0 0 0 0 0 0 0 0 0 0 0
-8.4361 -0.8661 0.1587 H 0 0 0 0 0 0 0 0 0 0 0 0 0
-6.8388 -2.4999 0.1900 H 0 0 0 0 0 0 0 0 0 0 0 0 0
-4.4197 -2.9786 0.0650 H 0 0 0 0 0 0 0 0 0 0 0 0 0
-0.0000 -1.3090 -0.1280 C 0 0 0 0 0 0 0 0 0 0 0 0 0
1.2949 -0.5724 0.0880 C 0 0 0 0 0 0 0 0 0 0 0 0 0
2.4191 -1.2808 -0.1686 C 0 0 0 0 0 0 0 0 0 0 0 0 0
3.8304 -0.9163 -0.1096 C 0 0 0 0 0 0 0 0 0 0 0 0 0
4.7658 -1.9532 0.0102 C 0 0 0 0 0 0 0 0 0 0 0 0 0
6.1287 -1.6851 0.0792 C 0 0 0 0 0 0 0 0 0 0 0 0 0
6.6032 -0.3809 0.0041 C 0 0 0 0 0 0 0 0 0 0 0 0 0
5.6832 0.6805 -0.1521 C 0 0 0 0 0 0 0 0 0 0 0 0 0
4.3229 0.4054 -0.2028 C 0 0 0 0 0 0 0 0 0 0 0 0 0
3.6321 1.2182 -0.3584 H 0 0 0 0 0 0 0 0 0 0 0 0 0
6.2378 1.9191 -0.2497 O 0 0 0 0 0 0 0 0 0 0 0 0 0
5.3743 3.0329 -0.4156 C 0 0 0 0 0 0 0 0 0 0 0 0 0
4.7998 2.9626 -1.3464 H 0 0 0 0 0 0 0 0 0 0 0 0 0
4.6843 3.1371 0.4298 H 0 0 0 0 0 0 0 0 0 0 0 0 0
6.0236 3.9055 -0.4595 H 0 0 0 0 0 0 0 0 0 0 0 0 0
7.9235 -0.0578 0.0565 O 0 0 0 0 0 0 0 0 0 0 0 0 0
8.4362 -0.8670 0.1546 H 0 0 0 0 0 0 0 0 0 0 0 0 0
6.8386 -2.5006 0.1847 H 0 0 0 0 0 0 0 0 0 0 0 0 0
4.4194 -2.9787 0.0603 H 0 0 0 0 0 0 0 0 0 0 0 0 0
2.2339 -2.3149 -0.4474 H 0 0 0 0 0 0 0 0 0 0 0 0 0
0.0000 -2.4791 -0.4937 O 0 0 0 0 0 0 0 0 0 0 0 0 0
-1.2845 1.5601 -0.2396 H 0 0 0 0 0 0 0 0 0 0 0 0 0
-2.1491 1.0475 1.2016 H 0 0 0 0 0 0 0 0 0 0 0 0 0
-0.0005 0.4905 2.3194 H 0 0 0 0 0 0 0 0 0 0 0 0 0
2.1484 1.0473 1.2025 H 0 0 0 0 0 0 0 0 0 0 0 0 0
1.2846 1.5602 -0.2389 H 0 0 0 0 0 0 0 0 0 0 0 0 0
1 48 1 0 0 0 0
1 2 1 0 0 0 0
2 3 1 0 0 0 0

```
  2 47  1  0  0  0  0
  4 46  1  0  0  0  0
  4  2  1  0  0  0  0
  5  4  1  0  0  0  0
  6  8  1  0  0  0  0
  6  5  2  0  0  0  0
  7  6  1  0  0  0  0
  8  9  2  0  0  0  0
  9 23  1  0  0  0  0
  9 10  1  0  0  0  0
 10 22  1  0  0  0  0
 11 20  1  0  0  0  0
 11 10  2  0  0  0  0
 12 11  1  0  0  0  0
 13 12  2  0  0  0  0
 13  8  1  0  0  0  0
 14 13  1  0  0  0  0
 15 12  1  0  0  0  0
 16 15  1  0  0  0  0
 16 17  1  0  0  0  0
 18 16  1  0  0  0  0
 19 16  1  0  0  0  0
 20 21  1  0  0  0  0
 24  5  1  0  0  0  0
 24 25  1  0  0  0  0
 25  1  1  0  0  0  0
 26 27  1  0  0  0  0
 26 25  2  0  0  0  0
 27 28  2  0  0  0  0
 28 42  1  0  0  0  0
 28 29  1  0  0  0  0
 29 41  1  0  0  0  0
 30 39  1  0  0  0  0
 30 29  2  0  0  0  0
 31 30  1  0  0  0  0
 32 31  2  0  0  0  0
 32 27  1  0  0  0  0
 33 32  1  0  0  0  0
 34 31  1  0  0  0  0
 35 34  1  0  0  0  0
 35 37  1  0  0  0  0
 36 35  1  0  0  0  0
 38 35  1  0  0  0  0
 39 40  1  0  0  0  0
 43 26  1  0  0  0  0
 44 24  2  0  0  0  0
 45  4  1  0  0  0  0
 49  1  1  0  0  0  0
M  END
```

1A: CCE-Curc S_1

```
47 48 0 0 0 0 0 0 0 0999 V2000
   8.5487  -2.2371   0.0001 C  0 0 0 0 0 0 0 0 0 0 0 0 0
   8.5891  -0.8053   0.0002 O  0 0 0 0 0 0 0 0 0 0 0 0 0
   7.4220  -0.1175   0.0001 C  0 0 0 0 0 0 0 0 0 0 0 0 0
   7.6107   1.2999   0.0002 C  0 0 0 0 0 0 0 0 0 0 0 0 0
   6.4952   2.1489   0.0001 C  0 0 0 0 0 0 0 0 0 0 0 0 0
   5.2253   1.6091   0.0000 C  0 0 0 0 0 0 0 0 0 0 0 0 0
   5.0124   0.1950  -0.0000 C  0 0 0 0 0 0 0 0 0 0 0 0 0
   6.1438  -0.6507   0.0000 C  0 0 0 0 0 0 0 0 0 0 0 0 0
   5.9918  -1.7231  -0.0000 H  0 0 0 0 0 0 0 0 0 0 0 0 0
   3.6940  -0.4098  -0.0001 C  0 0 0 0 0 0 0 0 0 0 0 0 0
   2.5058   0.2396  -0.0002 C  0 0 0 0 0 0 0 0 0 0 0 0 0
   1.2134  -0.5251  -0.0003 C  0 0 0 0 0 0 0 0 0 0 0 0 0
   0.0289   0.2030  -0.0003 C  0 0 0 0 0 0 0 0 0 0 0 0 0
  -1.2418  -0.4691  -0.0002 C  0 0 0 0 0 0 0 0 0 0 0 0 0
  -2.4725   0.2075  -0.0001 C  0 0 0 0 0 0 0 0 0 0 0 0 0
  -3.7094  -0.4325  -0.0001 C  0 0 0 0 0 0 0 0 0 0 0 0 0
  -5.0009   0.1799  -0.0000 C  0 0 0 0 0 0 0 0 0 0 0 0 0
  -5.2121   1.5855  -0.0000 C  0 0 0 0 0 0 0 0 0 0 0 0 0
  -6.4904   2.1275   0.0001 C  0 0 0 0 0 0 0 0 0 0 0 0 0
  -7.6128   1.2993   0.0001 C  0 0 0 0 0 0 0 0 0 0 0 0 0
  -7.4314  -0.1018   0.0001 C  0 0 0 0 0 0 0 0 0 0 0 0 0
  -6.1620  -0.6496   0.0000 C  0 0 0 0 0 0 0 0 0 0 0 0 0
  -6.0268  -1.7255  -0.0000 H  0 0 0 0 0 0 0 0 0 0 0 0 0
  -8.6189  -0.8046   0.0001 O  0 0 0 0 0 0 0 0 0 0 0 0 0
  -8.5533  -2.2208   0.0001 C  0 0 0 0 0 0 0 0 0 0 0 0 0
  -9.5853  -2.5755   0.0001 H  0 0 0 0 0 0 0 0 0 0 0 0 0
  -8.0392  -2.5974  -0.8930 H  0 0 0 0 0 0 0 0 0 0 0 0 0
  -8.0391  -2.5975   0.8931 H  0 0 0 0 0 0 0 0 0 0 0 0 0
  -8.8701   1.8323   0.0002 O  0 0 0 0 0 0 0 0 0 0 0 0 0
  -9.4863   1.0833   0.0002 H  0 0 0 0 0 0 0 0 0 0 0 0 0
  -6.6440   3.2022   0.0001 H  0 0 0 0 0 0 0 0 0 0 0 0 0
  -4.3597   2.2560  -0.0000 H  0 0 0 0 0 0 0 0 0 0 0 0 0
  -3.6903  -1.5193  -0.0001 H  0 0 0 0 0 0 0 0 0 0 0 0 0
  -2.4134   1.2916  -0.0001 H  0 0 0 0 0 0 0 0 0 0 0 0 0
  -1.2300  -1.8067  -0.0001 O  0 0 0 0 0 0 0 0 0 0 0 0 0
  -0.2524  -2.0781  -0.0001 H  0 0 0 0 0 0 0 0 0 0 0 0 0
   0.0449   1.2865  -0.0002 H  0 0 0 0 0 0 0 0 0 0 0 0 0
   1.3007  -1.8035  -0.0002 O  0 0 0 0 0 0 0 0 0 0 0 0 0
   2.4429   1.3256  -0.0001 H  0 0 0 0 0 0 0 0 0 0 0 0 0
   3.6359  -1.4957  -0.0002 H  0 0 0 0 0 0 0 0 0 0 0 0 0
   4.3702   2.2740  -0.0000 H  0 0 0 0 0 0 0 0 0 0 0 0 0
   6.6604   3.2203   0.0002 H  0 0 0 0 0 0 0 0 0 0 0 0 0
   8.8486   1.8117   0.0003 O  0 0 0 0 0 0 0 0 0 0 0 0 0
   9.4826   1.0735   0.0003 H  0 0 0 0 0 0 0 0 0 0 0 0 0
   8.0420  -2.6082  -0.8961 H  0 0 0 0 0 0 0 0 0 0 0 0 0
   9.5878  -2.5633   0.0002 H  0 0 0 0 0 0 0 0 0 0 0 0 0
   8.0418  -2.6082   0.8962 H  0 0 0 0 0 0 0 0 0 0 0 0 0
```

1 46 1 0 0 0 0
1 2 1 0 0 0 0
1 47 1 0 0 0 0
3 2 1 0 0 0 0
3 4 2 0 0 0 0
4 43 1 0 0 0 0
5 4 1 0 0 0 0
5 42 1 0 0 0 0
6 5 2 0 0 0 0
7 8 2 0 0 0 0
7 6 1 0 0 0 0
8 3 1 0 0 0 0
9 8 1 0 0 0 0
10 7 1 0 0 0 0
11 39 1 0 0 0 0
11 10 2 0 0 0 0
12 13 1 0 0 0 0
12 38 2 0 0 0 0
12 11 1 0 0 0 0
13 37 1 0 0 0 0
13 14 1 0 0 0 0
14 15 2 0 0 0 0
14 35 1 0 0 0 0
15 34 1 0 0 0 0
15 16 1 0 0 0 0
16 17 1 0 0 0 0
17 18 2 0 0 0 0
17 22 1 0 0 0 0
18 19 1 0 0 0 0
19 31 1 0 0 0 0
19 20 2 0 0 0 0
20 29 1 0 0 0 0
21 20 1 0 0 0 0
21 24 1 0 0 0 0
22 21 2 0 0 0 0
23 22 1 0 0 0 0
25 24 1 0 0 0 0
25 26 1 0 0 0 0
25 28 1 0 0 0 0
27 25 1 0 0 0 0
29 30 1 0 0 0 0
32 18 1 0 0 0 0
33 16 1 0 0 0 0
35 36 1 0 0 0 0
40 10 1 0 0 0 0
41 6 1 0 0 0 0
43 44 1 0 0 0 0
45 1 1 0 0 0 0
M END

2A: CCE-Curc-Conf S_0

```
47 48 0 0 0 0 0 0 0 0999 V2000
-5.8461 3.4550 0.0013 C 0 0 0 0 0 0 0 0 0 0 0 0 0
-6.8772 2.4753 0.0005 O 0 0 0 0 0 0 0 0 0 0 0 0 0
-6.5235 1.1493 0.0001 C 0 0 0 0 0 0 0 0 0 0 0 0 0
-7.6357 0.2767 -0.0003 C 0 0 0 0 0 0 0 0 0 0 0 0 0
-7.4261 -1.0944 -0.0007 C 0 0 0 0 0 0 0 0 0 0 0 0 0
-6.1287 -1.5984 -0.0006 C 0 0 0 0 0 0 0 0 0 0 0 0 0
-5.0141 -0.7491 -0.0002 C 0 0 0 0 0 0 0 0 0 0 0 0 0
-5.2368 0.6463 0.0002 C 0 0 0 0 0 0 0 0 0 0 0 0 0
-4.3927 1.3220 0.0006 H 0 0 0 0 0 0 0 0 0 0 0 0 0
-3.6831 -1.3371 -0.0002 C 0 0 0 0 0 0 0 0 0 0 0 0 0
-2.4892 -0.7139 -0.0000 C 0 0 0 0 0 0 0 0 0 0 0 0 0
-1.2312 -1.4790 0.0000 C 0 0 0 0 0 0 0 0 0 0 0 0 0
0.0074 -0.7451 0.0001 C 0 0 0 0 0 0 0 0 0 0 0 0 0
1.2194 -1.4087 0.0001 C 0 0 0 0 0 0 0 0 0 0 0 0 0
2.4863 -0.7027 0.0001 C 0 0 0 0 0 0 0 0 0 0 0 0 0
3.6770 -1.3374 0.0002 C 0 0 0 0 0 0 0 0 0 0 0 0 0
5.0069 -0.7475 0.0002 C 0 0 0 0 0 0 0 0 0 0 0 0 0
5.2293 0.6482 -0.0002 C 0 0 0 0 0 0 0 0 0 0 0 0 0
6.5157 1.1519 -0.0002 C 0 0 0 0 0 0 0 0 0 0 0 0 0
7.6287 0.2802 0.0003 C 0 0 0 0 0 0 0 0 0 0 0 0 0
7.4195 -1.0910 0.0007 C 0 0 0 0 0 0 0 0 0 0 0 0 0
6.1226 -1.5958 0.0006 C 0 0 0 0 0 0 0 0 0 0 0 0 0
5.9696 -2.6689 0.0009 H 0 0 0 0 0 0 0 0 0 0 0 0 0
8.2813 -1.7469 0.0010 H 0 0 0 0 0 0 0 0 0 0 0 0 0
8.8862 0.7852 0.0003 O 0 0 0 0 0 0 0 0 0 0 0 0 0
8.8093 1.7494 -0.0000 H 0 0 0 0 0 0 0 0 0 0 0 0 0
6.8683 2.4779 -0.0006 O 0 0 0 0 0 0 0 0 0 0 0 0 0
5.8369 3.4574 -0.0013 C 0 0 0 0 0 0 0 0 0 0 0 0 0
5.2117 3.3719 -0.8962 H 0 0 0 0 0 0 0 0 0 0 0 0 0
5.2113 3.3727 0.8935 H 0 0 0 0 0 0 0 0 0 0 0 0 0
6.3395 4.4229 -0.0016 H 0 0 0 0 0 0 0 0 0 0 0 0 0
4.3861 1.3250 -0.0006 H 0 0 0 0 0 0 0 0 0 0 0 0 0
3.6488 -2.4230 0.0003 H 0 0 0 0 0 0 0 0 0 0 0 0 0
2.4164 0.3793 0.0001 H 0 0 0 0 0 0 0 0 0 0 0 0 0
1.2745 -2.7355 0.0001 O 0 0 0 0 0 0 0 0 0 0 0 0 0
0.3129 -3.0425 0.0001 H 0 0 0 0 0 0 0 0 0 0 0 0 0
-0.0028 0.3362 0.0001 H 0 0 0 0 0 0 0 0 0 0 0 0 0
-1.2436 -2.7361 -0.0001 O 0 0 0 0 0 0 0 0 0 0 0 0 0
-2.4041 0.3680 0.0001 H 0 0 0 0 0 0 0 0 0 0 0 0 0
-3.6457 -2.4238 -0.0003 H 0 0 0 0 0 0 0 0 0 0 0 0 0
-5.9740 -2.6712 -0.0010 H 0 0 0 0 0 0 0 0 0 0 0 0 0
-8.2877 -1.7507 -0.0011 H 0 0 0 0 0 0 0 0 0 0 0 0 0
-8.8936 0.7812 -0.0004 O 0 0 0 0 0 0 0 0 0 0 0 0 0
-8.8168 1.7454 -0.0000 H 0 0 0 0 0 0 0 0 0 0 0 0 0
-6.3489 4.4204 0.0017 H 0 0 0 0 0 0 0 0 0 0 0 0 0
-5.2209 3.3694 0.8962 H 0 0 0 0 0 0 0 0 0 0 0 0 0
-5.2203 3.3704 -0.8933 H 0 0 0 0 0 0 0 0 0 0 0 0 0
1 45 1 0 0 0 0
```

1 46 1 0 0 0 0
2 1 1 0 0 0 0
3 8 1 0 0 0 0
3 2 1 0 0 0 0
4 3 2 0 0 0 0
5 6 2 0 0 0 0
5 4 1 0 0 0 0
6 7 1 0 0 0 0
7 10 1 0 0 0 0
7 8 2 0 0 0 0
8 9 1 0 0 0 0
10 11 2 0 0 0 0
11 12 1 0 0 0 0
11 39 1 0 0 0 0
12 13 1 0 0 0 0
13 14 2 0 0 0 0
13 37 1 0 0 0 0
14 35 1 0 0 0 0
14 15 1 0 0 0 0
15 16 2 0 0 0 0
16 17 1 0 0 0 0
16 33 1 0 0 0 0
17 22 2 0 0 0 0
18 19 2 0 0 0 0
18 17 1 0 0 0 0
19 20 1 0 0 0 0
20 25 1 0 0 0 0
20 21 2 0 0 0 0
21 24 1 0 0 0 0
22 21 1 0 0 0 0
22 23 1 0 0 0 0
26 25 1 0 0 0 0
27 19 1 0 0 0 0
28 27 1 0 0 0 0
28 30 1 0 0 0 0
29 28 1 0 0 0 0
31 28 1 0 0 0 0
32 18 1 0 0 0 0
34 15 1 0 0 0 0
35 36 1 0 0 0 0
38 12 2 0 0 0 0
40 10 1 0 0 0 0
41 6 1 0 0 0 0
42 5 1 0 0 0 0
43 4 1 0 0 0 0
43 44 1 0 0 0 0
47 1 1 0 0 0 0
M END

2A: CCE-Curc-Conf S_1

```
47 48 0 0 0 0 0 0 0 0999 V2000
5.6899 3.4512 -0.0005 C 0 0 0 0 0 0 0 0 0 0 0 0
6.7612 2.5216 -0.0003 O 0 0 0 0 0 0 0 0 0 0 0 0
6.4566 1.1781 -0.0001 C 0 0 0 0 0 0 0 0 0 0 0 0
7.6049 0.3515 0.0000 C 0 0 0 0 0 0 0 0 0 0 0 0
7.4462 -1.0360 0.0002 C 0 0 0 0 0 0 0 0 0 0 0 0
6.1750 -1.5897 0.0002 C 0 0 0 0 0 0 0 0 0 0 0 0
5.0053 -0.7835 0.0001 C 0 0 0 0 0 0 0 0 0 0 0 0
5.1862 0.6323 -0.0001 C 0 0 0 0 0 0 0 0 0 0 0 0
4.3181 1.2792 -0.0002 H 0 0 0 0 0 0 0 0 0 0 0 0
3.7189 -1.4086 0.0002 C 0 0 0 0 0 0 0 0 0 0 0 0
2.4763 -0.7937 0.0001 C 0 0 0 0 0 0 0 0 0 0 0 0
1.2581 -1.5347 0.0002 C 0 0 0 0 0 0 0 0 0 0 0 0
-0.0164 -0.7985 0.0001 C 0 0 0 0 0 0 0 0 0 0 0 0
-1.2264 -1.4793 0.0000 C 0 0 0 0 0 0 0 0 0 0 0 0
-2.4787 -0.7685 -0.0000 C 0 0 0 0 0 0 0 0 0 0 0 0
-3.6979 -1.3950 -0.0001 C 0 0 0 0 0 0 0 0 0 0 0 0
-4.9924 -0.7640 -0.0001 C 0 0 0 0 0 0 0 0 0 0 0 0
-5.1731 0.6464 0.0001 C 0 0 0 0 0 0 0 0 0 0 0 0
-6.4445 1.1868 0.0001 C 0 0 0 0 0 0 0 0 0 0 0 0
-7.5908 0.3431 -0.0002 C 0 0 0 0 0 0 0 0 0 0 0 0
-7.4276 -1.0460 -0.0004 C 0 0 0 0 0 0 0 0 0 0 0 0
-6.1531 -1.5854 -0.0003 C 0 0 0 0 0 0 0 0 0 0 0 0
-6.0266 -2.6633 -0.0005 H 0 0 0 0 0 0 0 0 0 0 0 0
-8.3125 -1.6728 -0.0005 H 0 0 0 0 0 0 0 0 0 0 0 0
-8.8248 0.8821 -0.0002 O 0 0 0 0 0 0 0 0 0 0 0 0
-8.7240 1.8491 -0.0000 H 0 0 0 0 0 0 0 0 0 0 0 0
-6.7651 2.5142 0.0003 O 0 0 0 0 0 0 0 0 0 0 0 0
-5.7089 3.4716 0.0005 C 0 0 0 0 0 0 0 0 0 0 0 0
-5.0860 3.3680 0.8961 H 0 0 0 0 0 0 0 0 0 0 0 0
-5.0858 3.3684 -0.8949 H 0 0 0 0 0 0 0 0 0 0 0 0
-6.1893 4.4499 0.0007 H 0 0 0 0 0 0 0 0 0 0 0 0
-4.3088 1.2977 0.0003 H 0 0 0 0 0 0 0 0 0 0 0 0
-3.6857 -2.4814 -0.0001 H 0 0 0 0 0 0 0 0 0 0 0 0
-2.4034 0.3153 0.0000 H 0 0 0 0 0 0 0 0 0 0 0 0
-1.2702 -2.8127 -0.0000 O 0 0 0 0 0 0 0 0 0 0 0 0
-0.2687 -3.0832 -0.0000 H 0 0 0 0 0 0 0 0 0 0 0 0
-0.0180 0.2859 0.0001 H 0 0 0 0 0 0 0 0 0 0 0 0
1.2066 -2.8133 -0.0000 O 0 0 0 0 0 0 0 0 0 0 0 0
2.3898 0.2896 0.0001 H 0 0 0 0 0 0 0 0 0 0 0 0
3.7078 -2.4960 0.0002 H 0 0 0 0 0 0 0 0 0 0 0 0
6.0619 -2.6698 0.0004 H 0 0 0 0 0 0 0 0 0 0 0 0
8.3347 -1.6590 0.0003 H 0 0 0 0 0 0 0 0 0 0 0 0
8.8488 0.9041 0.0000 O 0 0 0 0 0 0 0 0 0 0 0 0
8.7276 1.8669 -0.0001 H 0 0 0 0 0 0 0 0 0 0 0 0
6.1454 4.4426 -0.0006 H 0 0 0 0 0 0 0 0 0 0 0 0
5.0637 3.3380 -0.8942 H 0 0 0 0 0 0 0 0 0 0 0 0
5.0636 3.3382 0.8932 H 0 0 0 0 0 0 0 0 0 0 0 0
1 2 1 0 0 0 0
```

```
 1 47  1  0  0  0  0
  2  3  1  0  0  0  0
  3  8  2  0  0  0  0
  3  4  1  0  0  0  0
  4  5  2  0  0  0  0
  5  6  1  0  0  0  0
  5 42  1  0  0  0  0
  6 41  1  0  0  0  0
  7 10  1  0  0  0  0
  7  6  2  0  0  0  0
  8  7  1  0  0  0  0
  9  8  1  0  0  0  0
 10 40  1  0  0  0  0
 11 10  2  0  0  0  0
 11 12  1  0  0  0  0
 13 12  1  0  0  0  0
 14 13  2  0  0  0  0
 15 34  1  0  0  0  0
 15 14  1  0  0  0  0
 16 15  2  0  0  0  0
 17 16  1  0  0  0  0
 17 18  2  0  0  0  0
 18 32  1  0  0  0  0
 19 18  1  0  0  0  0
 19 27  1  0  0  0  0
 20 19  2  0  0  0  0
 21 22  2  0  0  0  0
 21 20  1  0  0  0  0
 22 17  1  0  0  0  0
 23 22  1  0  0  0  0
 24 21  1  0  0  0  0
 25 20  1  0  0  0  0
 25 26  1  0  0  0  0
 27 28  1  0  0  0  0
 28 31  1  0  0  0  0
 28 29  1  0  0  0  0
 30 28  1  0  0  0  0
 33 16  1  0  0  0  0
 35 14  1  0  0  0  0
 36 35  1  0  0  0  0
 37 13  1  0  0  0  0
 38 12  2  0  0  0  0
 39 11  1  0  0  0  0
 43  4  1  0  0  0  0
 44 43  1  0  0  0  0
 45  1  1  0  0  0  0
 46  1  1  0  0  0  0
M  END
```

3B: CY S_1

```
49 51  0  0  0  0  0  0  0  0999 V2000
    1.2610    0.8902    0.6198 C   0  0  0  0  0  0  0  0  0  0  0  0
   -0.0000    1.2125    1.4245 C   0  0  0  0  0  0  0  0  0  0  0  0
   -0.0000    2.2692    1.7155 H   0  0  0  0  0  0  0  0  0  0  0  0
   -1.2610    0.8902    0.6197 C   0  0  0  0  0  0  0  0  0  0  0  0
   -1.2683   -0.5416    0.1288 C   0  0  0  0  0  0  0  0  0  0  0  0
   -2.4087   -1.2962   -0.1032 C   0  0  0  0  0  0  0  0  0  0  0  0
   -2.2348   -2.3484   -0.3161 H   0  0  0  0  0  0  0  0  0  0  0  0
   -3.8081   -0.9383   -0.0722 C   0  0  0  0  0  0  0  0  0  0  0  0
   -4.7552   -1.9833    0.0358 C   0  0  0  0  0  0  0  0  0  0  0  0
   -6.1225   -1.7371    0.0661 C   0  0  0  0  0  0  0  0  0  0  0  0
   -6.5975   -0.4311   -0.0252 C   0  0  0  0  0  0  0  0  0  0  0  0
   -5.6745    0.6290   -0.1607 C   0  0  0  0  0  0  0  0  0  0  0  0
   -4.3101    0.3868   -0.1876 C   0  0  0  0  0  0  0  0  0  0  0  0
   -3.6268    1.2098   -0.3378 H   0  0  0  0  0  0  0  0  0  0  0  0
   -6.2778    1.8610   -0.2697 O   0  0  0  0  0  0  0  0  0  0  0  0
   -5.4518    3.0046   -0.4258 C   0  0  0  0  0  0  0  0  0  0  0  0
   -4.7756    3.1278    0.4292 H   0  0  0  0  0  0  0  0  0  0  0  0
   -4.8605    2.9471   -1.3479 H   0  0  0  0  0  0  0  0  0  0  0  0
   -6.1248    3.8612   -0.4811 H   0  0  0  0  0  0  0  0  0  0  0  0
   -7.9326   -0.1788    0.0014 O   0  0  0  0  0  0  0  0  0  0  0  0
   -8.0413    0.7810   -0.0894 H   0  0  0  0  0  0  0  0  0  0  0  0
   -6.8398   -2.5458    0.1577 H   0  0  0  0  0  0  0  0  0  0  0  0
   -4.3992   -3.0066    0.1070 H   0  0  0  0  0  0  0  0  0  0  0  0
   -0.0000   -1.1737   -0.0868 C   0  0  0  0  0  0  0  0  0  0  0  0
    1.2683   -0.5416    0.1288 C   0  0  0  0  0  0  0  0  0  0  0  0
    2.4087   -1.2961   -0.1032 C   0  0  0  0  0  0  0  0  0  0  0  0
    3.8081   -0.9383   -0.0723 C   0  0  0  0  0  0  0  0  0  0  0  0
    4.7552   -1.9833    0.0355 C   0  0  0  0  0  0  0  0  0  0  0  0
    6.1225   -1.7371    0.0658 C   0  0  0  0  0  0  0  0  0  0  0  0
    6.5975   -0.4311   -0.0253 C   0  0  0  0  0  0  0  0  0  0  0  0
    5.6745    0.6290   -0.1606 C   0  0  0  0  0  0  0  0  0  0  0  0
    4.3101    0.3868   -0.1875 C   0  0  0  0  0  0  0  0  0  0  0  0
    3.6268    1.2098   -0.3375 H   0  0  0  0  0  0  0  0  0  0  0  0
    6.2778    1.8610   -0.2695 O   0  0  0  0  0  0  0  0  0  0  0  0
    5.4518    3.0046   -0.4253 C   0  0  0  0  0  0  0  0  0  0  0  0
    4.8604    2.9474   -1.3473 H   0  0  0  0  0  0  0  0  0  0  0  0
    4.7757    3.1277    0.4298 H   0  0  0  0  0  0  0  0  0  0  0  0
    6.1248    3.8613   -0.4804 H   0  0  0  0  0  0  0  0  0  0  0  0
    7.9326   -0.1789    0.0012 O   0  0  0  0  0  0  0  0  0  0  0  0
    8.0413    0.7810   -0.0894 H   0  0  0  0  0  0  0  0  0  0  0  0
    6.8398   -2.5458    0.1571 H   0  0  0  0  0  0  0  0  0  0  0  0
    4.3991   -3.0066    0.1066 H   0  0  0  0  0  0  0  0  0  0  0  0
    2.2348   -2.3484   -0.3163 H   0  0  0  0  0  0  0  0  0  0  0  0
   -0.0000   -2.3975   -0.5735 O   0  0  0  0  0  0  0  0  0  0  0  0
   -1.3376    1.5925   -0.2251 H   0  0  0  0  0  0  0  0  0  0  0  0
   -2.1450    1.0542    1.2439 H   0  0  0  0  0  0  0  0  0  0  0  0
   -0.0001    0.6219    2.3483 H   0  0  0  0  0  0  0  0  0  0  0  0
    2.1449    1.0542    1.2441 H   0  0  0  0  0  0  0  0  0  0  0  0
```

1.3377 1.5925 -0.2250 H 0 0 0 0 0 0 0 0 0 0 0 0
1 48 1 0 0 0 0
1 2 1 0 0 0 0
2 3 1 0 0 0 0
2 47 1 0 0 0 0
4 46 1 0 0 0 0
4 2 1 0 0 0 0
5 4 1 0 0 0 0
6 8 1 0 0 0 0
6 5 2 0 0 0 0
7 6 1 0 0 0 0
8 9 2 0 0 0 0
9 10 1 0 0 0 0
9 23 1 0 0 0 0
10 22 1 0 0 0 0
11 20 1 0 0 0 0
11 10 2 0 0 0 0
12 11 1 0 0 0 0
13 12 2 0 0 0 0
13 8 1 0 0 0 0
14 13 1 0 0 0 0
15 12 1 0 0 0 0
16 15 1 0 0 0 0
16 17 1 0 0 0 0
18 16 1 0 0 0 0
19 16 1 0 0 0 0
21 20 1 0 0 0 0
24 25 2 0 0 0 0
24 5 1 0 0 0 0
25 1 1 0 0 0 0
26 27 1 0 0 0 0
26 25 1 0 0 0 0
27 28 2 0 0 0 0
28 29 1 0 0 0 0
28 42 1 0 0 0 0
29 41 1 0 0 0 0
30 39 1 0 0 0 0
30 29 2 0 0 0 0
31 30 1 0 0 0 0
32 31 2 0 0 0 0
32 27 1 0 0 0 0
33 32 1 0 0 0 0
34 31 1 0 0 0 0
35 34 1 0 0 0 0
35 37 1 0 0 0 0
36 35 1 0 0 0 0
38 35 1 0 0 0 0
40 39 1 0 0 0 0
43 26 1 0 0 0 0
44 24 1 0 0 0 0

45 4 1 0 0 0 0
49 1 1 0 0 0 0
M END

3B': O-CY S_1

49 51 0 0 0 0 0 0 0 0999 V2000
1.2614 0.9010 0.6106 C 0 0 0 0 0 0 0 0 0 0 0 0
0.0000 1.2252 1.4141 C 0 0 0 0 0 0 0 0 0 0 0 0
0.0000 2.2826 1.7022 H 0 0 0 0 0 0 0 0 0 0 0 0
-1.2613 0.9011 0.6105 C 0 0 0 0 0 0 0 0 0 0 0 0
-1.2685 -0.5319 0.1225 C 0 0 0 0 0 0 0 0 0 0 0 0
-2.4077 -1.2881 -0.1078 C 0 0 0 0 0 0 0 0 0 0 0 0
-2.2322 -2.3400 -0.3215 H 0 0 0 0 0 0 0 0 0 0 0 0
-3.8083 -0.9327 -0.0743 C 0 0 0 0 0 0 0 0 0 0 0 0
-4.7509 -1.9771 0.0360 C 0 0 0 0 0 0 0 0 0 0 0 0
-6.1165 -1.7174 0.0687 C 0 0 0 0 0 0 0 0 0 0 0 0
-6.5992 -0.4129 -0.0212 C 0 0 0 0 0 0 0 0 0 0 0 0
-5.6789 0.6539 -0.1613 C 0 0 0 0 0 0 0 0 0 0 0 0
-4.3149 0.3900 -0.1893 C 0 0 0 0 0 0 0 0 0 0 0 0
-3.6282 1.2088 -0.3424 H 0 0 0 0 0 0 0 0 0 0 0 0
-6.2411 1.8913 -0.2720 O 0 0 0 0 0 0 0 0 0 0 0 0
-5.3782 3.0050 -0.4239 C 0 0 0 0 0 0 0 0 0 0 0 0
-4.6980 3.1113 0.4314 H 0 0 0 0 0 0 0 0 0 0 0 0
-4.7851 2.9373 -1.3454 H 0 0 0 0 0 0 0 0 0 0 0 0
-6.0263 3.8808 -0.4785 H 0 0 0 0 0 0 0 0 0 0 0 0
-7.9245 -0.0964 0.0042 O 0 0 0 0 0 0 0 0 0 0 0 0
-8.4331 -0.9131 0.0954 H 0 0 0 0 0 0 0 0 0 0 0 0
-6.8244 -2.5384 0.1641 H 0 0 0 0 0 0 0 0 0 0 0 0
-4.4010 -3.0023 0.1077 H 0 0 0 0 0 0 0 0 0 0 0 0
0.0000 -1.1642 -0.0928 C 0 0 0 0 0 0 0 0 0 0 0 0
1.2685 -0.5319 0.1225 C 0 0 0 0 0 0 0 0 0 0 0 0
2.4077 -1.2880 -0.1081 C 0 0 0 0 0 0 0 0 0 0 0 0
3.8083 -0.9327 -0.0746 C 0 0 0 0 0 0 0 0 0 0 0 0
4.7509 -1.9771 0.0350 C 0 0 0 0 0 0 0 0 0 0 0 0
6.1165 -1.7175 0.0677 C 0 0 0 0 0 0 0 0 0 0 0 0
6.5992 -0.4129 -0.0216 C 0 0 0 0 0 0 0 0 0 0 0 0
5.6789 0.6539 -0.1611 C 0 0 0 0 0 0 0 0 0 0 0 0
4.3149 0.3900 -0.1890 C 0 0 0 0 0 0 0 0 0 0 0 0
3.6282 1.2090 -0.3416 H 0 0 0 0 0 0 0 0 0 0 0 0
6.2411 1.8914 -0.2712 O 0 0 0 0 0 0 0 0 0 0 0 0
5.3782 3.0052 -0.4223 C 0 0 0 0 0 0 0 0 0 0 0 0
4.7849 2.9380 -1.3437 H 0 0 0 0 0 0 0 0 0 0 0 0
4.6982 3.1110 0.4332 H 0 0 0 0 0 0 0 0 0 0 0 0
6.0263 3.8810 -0.4765 H 0 0 0 0 0 0 0 0 0 0 0 0
7.9245 -0.0965 0.0038 O 0 0 0 0 0 0 0 0 0 0 0 0
8.4331 -0.9133 0.0945 H 0 0 0 0 0 0 0 0 0 0 0 0
6.8244 -2.5385 0.1626 H 0 0 0 0 0 0 0 0 0 0 0 0
4.4010 -3.0024 0.1063 H 0 0 0 0 0 0 0 0 0 0 0 0
2.2322 -2.3399 -0.3220 H 0 0 0 0 0 0 0 0 0 0 0 0

```
-0.0001 -2.3886 -0.5777 O 0 0 0 0 0 0 0 0 0 0 0 0
-1.3386 1.6009 -0.2362 H 0 0 0 0 0 0 0 0 0 0 0 0
-2.1453 1.0669 1.2342 H 0 0 0 0 0 0 0 0 0 0 0 0
-0.0001 0.6372 2.3397 H 0 0 0 0 0 0 0 0 0 0 0 0
2.1453 1.0667 1.2344 H 0 0 0 0 0 0 0 0 0 0 0 0
1.3389 1.6009 -0.2360 H 0 0 0 0 0 0 0 0 0 0 0 0
1 48 1 0 0 0 0
1 2 1 0 0 0 0
2 3 1 0 0 0 0
2 47 1 0 0 0 0
4 46 1 0 0 0 0
4 2 1 0 0 0 0
5 4 1 0 0 0 0
6 8 1 0 0 0 0
6 5 2 0 0 0 0
7 6 1 0 0 0 0
8 9 2 0 0 0 0
9 10 1 0 0 0 0
9 23 1 0 0 0 0
10 22 1 0 0 0 0
11 20 1 0 0 0 0
11 10 2 0 0 0 0
12 11 1 0 0 0 0
13 12 2 0 0 0 0
13 8 1 0 0 0 0
14 13 1 0 0 0 0
15 12 1 0 0 0 0
16 15 1 0 0 0 0
16 17 1 0 0 0 0
18 16 1 0 0 0 0
19 16 1 0 0 0 0
20 21 1 0 0 0 0
24 25 2 0 0 0 0
24 5 1 0 0 0 0
25 1 1 0 0 0 0
26 27 1 0 0 0 0
26 25 1 0 0 0 0
27 28 2 0 0 0 0
28 29 1 0 0 0 0
28 42 1 0 0 0 0
29 41 1 0 0 0 0
30 39 1 0 0 0 0
30 29 2 0 0 0 0
31 30 1 0 0 0 0
32 31 2 0 0 0 0
32 27 1 0 0 0 0
33 32 1 0 0 0 0
34 31 1 0 0 0 0
35 34 1 0 0 0 0
35 37 1 0 0 0 0
```

```
36 35  1  0  0  0  0
38 35  1  0  0  0  0
39 40  1  0  0  0  0
43 26  1  0  0  0  0
44 24  1  0  0  0  0
45  4  1  0  0  0  0
49  1  1  0  0  0  0
M  END
```

S1. Conformational analysis of curcumin

First, the relative thermodynamic and kinetic stability of conformational isomers of enol curcumin was investigated. More specifically, two relaxed scans of the Potential Energy Surface (PES) of the compound were performed starting from the geometry of the Closed Cis isomer (compound 1A:CCE-Curc, Figure 2 in the main text).

In the first case we varied the dihedral angle between the planes containing the alkenyl moiety and the hydroxyl moiety of the keto-enol group, respectively, to generate the Open Cis Enol isomer (compound 4A:OCE-Curc) (Figure S1). In the second case instead, we modified the dihedral angle between the planes containing the carbonyl moiety and hydroxyl one of the same group to obtain the Trans Enol isomer (compound 5A:T-E-Curc) (Figure S2).

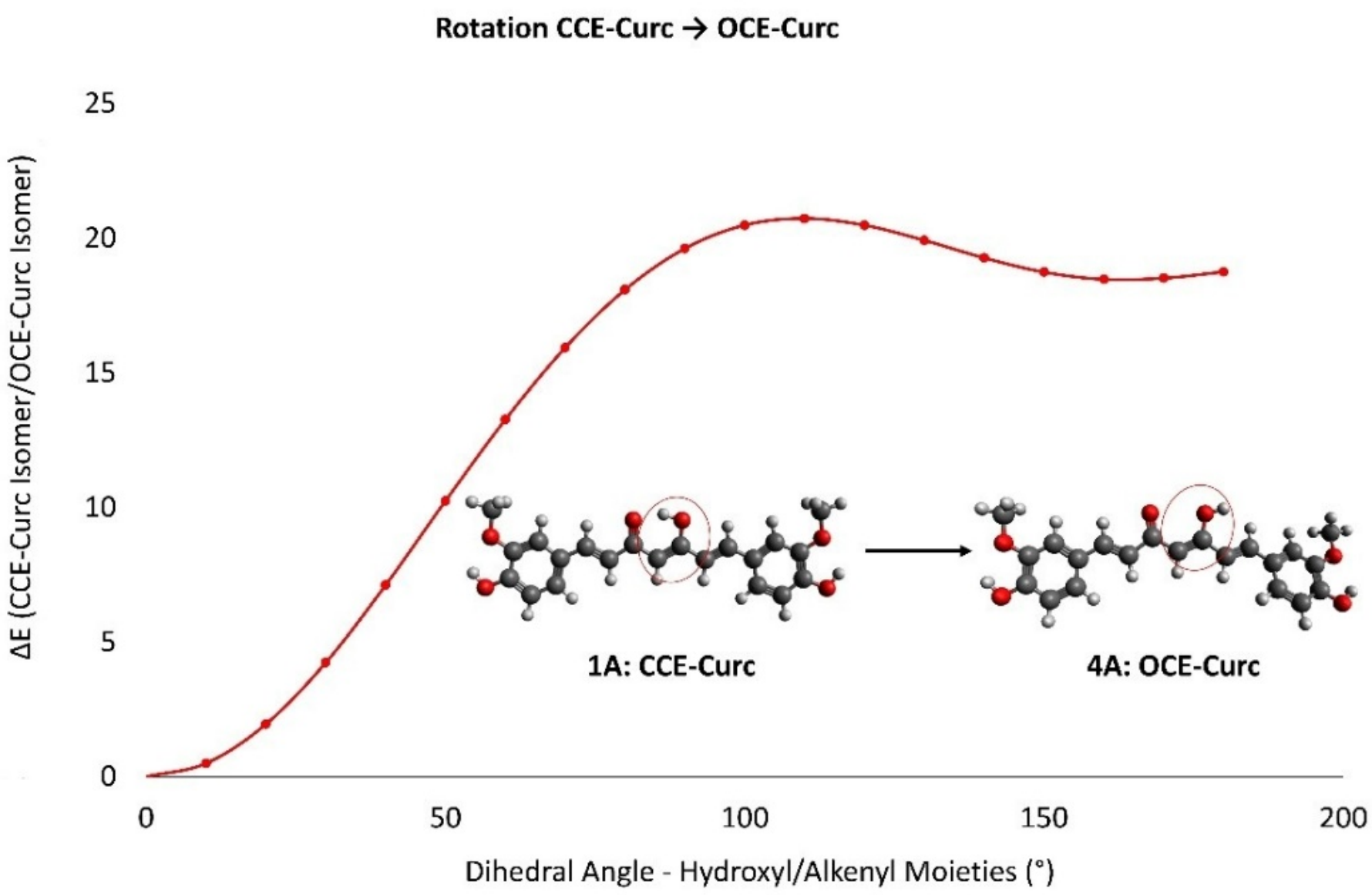

Figure S1. Relaxed scan of the Potential Energy Surface (PES) of Enol Curcumin from Closed Cis Enol Curcumin Isomer to Open Cis Enol Curcumin one (see Figure 2 in the main text), obtained by varying the dihedral angle between the hydroxyl and the alkenyl moieties of the keto-enol group by 10° from 0° to 180°. The dihedral angle is defined by atoms enclosed in the red circles shown in the Figure. The energies of each conformational isomer were normalized with respect to the energy of Closed Cis Enol Curcumin (∆E(CCE-Curc Isomer/OCE-Curc Isomer) and reported in kcal/mol. The dihedral angles are reported in degrees.

Not surprisingly, both calculations show that the Closed Cis molecule, characterized by keto-enol intramolecular H-bonding, is the most stable isomer with energy barriers and conformational energy differences respectively around 20 kcal/mol and 15-18 kcal/mol. The latter substantially reflects the strength of the keto-enol intramolecular H-bond which, in both cases, breaks following the conformational change. In fact, the binding energy of intramolecular H-bonds in neutral and medium-sized molecules such as curcuminoids usually ranges from around 5 kcal/mol up to around 15 kcal/mol.[1–4] This comes as no surprise though, considering the peculiar curcuminoids' structure. In fact, the intramolecular H-bond under consideration is associated with a keto-enol group which results from the tautomerism of a β-diketone, lying on a π-conjugated skeleton. Therefore, it falls into the category of Resonance-Assisted H-bonds (RAHBs) introduced by Gilli and coworkers in the late 80s.[5]

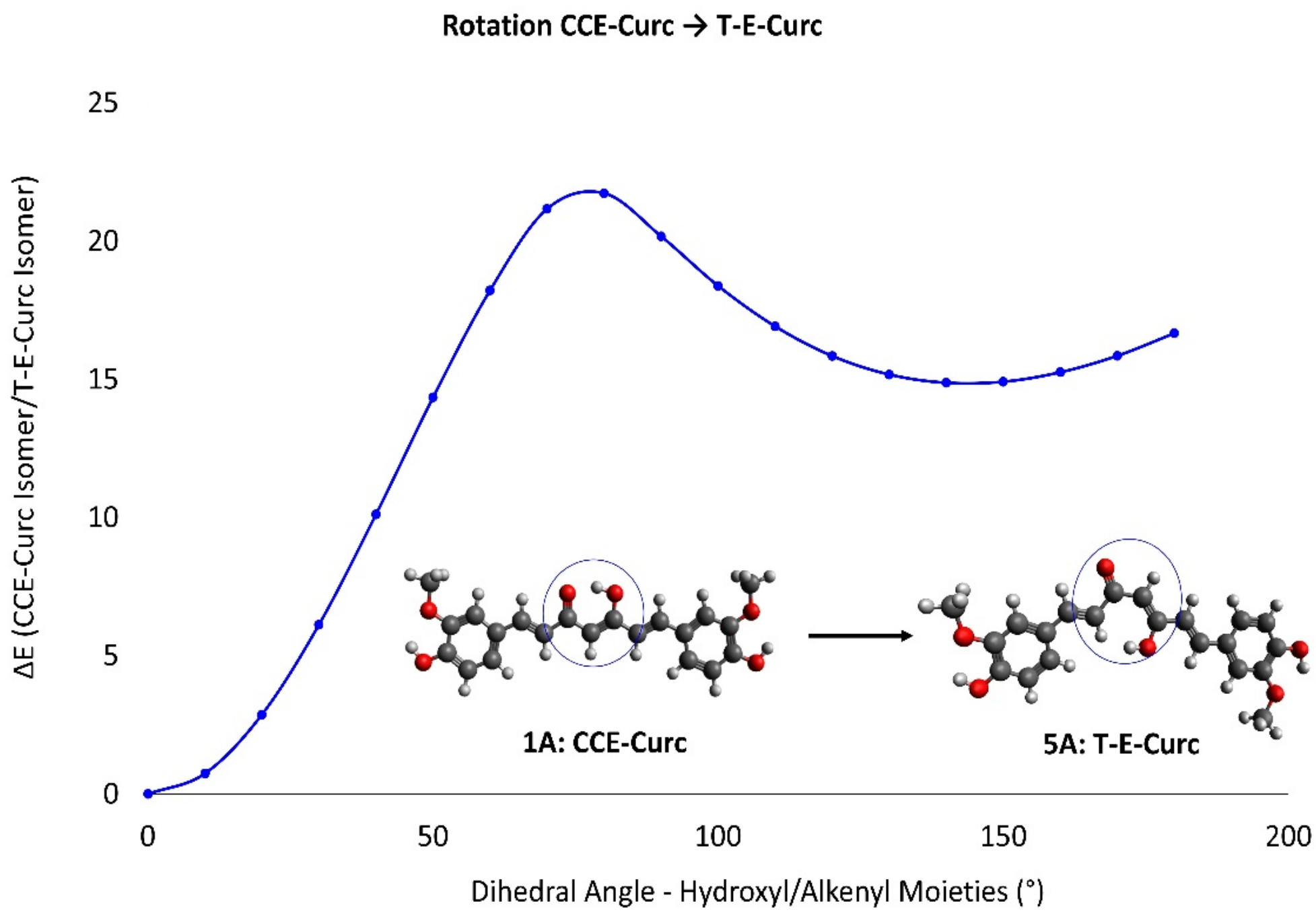

Figure S2. Relaxed scan of the Potential Energy Surface (PES) of Enol Curcumin from Closed Cis Enol Curcumin Isomer to Trans Enol Curcumin one (see Figure 2 in the main text), obtained by varying the dihedral angle between the carbonyl and the hydroxyl moieties of the keto-enol group by 10° from 0° to 180°. The dihedral angle is defined by atoms enclosed in the blue circles shown in the Figure. The energies of each conformational isomer were normalized with respect to the energy of Closed Cis Enol Curcumin (ΔE(CCE-Curc Isomer/OCE-Curc Isomer) and reported in kcal/mol. The dihedral angles are reported in degrees.

In fact, according to these researchers, the strengthening of H-bonds in conjugated π-systems results as a direct consequence of the extra resonance stabilization effect characterizing these compounds. Besides, in this specific case, the formation of the keto-enol intramolecular H-bond gives place to a pseudo-aromatic six-membered ring which might further stabilize the bond (Figure S3).

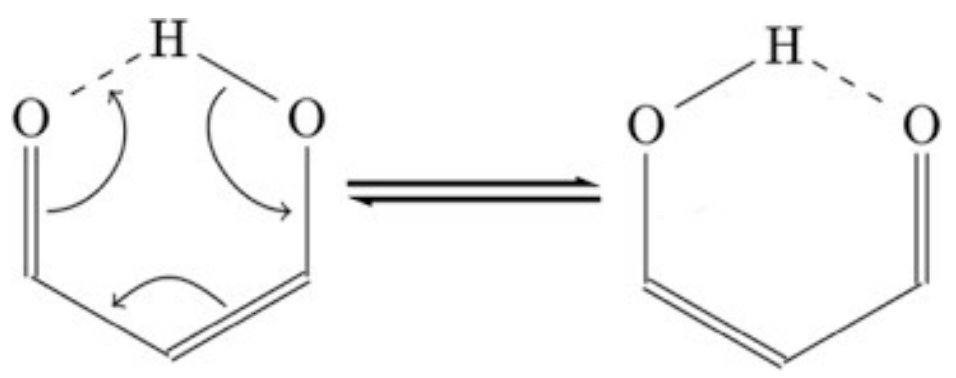

Figure S3. Resonance Assisted H-Bond (RAHB) mechanism in keto-enol tautomeric structure of β-diketones.

This trend is confirmed by the proper conformational analysis in Figure S4 which shows the energy difference between the optimized geometry of compound 1A:CCE-Curc and the corresponding values associated with its isomers (GROUP A, Figure 2 in the main text). Interestingly, the most stable isomer is compound 2A:CCE-Curc-Conf. Since the energy difference with respect to compound 1A:CCE-Curc is extremely low, of the order of 0.1 kcal/mol, from this point on we will refer to compound 1A:CCE-Curc as the reference molecule to discuss curcuminoids radicalization, considering it as the most stable and abundant curcumin's isomer.

Due to the cleavage of the intramolecular H-bond, the Trans Diketo Curcumin (compound 3A:T-DIK-Curc) results considerably more stable than both Open Cis and Trans Enol Curcumin (compounds 4A:OCE-Curc and 5A:T-E-Curc, respectively) which show conformational energies 2-3 times higher than the T-DIK-Curc's one. However, it is important to bear in mind that such energies do not merely depend on intramolecular H-bond energetic contribution but also on the overall structural relaxation. In this sense, it is worth noticing that 4A:OCE-Curc basically retains the 1A:CCE-Curc geometry while both 5A:T-E-Curc and 3A:T-DIKCurc undergo significant distortion, deviating from the peculiar planar geometry of the reference isomer. The comparison of the conformational energies of 5A:T-E-Curc, 3A:T-DIK-Curc and 4A:OCE-Curc with respect to 1A:CCE-Curc also suggests that out-of-plane distortions might significantly stabilize curcuminoids when intramolecular keto-enol H-bond cannot be formed.

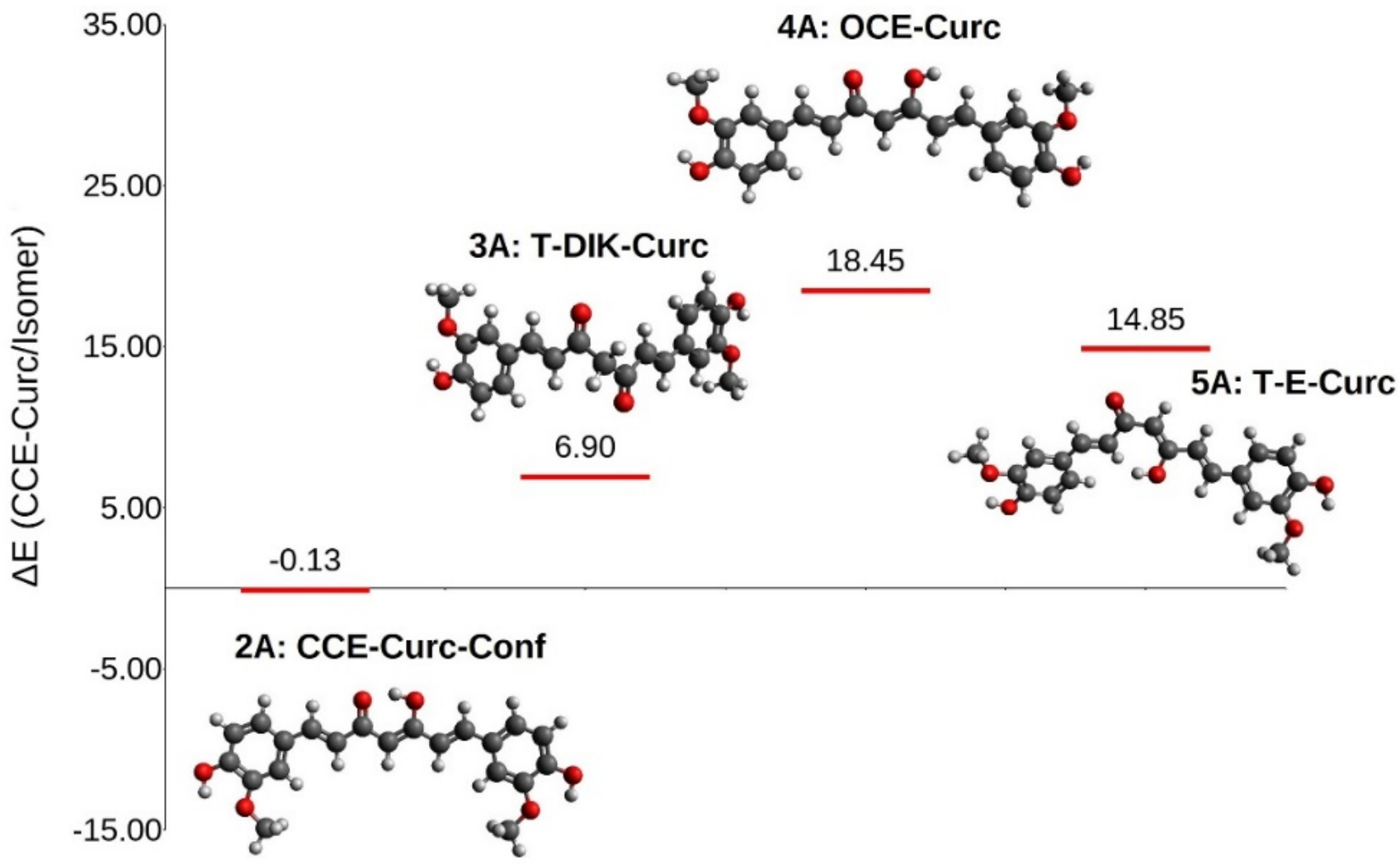

Figure S4. Relative thermodynamic stability of curcumin isomers with respect to Closed Cis Enol Curcumin (Compound 1A: CCE-Curc, see Figure 2 in the main text), evaluated in term of energy difference (ΔE(CCE-Curc/Isomer)) between the corresponding total energies associated with the optimized ground states of the molecules. All values are reported in kcal/mol.

In order to test the robustness of these results, we investigated both the convergence with respect to the basis set and solvent effect which was at first simulated by employing the Polarizable Continuum Model (PCM). More specifically, we performed single point energy calculations on the previously optimized structures using the Pople 6-311G++(d,p) basis set, featuring diffuse functions on both heavy atoms and hydrogens, and the implicit solvation model to simulate the molecular energy in the presence of methanol and acetonitrile which were employed in the experimental investigation (*vide infra*). The conformational energy change ΔΔE has been calculated according to Equation 2.

$$\square\square E = \square E(\mathrm{CCE} - \mathrm{Curc/Isomer})_{\mathrm{B3LYP/6-311G(d,p)}} - \square E(\mathrm{CCE} - \mathrm{Curc/Isomer})_{\mathrm{ACM}} \qquad (2)$$

In Equation 2, ACM stands for Alternative Computational Method.

A visual comparison of the ΔΔE values retrieved for all isomers is offered in Figure S5.

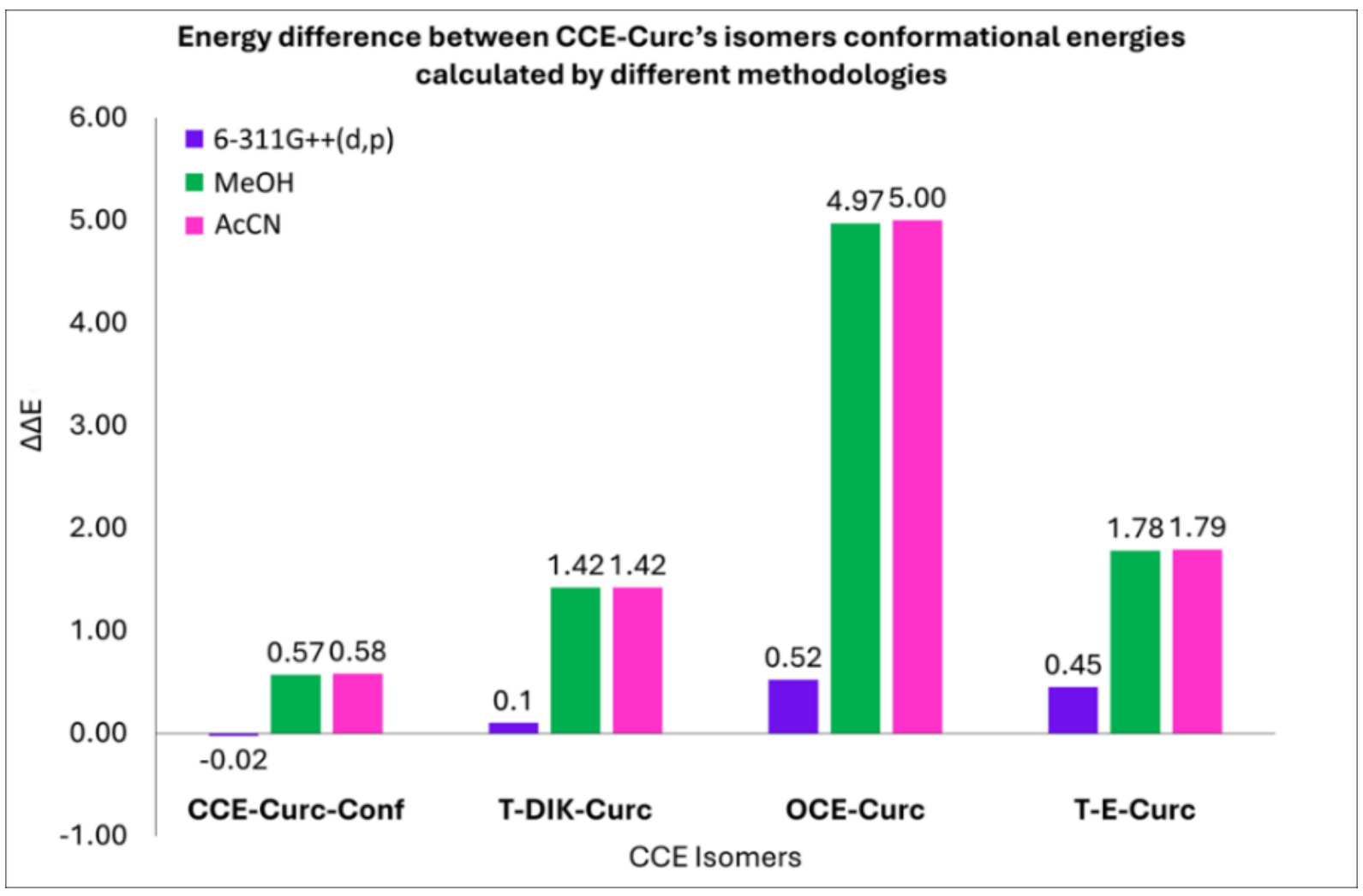

Figure S5. Conformational energy change for all Closed Cis Enol (CCE) Curcumin's isomers (ΔΔE, Equation 2) calculated by employing three different methods: B3LYP/6-311G++(d,p) (violet bars), B3LYP/6-311G(d,p) in methanol (PCM level) (green bars) and B3LYP/6-311G(d,p) in acetonitrile (PCM level) (pink bars). All data are reported in kcal/mol.

Interestingly, in all cases except the conformational energy change for 2A:CCE-Conf's due to the introduction of diffuse functions, both the latter and the solvent effect employment determines a slight decrease in the energy of higher lying conformers.

This indirectly suggests that more accurate computational methodologies (e.g. including a polarizable solvent model) provide less strong keto-enol intramolecular H-bonds, especially when the higher lying isomer increases its local polarity by exposing the acidic proton of the enolic group to a polarizable environment. Quite surprisingly, changes are extremely small when diffuse functions are added to the reference basis set (i.e. below 1 kcal/mol). In order to further investigate the convergence of these results, the eventual Basis Set Superimposition Error (BSSE) associated with the keto-enol intramolecular H-bond of the reference 1A:CCE-Curc molecule was also calculated using the counterpoise correction module of Gaussian09. The latter method, however, allows to calculate the BSSE associated with interacting atoms belonging to distinct monomers; we were, thus, forced to build a model of compound 1A:CCE-Curc consisting of independent enol and ketone moieties (Figure S6).

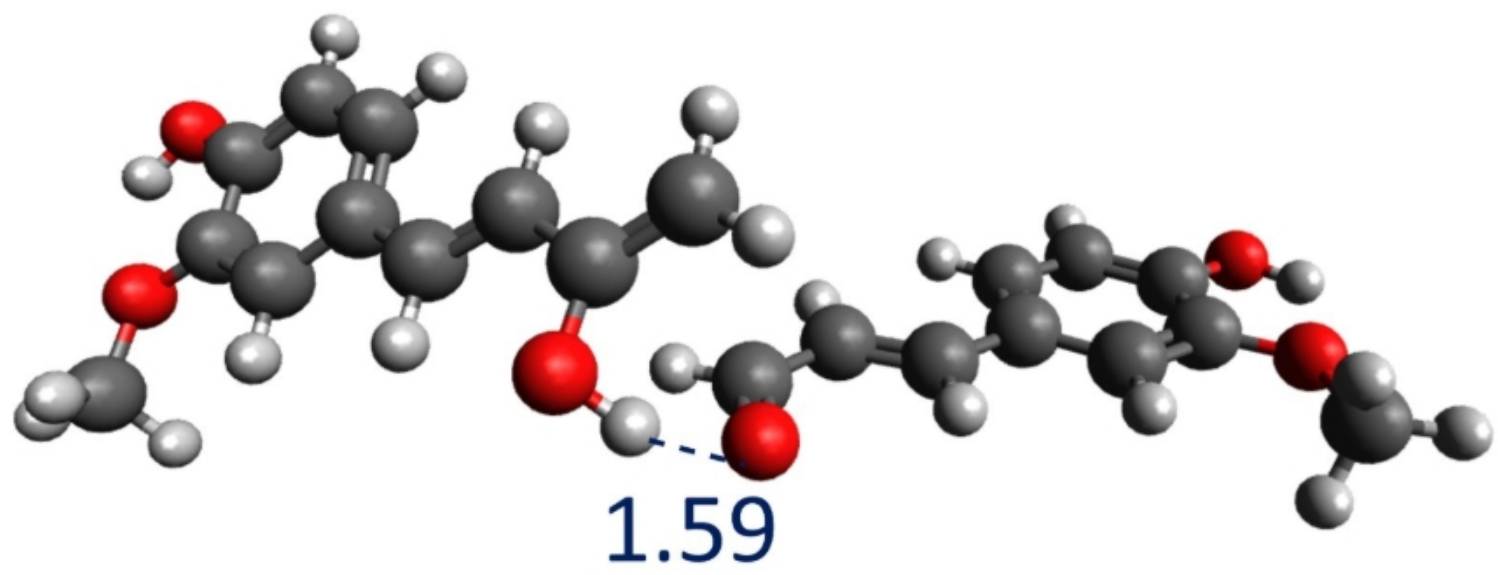

Figure S6. Model structure of compound 1A:CCE-Curc consisting of an enol and a ketone moiety. The distance between the hydrogen atom of the enol group and the oxygen atom of the ketone group reported in Å in the Figure corresponds to the distance between the same atoms in the optimized structure of the compound.

The distance between the hydrogen atom of the enol group and the oxygen atom of the ketone group was set at 1.59 Å (as in the optimized structure of compound 1A:CCE-Curc) and kept fixed during geometry relaxation. In the end, the BSSE resulted to be extremely low (around 0.7 kcal/mol), and it is thus not expected to affect the isomers' conformational energies discussed above (Figure S4). Conversely, the effect of the solvent appears more marked, with changes in conformational energy estimates ranging from around 1.5 kcal/mol in case of 3A:T-DIK-Curc to around 5 kcal/mol in case of 4A:OCE-Curc. Note that methanol and acetonitrile have similar dielectric constants at 20° (33.0 and 37.5, respectively); consequently, the simulations by the Implicit Solvent approach return comparable results (Figure S5). Notwithstanding the impact of higher theory levels, the relative conformational energies remain quite high, so confirming the particularly strong character of the keto-enol intramolecular H-bond. This said, one may be left to wonder whether including solvent molecules explicitly may decrease further the energy gaps, given the important effect demonstrated by the implicit solvent model. With this in mind, the conformational energies were recalculated in the presence of two acetonitrile molecules or including two or four methanol molecules; in all cases, geometries were fully optimized starting from aggregates with the solvent molecules in the vicinity of the keto-enol group. The optimized geometries are reported in Figures S7, S8 and S9, while the corresponding conformational energies are shown in Figure S10.

As expected, both methanol and acetonitrile molecules interact between themselves and with the solute by forming either relatively strong H-bonds (bond lengths around 1.8-1.9 Å) or dipole-dipole intermolecular bonds (bond lengths 2.3-2.6 Å). Interestingly, the H-bond distances shown in Figures S7, S8 and S9 suggest that these curcuminoids are more prone to accept H-bonds than to donate them. In fact, the solvent-solute H-bonds involving the oxygen atoms of the keto-enol group as bond acceptors are on average around 0.5 Å shorter than the bonds involving hydrogen atoms of the solute as bond donors. Although a more in-depth analysis should be conducted to properly assess this trend, the latter might be due to the peculiar structure of curcuminoids. In fact, the hydroxyl groups of curcuminoids which might act as H-bond donors are either involved in intramolecular H-bonds (e.g. in compounds 1A:CCE-Curc and 2A:CCE-Curc-Conf) or present a limited conformational flexibility, so that it becomes energetically more convenient to have methanol molecules donating H-bonds.

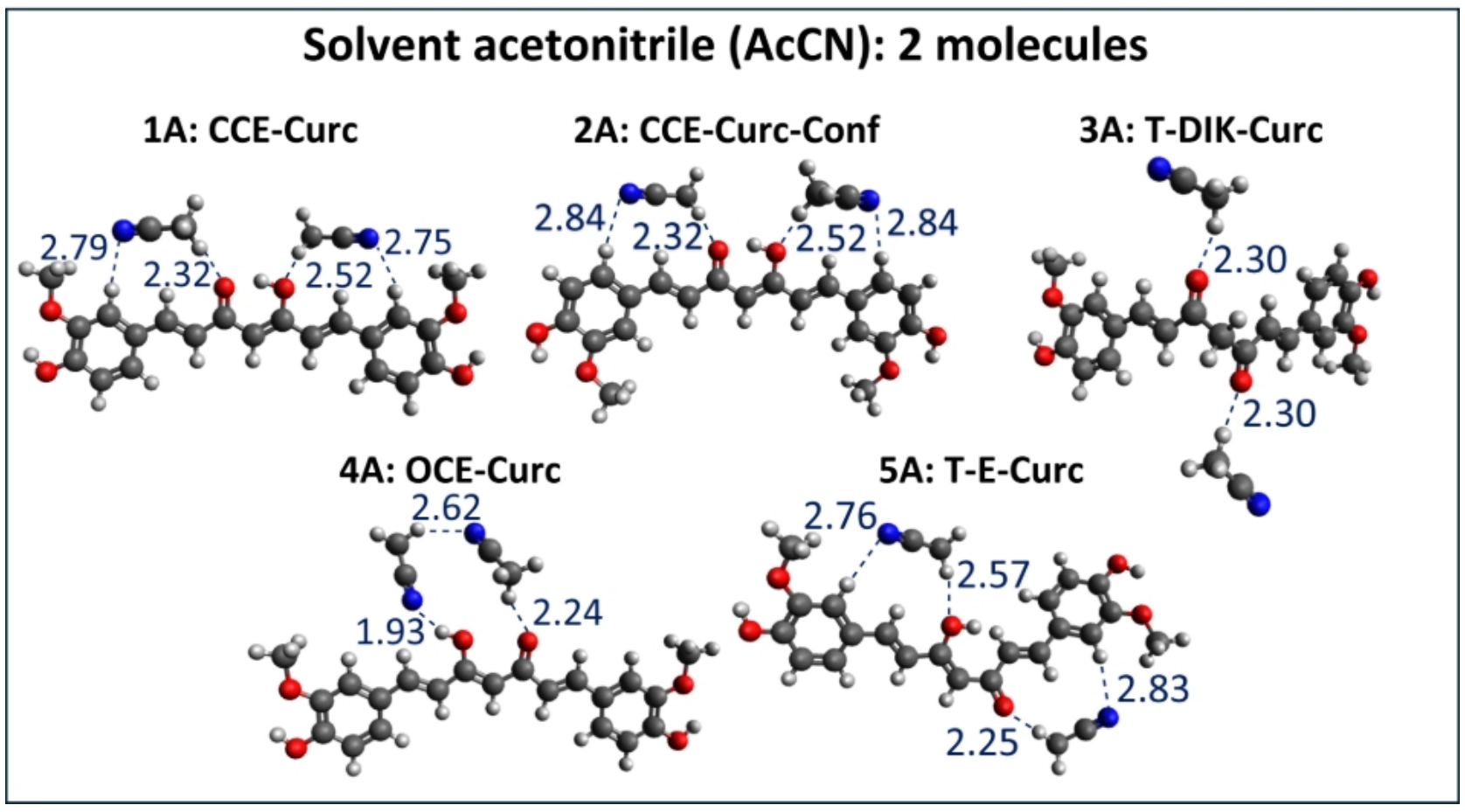

Figure S7. Optimized geometries of Closed Cis Enol (CCE) Curcumin's isomers in the presence of 2 molecules of acetonitrile. The most relevant solvent-solute and solvent-solvent bond distances in Å are also shown.

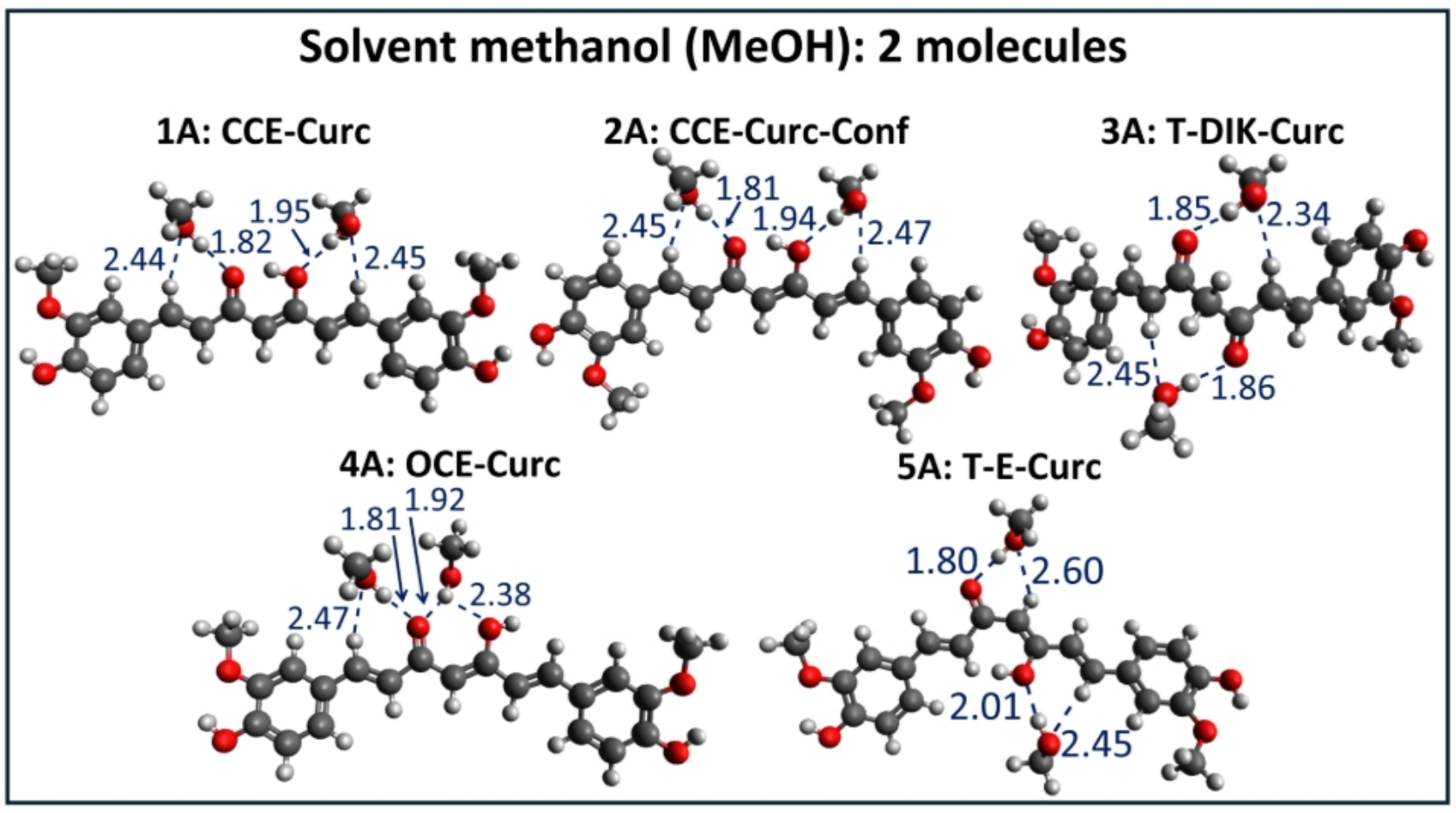

Figure S8. Optimized geometries of Closed Cis Enol (CCE) Curcumin's isomers in the presence of 2 molecules of methanol (MeOH). The most relevant solvent-solute and solvent-solvent bond distances in Å are also shown.

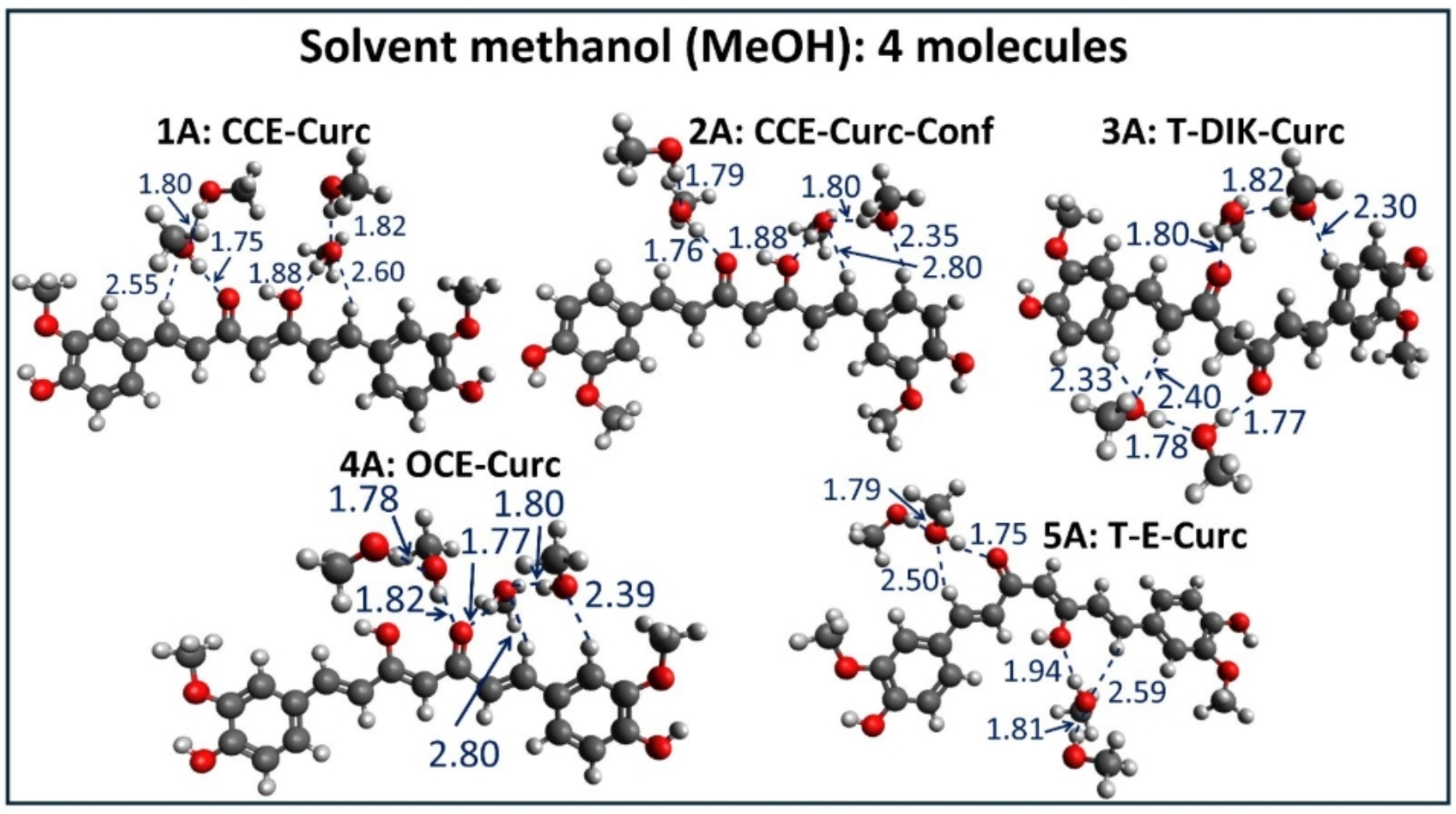

Figure S9. Optimized geometries of Closed Cis Enol (CCE) Curcumin's isomers in the presence of 4 molecules of methanol (MeOH). The most relevant solvent-solute and solvent-solvent bond distances in Å are also shown.

These fragments are particularly thermodynamically stable and thus less prone to interact with solvent molecules. In any case, both the solvent-solvent and solvent-solute interactions contribute to stabilize the isomers (Figure S10). This stabilization is particularly evident for both compounds 3A:T-DIK-Curc and 4A:OCE-Curc with 4 molecules of methanol. In fact, in the first case

the difference in conformer energy reduces by around the 90% while in the second one by around the 60% with respect to the values shown in Figure S4. On the contrary, such interactions do not significantly affect the conformational energy of compounds 1A:CCE-Curc-Conf and 5A:T-E-Curc, which is reduced only by 1-2 kcal/mol.

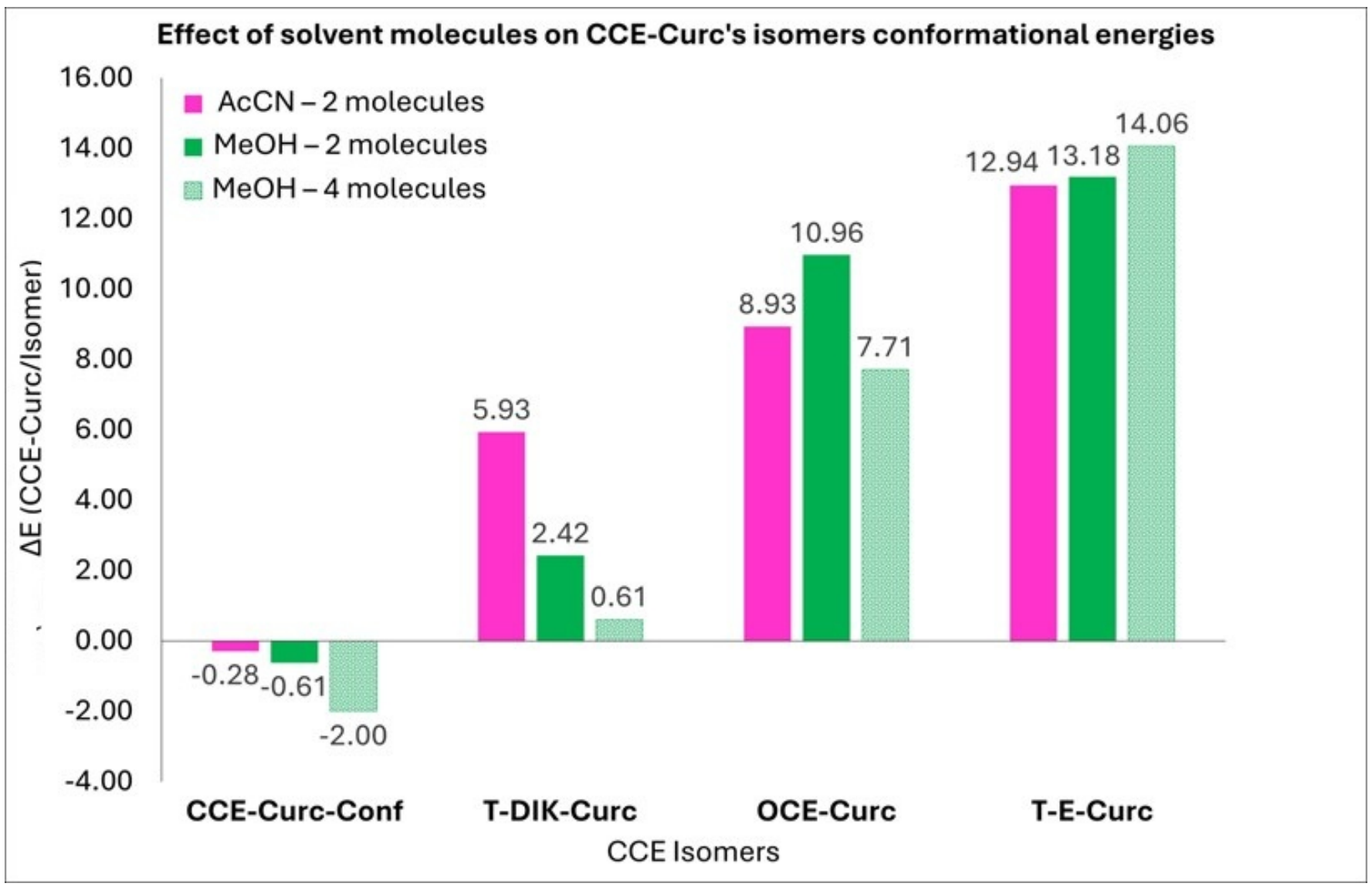

Figure S10. Conformational energies of CCE-Curc's isomers in the presence of 2 molecules of acetonitrile (AcCN), 2 molecules of methanol (MeOH) and 4 molecules of methanol.

In the end, these new results are consistent with those previously obtained (Figure S5) despite the more significant decrease of the conformational energy differences in presence of solvent molecules; in other words, they bring additional support to the particularly high thermodynamic stability of keto-enol intramolecular H-bond in curcuminoids.

To explore whether the CCE/OCE conversion is made somewhat more facile also from the kinetic viewpoint by the presence of explicit solvent molecules capable of donating/accepting hydrogen bonds, we have also explored the energy surface of a trimeric systems involving two water molecules using the same order parameter (i.e. the dihedral angle) exploited in Figure S1; we chose this specific system involving water, rather than methanol or acetonitrile, to maximize the number of possibly donated/accepted hydrogen bonds, hence the impact on the process energetics. The results are reported in Figure S11, and indicate that the barrier estimated in vacuum may be substantially reduced by the presence of solvent molecules to, roughly, 8 kcal/mol.

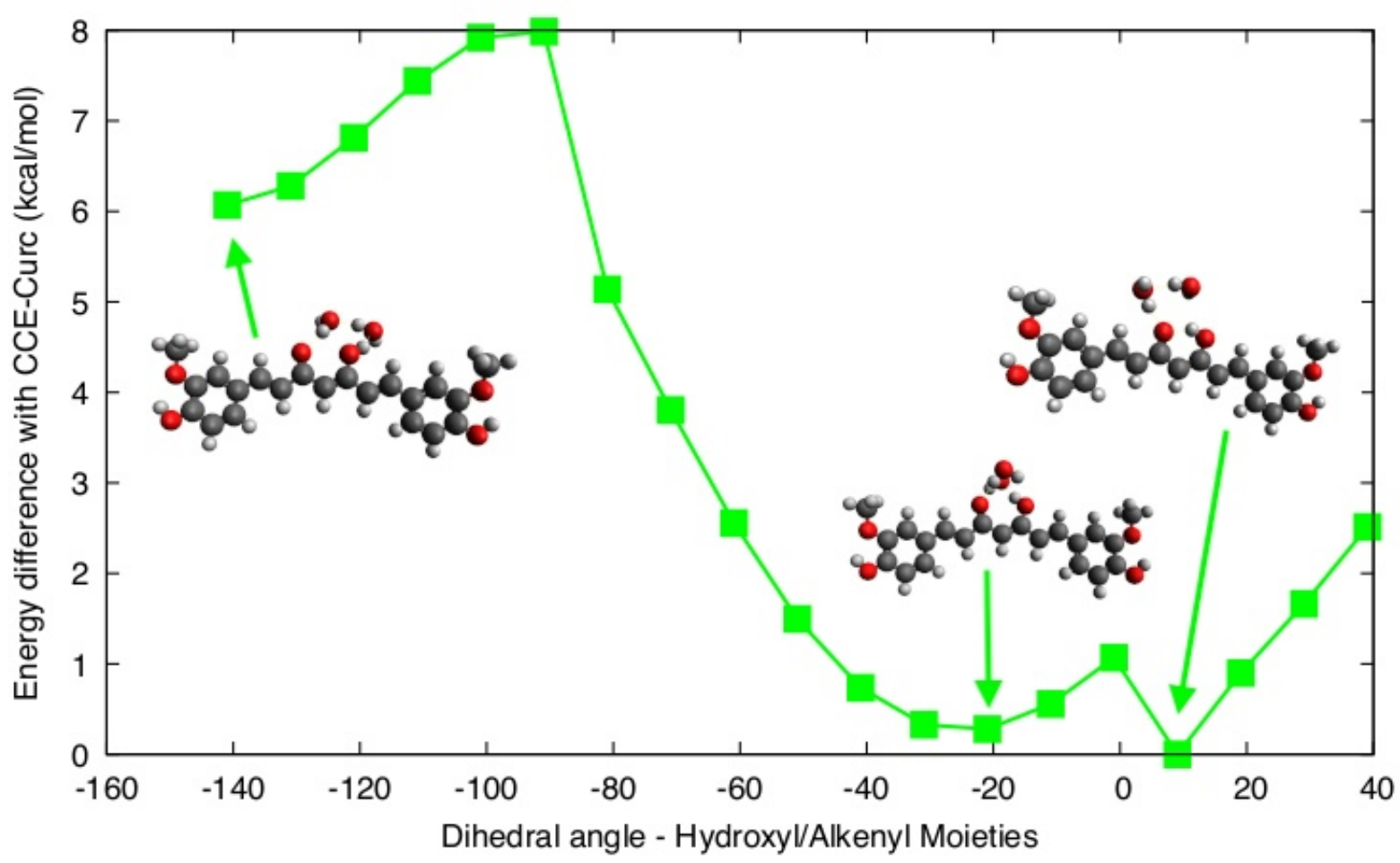

Figure S11. Relaxed scan of the Potential Energy Surface (PES) of Enol Curcumin with two water molecules from Closed Cis Enol Curcumin Isomer to Open Cis Enol Curcumin one, obtained by varying the dihedral angle between the hydroxyl and the alkenyl moieties of the keto-enol group. The dihedral angle is defined by atoms enclosed in the red circles shown the Figure S1. The energies of each conformational isomer were normalized with respect to the lowest energy conformer, i.e. Closed Cis Enol Curcumin and reported in kcal/mol. The dihedral angles are reported in degrees.

Despite this finding, the energy profiles confirm that CCE-Curc still remains the most stable isomer and that the CCE/OCE conversion ought to be slow. This notwithstanding, the results also clearly indicate that several local minima may be encountered while driving a specific order parameter (e.g. dihedral angle) as a consequence of molecules reorientation, making the overall study of conformational changes in presence of explicit solvent quite complicated.

S2. Effects of semi-empirical corrections for the dispersion interactions

Although literature DFT results including Grimme's D3 correction for conformational energies of curcuminoids are available for comparison with our results (*vide*, for instance, Madinah *et al.*[6]), we nevertheless assessed the effect of this correction on selected quantities for a limited set of representative compounds relevant to the present work.

First, we recalculated the relative energies of 4A:OCE-Curc and 3A:T-DIK-Curc with respect to 1A:CCE-Curc (taken as the zero of energy as before). These compounds were selected because they allow us to probe the effect of D3 correction on different structural features of curcumin and energy scales. More specifically, 4A:OCE-Curc represents the higher-energy conformer in which the resonance-assisted hydrogen bond (RAHB) stabilizing 1A:CCE-Curc is absent, whereas 3A:T-DIK-Curc was selected as the lowest-energy structure featuring a diketone rather than the keto-enol tautomer. Importantly, upon inclusion of Grimme's correction the relative energies of 4A:OCE-Curc and 3A:T-DIK-Curc become 18.66 and 6.35 kcal/mol, respectively, differing by only 0.21 and −0.55 kcal/mol from the values reported in Figure S4 and in the main text.

A similar test was also carried out for the bond dissociation energy (i.e. hydrogen loss) shown in Figure 2 of the main text. In particular, we considered the O-H bond breaking of both enolic and phenolic moieties in 1A:CCE-Curc, and the C-H bond breaking of the methylene group in 3A:T-DIK-Curc. In all cases, the D3 correction increased the BDEs by roughly 1.2 kcal/mol. This increase is consistent with the additional stabilization provided by the dispersion correction, although its magnitude remains small.

Overall, these tests showed that the inclusion of the D3 correction has a negligible impact on the relative energetics of curcumin isomers and on bond-breaking processes, with energy differences well below or on the order of chemical accuracy (1 Kcal/mol). We therefore retained the computational protocol without the D3 correction throughout the study.

References for the Supporting information section:

1 C. L. Perrin and J. B. Nielson, "STRONG" HYDROGEN BONDS IN CHEMISTRY AND BIOLOGY, *Annu. Rev. Phys. Chem.*, 1997, **48**, 511–544.

2 K. Szalewicz, in *Encyclopedia of Physical Science and Technology*, Elsevier, 2003, pp. 505–538.

3 S. J. Grabowski, *Understanding Hydrogen Bonds*, The Royal Society of Chemistry, 2020.

4 M. Shahamirian and S. M. Azami, Strong intramolecular hydrogen bonding in confined amino acids, *J. Mol. Graph. Model.*, 2021, **106**, 107913.

5 G. Gilli, F. Bellucci, V. Ferretti and V. Bertolasi, Evidence for resonance-assisted hydrogen bonding from crystal-structure correlations on the enol form of the .beta.-diketone fragment, *J. Am. Chem. Soc.*, 1989, **111**, 1023–1028.

6 R. Madinah, F. Rusydi, R. N. Fadilla, V. Khoirunisa, L. S. P. Boli, A. G. Saputro, N. H. Hassan and A. Ahmad, First-Principles Study of the Dispersion Effects in the Structures and Keto–Enol Tautomerization of Curcumin, *ACS Omega*, 2023, **8**, 34022–34033.
